# In situ mapping of activity distribution and oxygen evolution reaction in vanadium flow batteries

**DOI:** 10.1038/s41467-019-13147-9

**Published:** 2019-11-21

**Authors:** Kaijie Ma, Yunong Zhang, Le Liu, Jingyu Xi, Xinping Qiu, Tian Guan, Yonghong He

**Affiliations:** 1 Institute of Green Chemistry and Energy, Graduate School at Shenzhen, Tsinghua University, Shenzhen, 518055 China; 2 Department of Physics, Tsinghua University, Beijing, 100084 China; 3 Shenzhen Key Laboratory for Minimal Invasive Medical Technologies, Institute of Optical imaging and Sensing, Graduate School at Shenzhen, Tsinghua University, Shenzhen, 518055 China; 4 Key Lab of Organic Optoelectronics and Molecular Engineering, Department of Chemistry, Tsinghua University, Beijing, 100084 China

**Keywords:** Imaging studies, Sensors, Batteries, Reaction kinetics and dynamics

## Abstract

Understanding spatial distribution difference and reaction kinetics of the electrode is vital for enhancing the electrochemical reaction efficiency. Here, we report a total internal reflection imaging sensor without background current interference to map local current distribution of the electrode in a vanadium redox flow battery during cyclic voltammetry (CV), enabling mapping of the activity and reversibility distribution with the spatial resolution of a single fiber. Three graphite felts with different activity are compared to verify its feasibility. In long-term cyclic voltammetry, the oxygen evolution reaction is proved to enhance activity distribution, and homogeneity of the electrode and its bubble kinetics with periodic fluctuation is consistent with the cyclic voltammetry curve, enabling the onset oxygen evolution/reduction potential determination. Higher activity and irreversibility distribution of the electrode is found in favor of the oxygen evolution reaction. This sensor has potential to detect in situ, among other processes, electrochemical reactions in flow batteries, water splitting, electrocatalysis and electrochemical corrosion.

## Abstract (web summary)

Redox flow batteries are attractive for large-scale energy storage, but electrode activity should be better understood to improve electrochemical performance. Here the authors map the surface activity distribution of a vanadium redox flow battery electrode with spatial resolution of a single fiber.

## Introduction

As a large-scale stable energy storage technology to tackle the intermittent supply of renewable energy, redox flow batteries are deserving attention for the superiority of reliable and sustainable power supply^1,2^. Vanadium redox flow batteries (VFB, VRB, VRFB) have advantages of unparalleled cycle life for no cross-contamination issues (the same vanadium ions with different valence states as active species), easy to scale for high capacity of a single battery and environmentally friendly for electrolyte recycling, thus, exhibiting promising prospect of industrialization^3,4^. Nowadays, the pursuit of next-generation VFBs with high electrochemical performance (power density, energy density, long-term stability, etc.) motivates researchers to focus on the effective amelioration of electrode^5^, membrane^6^, electrolyte^7–9^, and cell structure design^10^. Conventional methods to evaluate their effects on electrochemical performance can only provide average information of the whole cell, including cyclic voltammetry (CV)^11^, electrochemical impedance spectroscopy^12^, charge-discharge test^13^, polarization curves^14^, long-term cycling test^15^, and so on. However, the spatial distribution difference of the cell is also the vital point affecting the performance of energy efficiency, charge-discharge capacity, polarization^16–19^. Since electrode is the primary active reaction site for the redox couples, the activity, conductivity, polarization and reversibility distribution of the electrode should be considered to fully participate in the reaction of active species on the electrode.

As the mainstream electrode material of VFBs, graphite felt provides multiple active reaction sites for its porous structures with high area to volume ratio and maintains stability in the strongly oxidizing and acidic environment^20^. To further improve the electrochemical performance of the graphite felt, a series of modification methods, such as thermal treatment^21^, electrocatalyst introduction^22^, doping^23^, and electrode compression^24^ are utilized to enhance the reaction activity and reduce resistance and polarization. However, spatial distribution of active species introduced by above methods should be characterized to ensure fully utilization of active species in the battery^25^. Otherwise, non-uniform distribution of active sites may cause different local reaction rates and therefore aggravate electrochemical polarization and concentration polarization. Despite that the whole performance effected by the non-uniform distribution can be detected by the polarization curve^26^, it cannot in situ determine the local distribution information (active site distribution, additive distribution, porosity, etc). Besides, activity difference rendered by poor charge transfer on the electrode would lead to large overpotentials and thus oxygen evolution reaction (OER) occurs^27^. Efforts have been payed either to investigate how the electrocatalyst affects OER kinetics^28,29^ or directly image the growth of oxygen bubbles^30,31^. As electrode corrosion caused by the OER might occur, the relationship between the local activity distribution and the oxygen bubble location on the electrode should be determined to inhibit the OER for cell performance optimization.

Local current distribution is an intuitive indicator to characterize activity distribution and reaction kinetics of the porous electrode. Uniform current distribution demonstrates the consistent reaction rate. Traditional in situ measurement techniques for current distribution, such as potential probes^26^, shunt-resistors^32^, and printed circuit boards^18^ are based on the Ohm’s law, whose detection results may be affected by the cell. As limited spatial resolution of the above techniques, only the flow rate and other macroscopic factors can be quantified on the current distribution^33^. Optical imaging detection method with high spatial resolution and sensitivity, high throughput and minimal interference is a promising option to detect the local current density distribution caused by active reaction site, additive distribution and other microscopic factors^34^.

Optical imaging sensors such as surface plasmon resonance imaging (SPRi) not only provide spatially resolved microscopic imaging, but also allow simultaneous detection of real-time kinetic process. SPRi sensor is a powerful tool for mapping physical quantities like local electrochemical current^34–37^ and measuring the reaction variation process^38–42^. Similar to SPRi sensors in detecting surface refractive index distribution, imaging sensors based on total internal reflection (TIRi), which is an ancient method but has new development in recent years^43,44^, have potential in mapping local current distribution of VFBs for the following reasons. First, due to the lack of gold film, there is no interference of Faraday current from the gold film to the electrochemical process of the measured substance, which occurs in SPRi sensors^41^. Second, the problem that the gold film of the SPRi sensor is easy to fall off in the strongly oxidizing and acidic VFB operating environment is also avoided (Supplementary Fig. 1). Third, coupling element (prism, etc.) used in the TIRi sensor is inactive in surface reaction and has the feasibility of long-term utilization.

Herein, we present an unprecedented method of imaging surface activity distribution of a VFB electrode by mapping the local current density distribution with the TIRi sensor, which not only images the location of graphite felt fibers, but also detects their local electrochemical reaction kinetics. By CV, a graphite felt electrode in a three-electrode electrochemical cell is imaged by the TIRi sensor and simultaneously the time-varying refractive index of the positive electrolyte is recorded by the sensor to acquire the local current density. The peak oxidation/reduction current densities, their ratio and the peak potential separation value are obtained to map the local activity and reversibility distribution. The generated bubbles from oxygen evolution reaction on the electrode surface are imaged in real time to explore the relationship between the local activity distribution and bubble location distribution. This TIRi sensor has potential to in situ detect the electrochemistry kinetics distribution of the electrode in flow batteries, water splitting, electrocatalysis and electrochemical corrosion for performance optimization.

## Results

### TIR imaging of graphite felt fibers

**Fig. 1:**
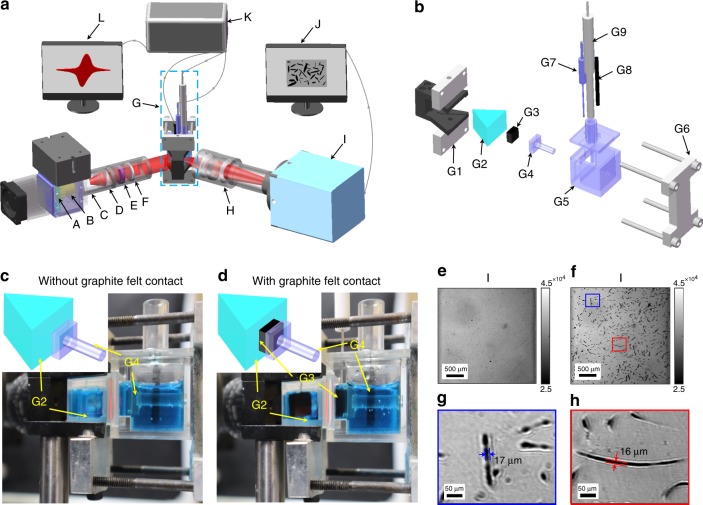
Total internal reflection imaging (TIRi) sensor system for current density detection of electrode. **a** Schematic of the TIRi sensor system. A: LED; B: objective lens; C: aperture; D: achromatic lens; E: bandpass filter; F: linear polarizer; G: reservoir-coupled sensor module (RCS); H: imaging lens; I: charge coupled device (CCD); J: computer for receiving images from the CCD; K: electrochemical workstation (EW); L: computer for receiving cyclic voltammetry (CV) curves from the EW. **b** Expanded view of the RCS in **a**. G1: prism holder; G2: prism; G3: graphite felt; G4: strut; G5: fluid reservoir; G6: reservoir holder; G7: platinum electrode; G8: graphite rod; G9: saturated calomel electrode. **c** Snapshot and schematic (inset) of RCS without graphite felt contact. **d** Snapshot and schematic (inset) of RCS with graphite felt contact. **e** Image captured by CCD in case **c**. **f** Image captured by CCD in case **d**. **g, h** Enlarged views of rectangular regions marked in **f**.

Graphite felt is the prevalent electrode material in VFBs. To verify the feasibility of the TIRi sensor for mapping the activity and reversibility distribution of electrodes, graphite felt (GF), thermal activated graphite felt (TGF) and porous graphite felt (PGF) are used as the electrodes in this work. SEM images reveal that the surfaces of GF and TGF are smooth while that of PGF has many holes. Their typical diameter is about 13 μm (Supplementary Fig. 2). The home-built TIRi system in Fig. 1a, b was utilized to image the electrode and detect the kinetics of vanadium ions during the electrochemical process (described in Methods). To verify the TIR image of graphite felt fibers, two situations (insets in Fig. 1c, d) of the reservoir-coupled sensor module (RCS) without and with graphite felt contact are compared in Fig. 1c, d. Due to different reflected light intensity at the prism interface in contact with the graphite felt and electrolyte (refractive index: $n_{\mathrm{graphite}} = 2.8363$; $n_{\mathrm{electrolyte}} = 1.3566$; $n_{\mathrm{prism}} = 1.75$ at the wavelength of 632.8 nm)^45^, the fibers are visualized in Fig. 1f with graphite felt contact while the image with homogeneous intensity distribution is the situation without graphite felt contact in Fig. 1e. The enlarged views of two regions marked as red and blue rectangles in Fig. 1f demonstrate the traces of fibers with the diameter of ~16 μm in Fig. 1g, h, which is slightly larger than that (13 μm) in SEM images (Supplementary Fig. 2) owing to the diffraction effect. Their relevance of the fiber diameters further confirms the traces in TIR images are graphite fibers. Since the reflected light intensity varies quantitatively with the refractive index variation, and the detection depth of the reflected light is consistent with the penetration depth (~938 nm) of the evanescent wave, the TIR image reveals the RI of the contact interface.

### Local current density mapping by the TIRi sensor

**Fig. 2:**
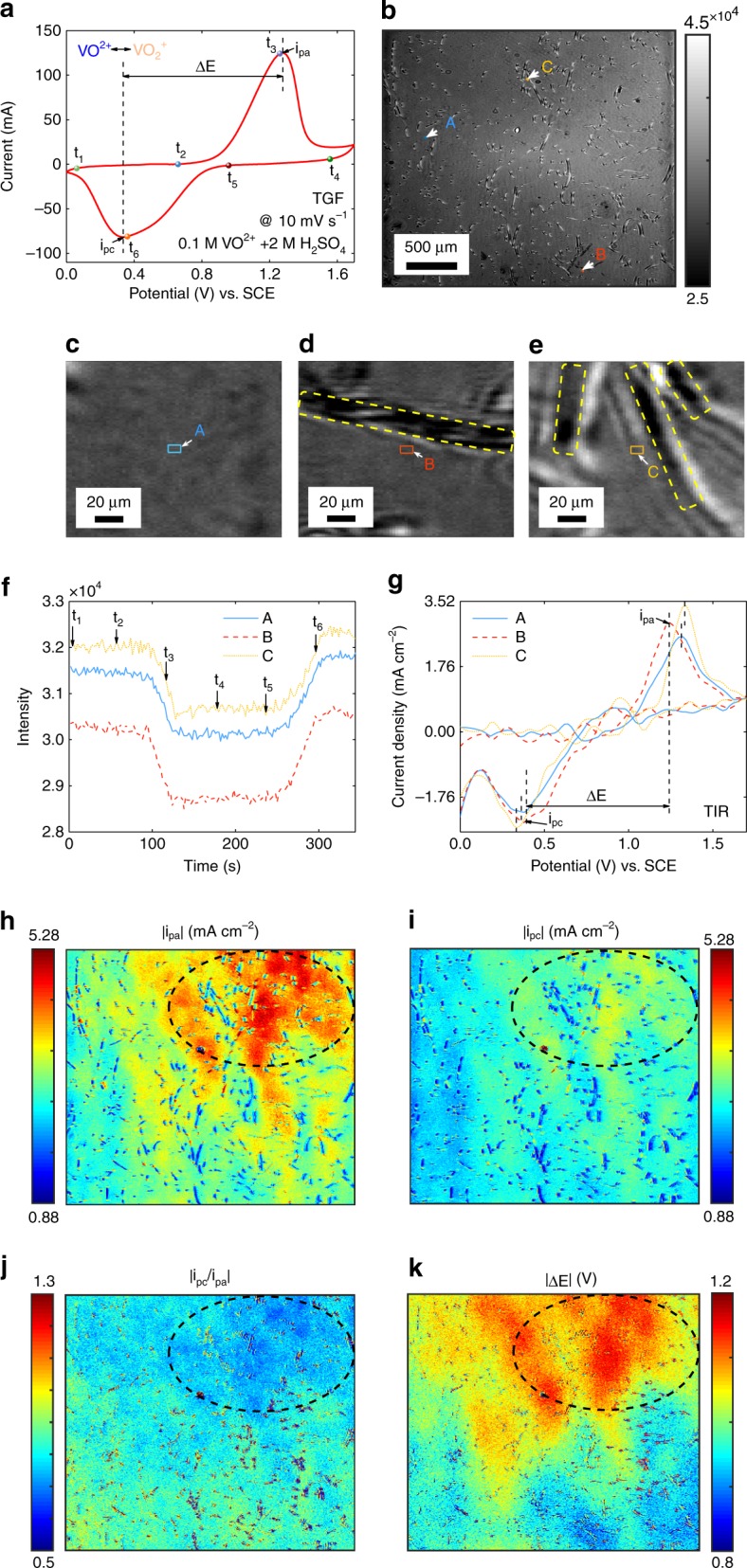
Flow charts of obtaining current density distribution by the captured images. **a** CV curve of a TGF in the positive electrolyte of 0.1 M VO^2+^ and 2 M H_2_SO_4_ at a scan rate of 10 mV s^−1^ recorded by EW. **b** Image captured by CCD at 0 V. **c**–**e** The enlarged views labeled as A, B and C in **b** respectively. Yellow dashed rectangle: carbon fiber contacted with the prism. **f** Time-varying averaged intensities of the rectangle area (5 × 5 pixels) labeled as A, B and C in **b** during the CV. **g** CV curves obtained by convolution calculation based on the data in **f**. **h**–**k** The mappings of the peak oxidation current densities (|*i*_pa_|), the peak reduction current densities (|*i*_pc_|), the ratio of two peaks (|*i*_pc_/*i*_pa_|) and the peak potential separation value (|Δ*E*|).

As the image intensity varies along with the RI of electrolyte on the electrode surface (Supplementary Fig. 3), it is feasible to map the current density distribution of electrode surface by the quantitative relationship between RI and current density. During a CV, electrochemical reaction of the positive electrolyte (0.1 M VO^2+^ and 2 M H_2_SO_4_) takes place on the TGF electrode by a potential sweep between 0 V and 1.7 V at a scan rate of 10 mV s^−1^. The CV curve detected by the electrochemical workstation (EW) and its peak oxidation current densities i_pa_, the peak reduction current densities i_pc_ and the peak potential separation value ΔE are displayed in Fig. 2a. When the potential gradually increases, VO^2+^ ions on the TGF are oxidized to VO_2_^+^ ions and the oxidation current raises. Inversely, VO_2_^+^ ions are reduced to VO^2+^ ions and the reduction current increases. The first TIR image is displayed in Fig. 2b, whose regions with visible optical contrast traces are multiple fibers in contact with the prism. The enlarged views of three regions in Fig. 2b reveal three target surroundings with no fiber (A), single fiber (B) and multiple fibers (C) within the range of 200 μm in diameter. The yellow rectangles in Fig. 2d, e are the fiber traces. The intensity images and the intensity variation images at t_1_–t_6_ in a cycle demonstrate the large intensity decrease and recovery (Supplementary Fig. 4). To quantify the time-varying intensity in a cycle, the averaged intensity points of A, B and C are shown in Fig. 2f. By deconvolution calculation (Supplementary Figs. 5–9), the current densities of A, B and C in Fig. 2g reflect their activity (represented by the peak oxidation current densities |*i*_pa_| and the peak reduction current densities |i_pc_|) and reversibility (represented by the ratio of two peaks |*i*_pc_/*i*_pa_| and the peak potential separation value |ΔE|) differences. For example, *i*_pa_ and *i*_pc_ of target C whose surroundings with multiple fibers are the largest, illustrating target C has the largest activity compared with A and B. However, since the detection signal is contributed by the concentration variation of the electrolyte from the electrochemical reaction of the local graphite fibers within the local penetration depth of the evanescent field, and the concentration variation of the diffused electrolyte from the reaction of the graphite fibers within the diffusion layer, the reaction rate (standard rate constant) and the density of the fibers contribute to the local current density, which is verified by the numerical simulation of the CV process (Supplementary Figs. 10–14). Therefore, the current density cannot be determined simply by the fiber density on the interface. The similarity of the CV curves obtained by the EW in Fig. 2a and TIRi sensor in Fig. 2g reveals the capability of the TIRi sensor to map current density distribution of the electrode. The mappings of |*i*_pa_|, |*i*_pc_|, |*i*_pc_/*i*_pa_| and |ΔE| distribution are obtained in Fig. 2h–k, which demonstrate their differences. The larger |*i*_pa_| and |*i*_pc_| regions (labeled by the dashed oval in Fig. 2h, i) has higher activity, but the reversibility of the corresponding regions in Fig. 2j, k are worse in view of the |*i*_pc_/*i*_pa_| away from one and larger |ΔE|. The TIRi sensor enables the above four parameters to characterize activity and reversibility distribution of the electrode. Since the total reflection at the areas where the graphite fiber in direct contact with the prism is not satisfied, the method cannot give an accurate current density by the electrolyte concentration variations at these areas. The current density of the associated void areas immersed in the electrolyte is mainly from the surrounding graphite fibers, which can reflect the activity distribution of the surrounding graphite fibers (Supplementary Fig. 15). The TIRi sensor is also utilized to detect electrochemical reaction of the positive electrolyte with different concentrations of VO^2+^ and 2 M H_2_SO_4_ on the TGF whose consistent results verify its feasibility (Supplementary Fig. 16).

### Activity and reversibility distribution comparison

**Fig. 3:**
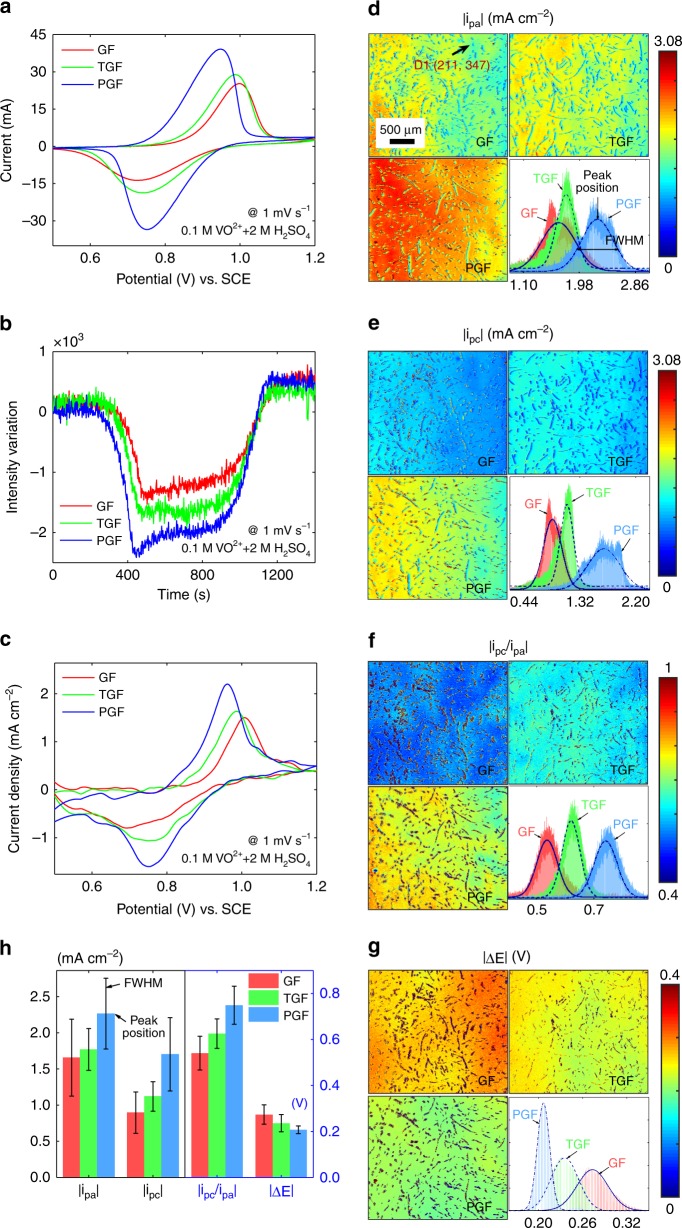
Activity and reversibility distribution comparison of three electrodes. **a** CV curves of the GF, TGF and PGF in the positive electrolyte of 0.1 M VO^2+^ and 2 M H_2_SO_4_ at a scan rate of 1 mV s^−1^ recorded by EW. **b** TIR intensity variations of the GF, TGF and PGF of point D1 (211, 347) recorded by the TIRi sensor. **c** CV curves by convolution calculation of the data in **b**. The mapping of the peak oxidation current densities |*i*_pa_| (**d**), the peak reduction current densities |*i*_pc_| (**e**), the ratio of two peaks |*i*_pc_/*i*_pa_| (**f**) and the peak potential separation value |Δ*E*| (**g**) of the GF, TGF, and PGF respectively and their distribution in the bottom right. **h** The peak position and FWHM (labeled in **d**) comparison of |*i*_pa_|, |*i*_pc_|, |*i*_pc_/*i*_pa_| and |Δ*E*| from the distributions in the bottom right of (**d**–**g**).

To further verify the feasibility of the TIRi sensor to determine activity and reversibility distribution, the CV curves of GF, TGF, and PGF are compared. A cyclic sweep potential is performed to induce redox reaction of vanadium ions on the GF, TGF and PGF respectively at a scan rate of 1 mV s^−1^. Their CV curves recorded by EW in Fig. 3a indicate that the activity and reversibility of the PGF is the best for its highest |*i*_pa_| and smallest |Δ*E*| while that of the GF is the worst for its lowest |*i*_pa_| and largest |Δ*E*|. The performance of the TGF locates between GF and PGF. This attributes to functional groups on the TGF and high area to volume ratio for the porous structure of the PGF compared with the GF (Supplementary Fig. 2). The intensity variations of the single point D1 (211, 347) in the GF, TGF and PGF are recorded by the TIRi sensor in Fig. 3b, which firstly decrease sharply for the oxidation current peak, and then remain stable for about 550 s away from reaction potential, further recover to the almost initial intensity for the reduction current peak. By deconvolution of the data in Fig. 3b, the obtained CV curves in Fig. 3c are consistent with that recorded by EW in Fig. 3a. Besides, the intensity variations and CV curves from full-image and the segment (point D1) are compared with good consistency (Supplementary Fig. 17, 18). The |*i*_pa_| mappings of the GF, TGF, and PGF in Fig. 3d demonstrate distribution difference of local peak oxidation currents. To quantify the distribution difference, the counts of the |*i*_pa_| distribution are compared in the bottom right of Fig. 3d for the GF, TGF, and PGF. The peak position of Gaussian fitting curve for |*i*_pa_| distribution of the PGF is the largest while the full width at half maximum (FWHM) of the TGF is the smallest, which imply that the PGF has the highest activity and the distribution difference of the TGF is the smallest. The |*i*_pc_| mappings and the distribution are similar to |*i*_pa_| distribution. The only difference is that the |*i*_pa_| of the corresponding region is larger than the |*i*_pc_|, which illustrates this reaction is not completely reversible. The region with higher |*i*_pa_| could provide larger |*i*_pc_| compared with other regions in Fig. 3d, e. The |*i*_pc_/*i*_pa_| and |Δ*E*| mappings and distributions in Fig. 3f, g manifest that the PGF has the best reversibility for its |*i*_pc_/*i*_pa_| closest to one and smallest |ΔE|. In detail, the quantitative peak position and FWHM of |*i*_pa_|, |*i*_pc_|, |*i*_pc_/*i*_pa_| and |ΔE| are compared for the GF, TGF, and PGF in Fig. 3h. It indicates that the TIRi sensor can not only quantify the activity and reversibility of the electrode, but also measure their distribution differences. However, it can only reflect the electrochemical activity distribution of the thin surface layer of graphite fibers (Supplementary Fig. 19). Furthermore, parallel measurements under different view of fields and parallel measurements from the same batches of graphite felts are executed, whose relatively consistent results verify that the method is capable of distinguishing the activity and reversibility differences (Supplementary Figs. 20–26).

### Bubble kinetics of oxygen evolution reaction

**Fig. 4:**
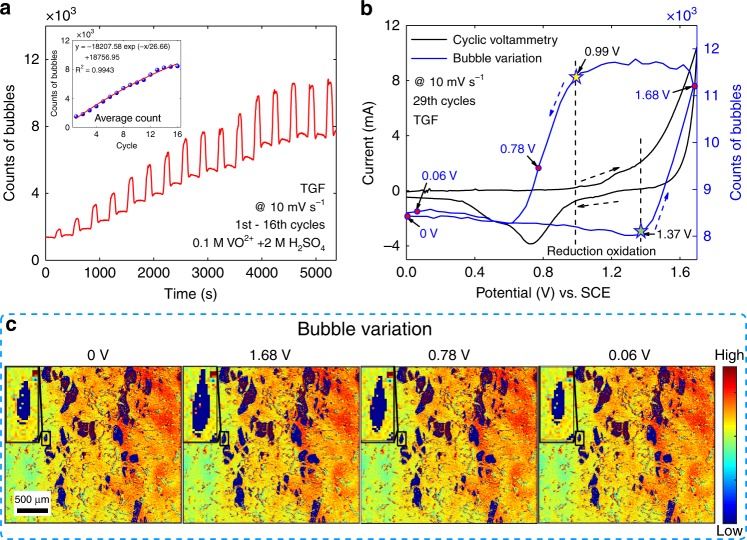
Bubble kinetics of oxygen evolution reaction in long-term cyclic voltammetry (CV). **a** The counts of bubbles in the 1st to 16th cycles during long-term CV of the TGF in the positive electrolyte of 0.1 M VO^2+^ and 2 M H_2_SO_4_ at a scan rate of 10 mV s^−1^ recorded by the TIRi sensor. Inset, the average counts of bubbles in each cycle and the fitting curve. **b** CV curve of the TGF in the positive electrolyte of 2 M H_2_SO_4_ and the counts of bubbles via the sweep potential in the 29th cycle. Black and blue dashed arrows indicate the potential sweep direction. Dashed lines point out the onset oxygen generation (green star) potential and consumption (yellow star) potential. **c** Bubble variation in combination with the mapping of the peak oxidation current densities |i_pa_| at the potential of 0, 1.68, 0.78, and 0.06 V during the 29th cycle.

To demonstrate the unique imaging advantage of the TIRi sensor, the process of OER from electrolysis of water in long-term CV is evaluated. A long-term CV is conducted on the TGF with positive electrolyte (0.1 M VO^2+^ and 2 M H_2_SO_4_) between 0 V and 1.7 V at a scan rate of 10 mV s^−1^. By calculating the counts of points (5 × 5 pixels as one point) covered by bubbles in contact with the surface, the counts of bubbles label the effective oxygen output from electrolysis of water. The bubble generation in the narrow potential window is more moderate, which is convenient for statistical calculation of bubbles (Supplementary Figs. 27, 28, Supplementary Video 1). As the recorded bubbles are from bubbles generated on the outer surface of the graphite felt and also from bubbles inside the graphite felt that diffuse to the prism, Fig. 4a shows the time-varying counts of bubbles in the 1st–16th cycles, which gradually increase accompanied by periodic fluctuations and finally reach a stable equilibrium state. The averaged counts of bubbles in each cycle are plotted in the inset, and the fitting function is ${y} = - 18207.58\exp \left( { - \frac{x}{{26.66}}} \right) + 18756.95$ with *R*^2^ = 0.9943, where *y* is counts of bubbles, *x* is cycle number. Besides, three types of the local bubble dynamics are compared to confirm the bubble distribution (Supplementary Fig. 29). To study the periodic fluctuations of the bubbles, oxygen evolution/reduction reaction of 2 M H_2_SO_4_ electrolyte on the TGF is drove at the same condition. In Fig. 4b, the CV curve (black curve) demonstrates the onset oxidation (1.37 V) and reduction (0.99 V) potentials exist (black dashed arrows: potential sweep direction) in a cycle. The counts of bubbles (blue curve) in the 29th cycle decrease very slowly, then start to increase rapidly at the onset oxygen generation potential (labeled as a green star) $\left( {2H_2O \to 4H^ + + O_2 + 4e^ - } \right)$^46^ and maintain stable from 1.7 V to the onset oxygen consumption potential (labeled as a yellow star), finally decrease sharply to the initial state $\left( {O_2 + 2H^ + + 2e^ - \to H_2O_2} \right)$^47^ and slightly increase (blue dashed arrows: bubble variation direction). Coincidentally, the onset oxygen generation/consumption potential is consistent with the onset oxidation/reduction potential, which indicates that the TIRi sensor has potential to determine the onset potential of the OER by mapping the bubble kinetics directly rather than CV/polarization curves. Furthermore, the kinetic curves of the counts of bubbles and the obtained onset potentials are relatively consistent at different cycles to further verify the feasibility of visually determining the onset potential of the oxygen evolution/reduction reaction by the counts of bubbles (Supplementary Fig. 30). Meanwhile, this method provides another unique advantage of mapping the onset oxidation potential distribution by measuring the local bubble kinetics of different regions, while CV/polarization curve can only present the whole onset oxidation potential. By combining the |*i*_pa_| mapping of the first cycle and the bubble covered regions of the average intensity image in the 29th cycle (blue areas in Fig. 4c), the combined images of bubble variation at 0, 1.68, 0.78, and 0.06 V (red points in Fig. 4b) are displayed in Fig. 4c. The insets (the enlarged view of the marked rectangle) clearly demonstrate distinct bubble size variation in one cycle. The video of the entire electrochemical reaction and OER process is attached in Supplementary Information as Supplementary Video 2, which shows that the oxygen evolution reaction begins at the 1st cycle and the region R with high activity is more likely to generate bubbles (Supplementary Fig. 31).

### Activity distribution of TGF in long-term CV

**Fig. 5:**
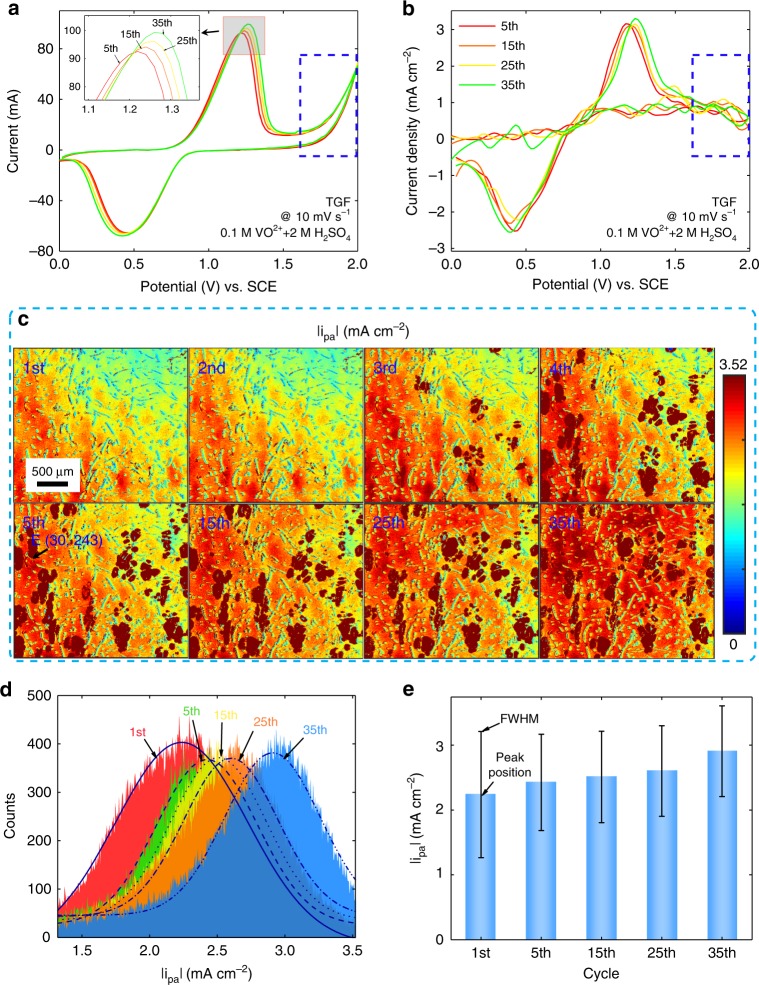
Activity distribution of thermal-activated graphite felt (TGF) in long-term cyclic voltammetry (CV). **a** Long-term CV curves (5th, 15th, 25th, 35th cycle) of the TGF in the positive electrolyte of 0.1 M VO^2+^ and 2 M H_2_SO_4_ at a scan rate of 10 mV s^−1^ recorded by EW. **b** Long-term CV curves of point E (30, 243) by convolution calculation of intensity variations recorded by the TIRi sensor. The mappings of the peak oxidation current densities |*i*_pa_| (**c**), |*i*_pa_| distribution (**d**) and the peak position and FWHM comparison of |*i*_pa_| (**e**) for the TGF in different cycles.

For further studying the vanadium ion redox reaction and oxygen evolution reaction on the electrode, the potential window between 0 and 2.0 V is applied to enable a long-term CV on the TGF with the positive electrolyte (0.1 M VO^2+^ and 2 M H_2_SO_4_) at a scan rate of 10 mV s^−1^. The CV curves of the 5th, 15th, 25th, and 35th cycles by EW in Fig. 5a reveal relative consistency, but their |*i*_pa_|, |*i*_pc_| and |Δ*E*| gradually increase, which imply the increasing activity and deteriorating reversibility. However, bubble generation would block the local electrochemical reactions and reduce the reaction area, so the peak oxidation/reduction currents of the CV curves decrease in the first few cycles, and then increase along with the increasing electrochemical activation effect (Supplementary Fig. 32). The oxygen evolution reaction in a large potential window is required to generate a large number of oxygen bubbles, resulting in a higher degree of electrode activation (Supplementary Figs. 27, 28, Supplementary Video 3). The corresponding CV curves of a single point E (30, 243) by the TIRi sensor are presented in Fig. 5b, in which the redox curves of vanadium ions are similar with that recorded by EW in Fig. 5a. Since the selected point E is not covered by bubbles, its CV curve does not display the OER (blue dash rectangle) in Fig. 5b. The |*i*_pa_| mappings are successively displayed in Fig. 5c in which dark brown areas are the regions covered by bubbles. The bubbles grow rapidly in the first five cycles (Supplementary Fig. 33), followed by an equilibrium state and then maintain stable. Meanwhile, although |*i*_pa_| is diverse at different regions in the same cycle, a gradual increase of the |*i*_pa_| at the same region emerges, which indicates the increasing activity owing to the generated oxygen-containing functional groups on the electrode^31,48^. The |*i*_pc_|, |*i*_pc_/*i*_pa_| and |Δ*E*| mappings of the TGF at different cycles are also compared (Supplementary Fig. 34). The quantitative result in Fig. 5d shows the |*i*_pa_| gradually increases. The peak positions and FWHMs of the Gaussian fitting curves in Fig. 5d demonstrate the increasing peak position and decreasing FWHM in Fig. 5e which indicate that the activity of the TGF is rising and its distribution becomes more homogeneous attributing to the growing oxygen-containing functional group on the electrode.

### Relationship between activity and bubble distribution

**Fig. 6:**
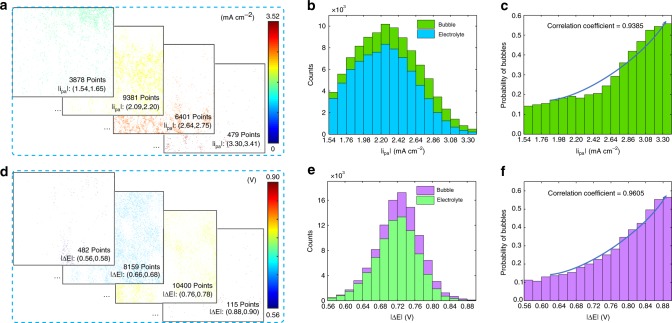
Activity distribution and bubble distribution in long-term cyclic voltammetry. The |*i*_pa_| (**a**) and |Δ*E*| (**d**) mappings of different intervals excluding the fiber contact regions at the 1st cycle. The count distribution of regions covered by electrolyte and by bubbles in 17 |*i*_pa_| (**b**) and |Δ*E*| (**e**) intervals respectively. The probability of bubble generation in different |*i*_pa_| (**c**) and |Δ*E*| (**f**) intervals.

In situ monitoring of the local current density distribution and the bubble generation of OER by the TIRi sensor enables to determine the relationship between the activity distribution of the electrode and OER distribution. In detail, as the refractive index of the bubble is far from that of the electrolyte, the reflected light from the region occupied by the bubble is very strong (Supplementary Fig. 9) and out of the almost linear range (R_L_ in Supplementary Fig. 3). So the local current density cannot be calculated correctly by deconvolution of this reflected light intensity. To avoid it, the |*i*_pa_|, |*i*_pc_|, |*i*_pc_/*i*_pa_| and |Δ*E*| of the 1st cycle without bubble are utilized to assess the activity and reversibility of the electrode. After excluding fiber contact regions with low reflected intensity for its absence from the high sensitivity range (Supplementary Fig. 35), the ranges of |*i*_pa_| and |Δ*E*| are divided into 17 intervals, respectively. The |*i*_pa_| and |Δ*E*| mappings locating in each interval are shown in Fig. 6a, d respectively (Supplementary Figs. 36, 39). For example, there are 3878 points locating in the |*i*_pa_| interval of 1.54–1.65 mA cm^−2^ in Fig. 6a. The distinguishing location distributions of |*i*_pa_| and |Δ*E*| in different intervals attribute to the diverse activity and reversibility distributions in different locations. After recording the counts of points covered by bubbles and electrolyte in each |*i*_pa_| and |Δ*E*| interval of the 35th cycle respectively, the distribution of |*i*_pa_| is plotted in Fig. 6b while that of |Δ*E*| is in Fig. 6e. The peak position of |*i*_pa_| is about 2.31 mA cm^−2^ and that of |ΔE| is 0.72 V. By calculation, the probability of bubbles ($P_{bubble} = \frac{{C_{bubble}}}{{C_{bubble} + C_{Electrolyte}}}$, *C*_bubble_ is counts of regions covered by bubbles, *C*_Electrolyte_ is counts of regions covered by electrolyte) generated in each |*i*_pa_| interval is displayed in Fig. 6c and that of |ΔE| in Fig. 6f, which indicate that bubbles are more likely to generate on the region with higher |*i*_pa_| (correlation coefficient: 0.9385) and larger |Δ*E*| (correlation coefficient: 0.9605). The probability of bubbles versus |*i*_pc_| and |*i*_pc_/|*i*_pa_| are also studied but the correlation is not significant (Supplementary Figs. 37, 38, 40). It means that the OER has larger probability to take place on the electrode with high activity and irreversibility (Supplementary Fig. 41). The high irreversibility renders the overpotential and then side reaction (OER, etc.) occurs^27^. By adjusting the scan rate to investigate the redox reaction of vanadium ions and the OER kinetics, the peak oxidation/reduction current of vanadium ions raises dramatically along with the increasing scan rate, however the onset potential of the OER is less affected by the scan rate. Besides, it is faster to generate bubbles and reach the equilibrium state at lower scan rate (Supplementary Figs. 42, 43). This attributes to the enough current density of the OER and its longer duration time at lower scan rate. Hence, the TIRi sensor provides an opportunity to detect the activity distribution and bubble distribution at the same time, and further to determine their relationship.

## Discussion

We have proposed a total internal reflection imaging (TIRi) sensor for mapping the local electrochemical reactions on graphite felt of the VFB during CV by detecting the local current density distribution, enabling imaging the activity and reversibility distributions with the spatial resolution of a single fiber. Without background current interference compared with SPRi sensors^41^ (Supplementary Fig. 44), the TIRi sensor is capable of imaging the redox reaction of vanadium ions and the OER by measuring the CV curves and recording the bubble location. The local CV curves recorded by the TIRi sensor are consistent with the whole CV curve recorded by EM, but the TIRi sensor can record the CV curves at different positions of the electrode simultaneously. By extracting the four parameters of the different CV curves, the mappings of |*i*_pa_|, |*i*_pc_|, |*i*_pc_/*i*_pa_| and |Δ*E*| are compared to determine the activity and reversibility distributions for three graphite felts with different activity, which verify the feasibility of the sensor. In long-term CV, the activity distribution and homogeneity of the electrode are enhanced by introducing the oxygen-containing functional group from the OER, whose generated bubbles periodically fluctuate and gradually increase to a stable equilibrium state. It provides a tool to measure the onset oxygen evolution potential, which is verified by the CV curve. It is revealed that higher activity and irreversibility play essential roles on bubble generation of the OER. Compared with existing techniques for directly imaging the bubble location^31^ and measuring the whole activity of the electrode in separation^22^, the present work combines the activity distribution detection with the bubble distribution imaging to determine the factors affecting the OER. This simple TIRi sensor has potential to in situ detect electrochemical reaction in flow batteries, water splitting, electrocatalysis, electrochemical corrosion and so on.

## Methods

### Materials

Equilateral prisms (Chinese ZF6 glass, length: 27 mm, thickness: 20 mm) were ordered from Fuzhou Alpha Optics Co., Ltd. (Fuzhou, China). Glucose was ordered from Aladdin Industrial Corporation (Shanghai, China). Vanadyl sulfate (VOSO_4_·3.5H_2_O, 99% purity) was ordered from Shenyang Haizhongtian Fine Chemical Co., Ltd. (Shenyang, China). Sulfuric acid (H_2_SO_4_) was purchased from Dongguan Dongjiang Chemical Reagent Co., Ltd. (Dongguan, China). Ferric trichloride (FeCl_3_, 99% purity) and sodium nitrate (NaNO_3_, 99% purity) were ordered from Tianjin Damao Chemical Reagent Factory (Tianjin, China). Graphite felts (10 mm × 10 mm × 5.4 mm) were purchased from Gansu Haoshi Cabon Fiber Co., Ltd. (Gansu, China). All other chemical reagents were purchased from Shenzhen Tianxiang Huabo Co., Ltd. (Shenzhen, China).

### Preparation and characterizations of electrode materials

The preparation method of TGF and PGF was detailedly described in the reported work^49^. In brief, after copiously rinsed with deionized water (Milli-Q water, 18.2 MΩ cm), graphite felt (GF) was thermally treated in the muffle furnace at 420 °C for 10 h to become the TGF. The PGF was prepared as follows. Firstly, the TGF immersed in 0.15 M FeCl_3_ and 1 M NaNO_3_ solution conducted hydrothermal reaction at 95 °C for 6 h to grow FeOOH nanorods. Secondly, after washed with deionized water and dried at 60 °C, the TGF with Fe_3_O_4_ was acquired by annealing in N_2_ gas at 900 °C for 3 h. Thirdly, the obtained TGF with Fe_3_O_4_ was immersed in concentrated hydrochloric acid for 12 h and washed with deionized water until the solution was neutral. Finally, the PGF was obtained by further drying at 60 °C to remove water. Scanning electron microscopy (SEM, field emission scanning electron microscope at 5 kV, ZEISS SUPRA^®^55) was utilized to characterize the size and the morphology of the GF, TGF and PGF in order to distinguish the electrochemical activity of three electrodes combined with the TIRi sensor.

### TIRi system for in situ detection of electrodes

The home-built TIRi system was utilized to image the electrode and detect the kinetics of vanadium ions during the electrochemical process. It consists of three parts: optical system, three-electrode system and display system (Supplementary Fig. 45). Similar to our previous work^50^, in Fig. 1a, the incident light from a red light emitting diode (A, LED, LR W5AP, Osram, Germany, central wavelength of 632.8 nm, electric power of 5 W) equipped with a 25× objective lens (B, GCO-2104, Daheng Optics, China) was collimated by an achromatic lens (D, GCL-010652, Daheng Optics, China, focal length 50 mm) into parallel light after passing through a homemade 0.3 mm aperture (C), and then reformed by a bandpass filter (E, FL632.8-10, Thorlabs, USA, central wavelength 632.8 nm, bandwidth 10 nm) and a linear polarizer (F, GCL-050003, Daheng Optics, China, extinction ratio of 500:1) to the p polarized light for its higher sensitivity than s polarized light (Supplementary Fig. 46). It radiated the total reflection interface of the reservoir-coupled sensor module (G, RCS) at the most sensitive angle near the total reflection angle (*θ*_TIR_). By adjusting the imaging lens (H, GuangZhou ZhiSai Electronic Technology Co., LTD, China), the parallel reflected light was focused on a charge coupled device (I, CCD, Retiga R3, Qimaging, Canada, 1920 × 1460 pixels, 4.54 × 4.54 μm^2^ pixel size, thermoelectric cooled to −20 °C) to record a series of images of the graphite felt with the imaging area of 2.92 × 3.84 mm^2^. The expanded view of the three-electrode system in Fig. 1b shows that a prism (G2) was combined with a fluid reservoir (G5) by a prism holder (G1) and a reservoir holder (G6), in which a strut (G4) pushed the graphite felt (G3) (10 × 10 × 5.4 mm^3^, compression ratio: 8%) in close contact with the prism. The platinum electrode (G7, Pt017, Tianjin Aida Hengsheng Technology Development Co., LTD, China, ϕ 1 mm × 37 mm, purity: 99.95%) was inserted from the middle of the side and through the graphite felt with the preferable conductivity and corrosion resistance as the working electrode^51^ (Supplementary Fig. 47). A graphite rod (G8) and a saturated calomel electrode (G9, SCE) partially immersed in the electrolyte were the counter electrode and reference electrode. Drove by an electrochemical workstation (K, EW, PARSTST 2273), two computers (J, L, the display system) received images from the TIRi sensor and CV curves from the EW respectively.

### Cyclic voltammetry measurement of electrodes

CV measurement was performed and recorded by the EW and TIRi sensor. Prior to experiment, 9 mL electrolyte of 0.1 M VO^2+^ and 2 M H_2_SO_4_ was injected into the RCS and the graphite felt was infiltrated by vacuum pumping. Subsequently, the RCS was installed on the TIRi sensor before the incident light was adjusted and the imaging lens was modulated to achieve high resolution imaging. Finally, after setting the potential window (0.5 V–1.2 V, 0 V–1.7 V, 0 V–2.0 V), scan rate (1 mV s^−1^, 10 mV s^−1^), cycles, exposure time (10 ms) and collection interval (2 s), optical and electrochemical measurements were conducted. The optical devices (CCD) and electrode potential are synchronized by manually pressing the “Start” button of the EW and image acquisition button of the TIRi sensor almost simultaneously.

### Data processing

A series of TIRi images captured by the CCD camera were converted to a three-dimensional matrix by a Matlab program. Each intensity point of the averaged image was acquired by smoothening over adjacent 5 × 5 pixels of the raw image as region of interest (ROI) (Supplementary Fig. 17). Considering spatial scale difference of the captured image and the incident angle of 51.5°, one pixel of the image in the vertical direction represents 2 μm of the object while that in the horizontal direction represents 3.21 μm of the object. So the actual size of the object is determined by counting the number of pixels or points. During a CV, the initial averaged image was subtracted from all the subsequent averaged images to become the intensity variation images. Each point of the intensity variation image was converted into a CV curve by deconvolution calculation (Supplementary Figs. 5–9). By extracting the peak oxidation/reduction current density and their corresponding potentials, the mapping of the peak oxidation current densities (|*i*_pa_|), the peak reduction current densities (|*i*_pc_|), the ratio of two peaks (|*i*_pc_/*i*_pa_|) and the peak potential separation value (|Δ*E*|) were obtained.

## Supplementary information

Supplementary Information

Supplementary Video 1

Supplementary Video 2

Supplementary Video 3

Supplementary Video 4

Supplementary Video 5

Supplementary Video 6

Supplementary Video 7

Supplementary Video 8

Supplementary Video 9

Source Data

## Data Availability

Data supporting this manuscript are available from the corresponding authors upon reasonable request. The source data underlying Fig. 1e, f, Fig. 2a, b, f–h, Fig. 3, Fig. 4, Fig. 5 and Fig. 6 are provided as a Source Data file.

## Code availability

Code supporting this manuscript are available from the corresponding authors upon reasonable request.
