# Supplementary material for: In situ mapping of activity distribution and oxygen evolution reaction in vanadium flow batteries
Source: Nat Commun. 2019 Nov 21;10:5286. doi: 10.1038/s41467-019-13147-9 (PMC6872572; doi:10.1038/s41467-019-13147-9)
Supplement: Supplementary file 1 — Supplementary Information [file 41467_2019_13147_MOESM1_ESM.pdf]

Supplementary Information:

In situ mapping of activity distribution and oxygen evolution reaction in vanadium flow batteries

Kaijie Ma[†,‡,§], Yunong Zhang[†], Le Liu[†,*], Jingyu Xi[†], Xinping Qiu[#], Tian Guan[§], Yonghong He[‡,§,*]

[†] *Institute of Green Chemistry and Energy, Graduate School at Shenzhen, Tsinghua University, Shenzhen 518055, China*

[‡] *Department of Physics, Tsinghua University, Beijing 100084, China*

[§] *Shenzhen Key Laboratory for Minimal Invasive Medical Technologies, Institute of Optical imaging and Sensing, Graduate School at Shenzhen, Tsinghua University, Shenzhen 518055, China*

[#] *Key Lab of Organic Optoelectronics and Molecular Engineering, Department of Chemistry, Tsinghua University, Beijing 100084, China*

** corresponding authors:*

Le Liu, Tel: 86-755-26036111. Email: liu.le@sz.tsinghua.edu.cn,

Yonghong He, Tel: 86-755-26036873. Email: heyh@sz.tsinghua.edu.cn.

1. Comparison of the TIRi sensor and SPRi sensor after cyclic voltammetry.

As the module of the TIR sensor is the prism, it is still stable in the positive electrolyte of 0.1 M VO^{2+} and 2 M H_2SO_4 after cyclic voltammetry (CV) as shown in Supplementary Figure 1(a). However, after depositing 2 nm chromium and 50 nm gold (ZNXC Co., Ltd., Beijing, China) on the slice (Fuzhou Alpha Optics Co., Ltd., Fuzhou, China) by magnetron sputtering, which is in contact with the prism by refractive index matching liquid (Cargille, USA), the module of the SPR sensor is also utilized to detect the electrochemical reaction of 0.1 M VO^{2+} and 2 M H_2SO_4 as the positive electrolyte during CV. It is clearly seen in Supplementary Figure 1(b) that the gold film is easy to fall off in the strongly oxidizing and acidic operating environment of the VFB. Hence, the TIRi sensor is used in this work to map the current density distribution and bubble generation of the VFB's electrode.

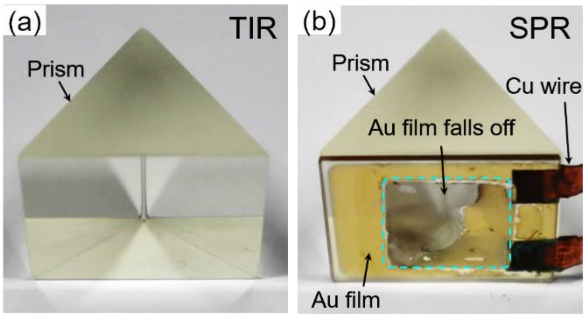

Supplementary Figure 1. Photograph of the (a) TIRi and (b) SPRi sensor module after cyclic voltammetry (CV) in the positive electrolyte of 0.1 M VO^{2+} and 2 M H_2SO_4.

2. Photograph and SEM comparison of GF, TGF and PGF electrodes.

Supplementary Figure 2(a) is the photograph of GF, TGF and PGF electrodes with each volume of 10 × 10 × 5.4 mm^3 used in this work. The SEM images of the GF and TGF are shown in Supplementary Figure 2(b1, b2) and Supplementary Figure 2(c1, c2) respectively, whose surfaces are smooth. The diameter of the fiber is about 13 μm. The SEM images of the PGF are shown in Supplementary Figure 2(d1, d2), whose surfaces are full of holes with higher area to volume ratio for the electrochemical reaction.

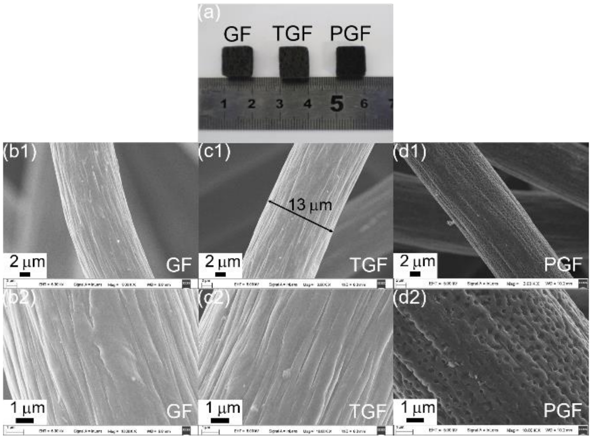

Supplementary Figure 2. (a) Photograph of GF, TGF and PGF electrodes. The SEM images of the GF (b1, b2), TGF (c1, c2) and PGF (d1, d2) electrodes.

3. Calibration of the TIRi sensor.

To calibrate the TIRi sensor system, a series of glucose solutions with different concentrations are injected into the fluid reservoir and in contact with the prism to enable the intensity response of the TIR sensor. The responses of 200 detections for three points (A, B and C in Supplementary Figure 3(c)) are plotted in Supplementary Figure 3(a) for each concentration of glucose solution, whose standard deviation is defined as the detection noise. It is found that the intensity variation is quasilinear in the concentration range of 125 - 200 g L^{-1}, which is marked as the almost linear range (R_L). According to the refractive indices of the glucose solutions with different concentrations detected by Abbe refractometer (2WAJ, Shanghai optical instrument five factory), the average intensities versus refractive indices are plotted in Supplementary Figure 3(b). The linear fitting lines of A, B and C in R_L are displayed in Supplementary Figure 3(b), whose slopes are the sensitivities

of A, B and C respectively. Repeat the same process and the absolute value mapping of the sensitivity is obtained as shown in Supplementary Figure 3(c). It can be seen that the regions with graphite felt fiber contact have smaller sensitivity owing to the intensity beyond the almost linear range. So these regions are not in the consideration ranges of the further activity and reversibility studies.

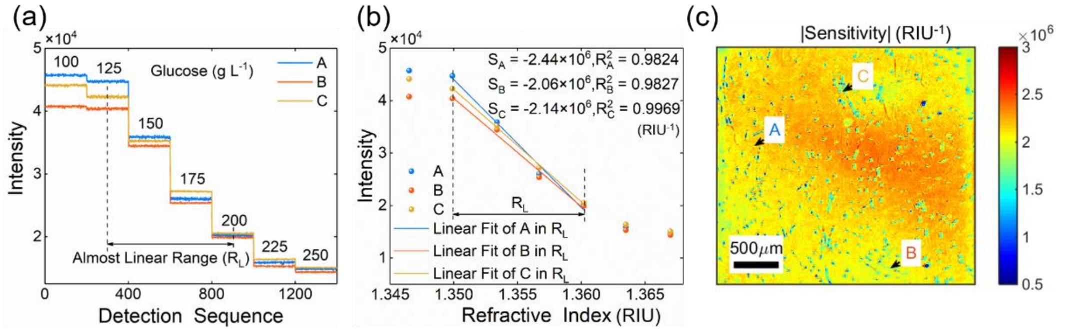

Supplementary Figure 3. (a) Step variations of the reflected light intensities corresponding to different concentrations of glucose solutions. Detection sequence is the number of detection. A, B and C are three points labelled in (c). The concentration range of glucose solutions between 125 g L^{-1} and 200 g L^{-1} is masked as the almost linear range. (b) The reflected light intensity shifts versus the refractive index and the fitting line in the almost linear range. (c) The absolute value mapping of the sensitivity of the imaging region.

4. Images of the intensity and intensity variation in one cycle.

Supplementary Figure 4(a) shows seven TIRi images captured by the CCD camera at different potentials in one cycle. Supplementary Figure 4(b) shows six images of intensity variation by subtracting the initial image at 0V from all the subsequent images in one cycle. In detail, the intensity of the image keeps stable between 0 V and 0.06 V for no electrochemical reactions. Subsequently, the intensity begins to decrease sharply at 0.66 V for the oxidation of the VO^{2+} ions leading to the increasing refractive index of the electrolyte. After maintaining stable between 0.66 V and 1.56 V, the intensity starts to recover into the almost initial intensity because of the reduction of the VO_2^+ ions. The visible intensity variation images confirm the TIRi sensor to detect the electrochemical reaction of the VFB’s electrode.

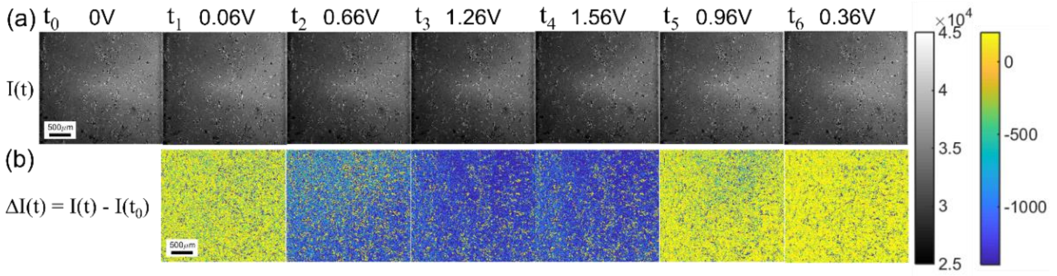

Supplementary Figure 4. (a) A series of intensity images I(t) captured by the CCD camera in one cycle. (b) The corresponding intensity variation images ΔI(t) in one cycle.

5. Calculation steps of the cyclic voltammetry curve.

Supplementary Figure 5(a) displays the time-varying intensities (I(t)) of A, B and C points. By subtracting the initial intensity at 0 V from all the subsequent intensities in one cycle ($\Delta I(t) = I(t) - I(t_0)$), the time-varying intensity variations ΔI(t) of A, B and C are shown in Supplementary Figure 5(b). As the fluctuation of the intensity variations in Supplementary Figure 5(b), the curves of the intensity variations are smoothed by the "smooth" function in MATLAB R2017a. The denoised results are plotted in Supplementary Figure 5(c). In this work, the electrochemical reaction does occur on the graphite felt, but the graphite felt is in close contact with the prism, so the detection signal is contributed by the concentration variation of the electrolyte within the local penetration depth of the evanescent field, and the concentration variation of the diffused electrolyte from the electrochemical reaction of the graphite fibers within the diffusion layer. Overall, since the size of the electrolytic cell (fluid reservoir, 27 mm × 27 mm × 24 mm) is much larger than the diffusion length (~ 1 mm), the semi-infinite diffusion model can be applied in this work. Considering a redox reaction in the cyclic voltammetry[1], the diffusion equation

$$\frac{\partial C_O(z,t)}{\partial t} = D_O \frac{\partial^2 C(z,t)}{\partial z^2} \tag{S1}$$

can be solved under the initial condition $C_O(z,0) = C_O^0$, the semi-infinite diffusion boundary condition $\lim_{z\to\infty} C_O(z,t) = C_O^0$ and flux balance condition $i(t) = nFD_O[\frac{\partial C_O(z,t)}{\partial z}]_{z=0}$. The concentrations of oxidized and reduced species are obtained as follow[2]:

$$C_O(0,t) = C_O^0 - [nF(\pi D_O)^{1/2}]^{-1} \int_0^t i(t')(t-t')^{-1/2} dt' \tag{S2}$$

$$C_R(0,t) = C_R^0 + [nF(\pi D_R)^{1/2}]^{-1} \int_0^t i(t')(t-t')^{-1/2} dt' \tag{S3}$$

where C_O^0 and C_R^0 are the concentrations of the oxidized and reduced species at the initial time, n

is the electron number of the redox reaction, F is the Faraday constant, D_O and D_R are the diffusion coefficients of the oxidized and reduced species, $i(t')$ is the current density. Similar to the reported work[2], the response of the TIR sensor in view of its detection depth is

$$I(t) = R[\alpha_O C_O(z,t)|_{z=0} + \alpha_R C_R(z,t)|_{z=0}] \tag{S4}$$

where $I(t)$ is the reflected light intensity, α_O and α_R are the refractive index changes per unit concentration of the oxidized and reduced species respectively. R is the intensity change per unit refractive index. According to equations (S2), (S3) and (S4), the intensity can be derived:

$$I(t) = I(t_0) + R(\alpha_R D_R^{-1/2} - \alpha_O D_O^{-1/2})(nF\pi^{1/2})^{-1} \int_0^t i(t')(t-t')^{-1/2}dt' \tag{S5}$$

where the initial intensity is $I(t_0) = R(\alpha_O C_O^0 + \alpha_R C_R^0)$. Define a parameter: b = $R(\alpha_R D_R^{-1/2} - \alpha_O D_O^{-1/2})$, the intensity variation is shown as follows:

$$\Delta I(t) = I(t) - I(t_0) = \frac{b}{nF}\pi^{-1/2} \int_0^t i(t')(t-t')^{-1/2}dt' \tag{S6}$$

Rewrite the equation (S6) as:

$$\frac{nF}{b}\Delta I(t) = \pi^{-1/2}\int_0^t i(t')(t-t')^{-1/2}dt' = \int_0^t i(t')[\pi(t-t')]^{-1/2}dt'$$
$$= convolution[i(t), (\pi t)^{-1/2}] \tag{S7}$$

Hence, the current density can be derived as:

$$i(t) = deconvolution[\frac{nF}{b}\Delta I(t), (\pi t)^{-1/2}] \tag{S8}$$

Actually, the value of "nF/b" is a constant c (nF/b = c). For simplicity, the relative current density distribution is calculated herein by supposing nF/b =1 in this work. We can get the current density distributions by setting nF/b with the different values, but the ratio of current density between different positions remains the same. By the following calibration experiment, the absolute current density distribution remains the same under the condition of nF/b with different values. The derived CV curves of A, B and C in Supplementary Figure 5(d) show the relative local current densities along with the potential.

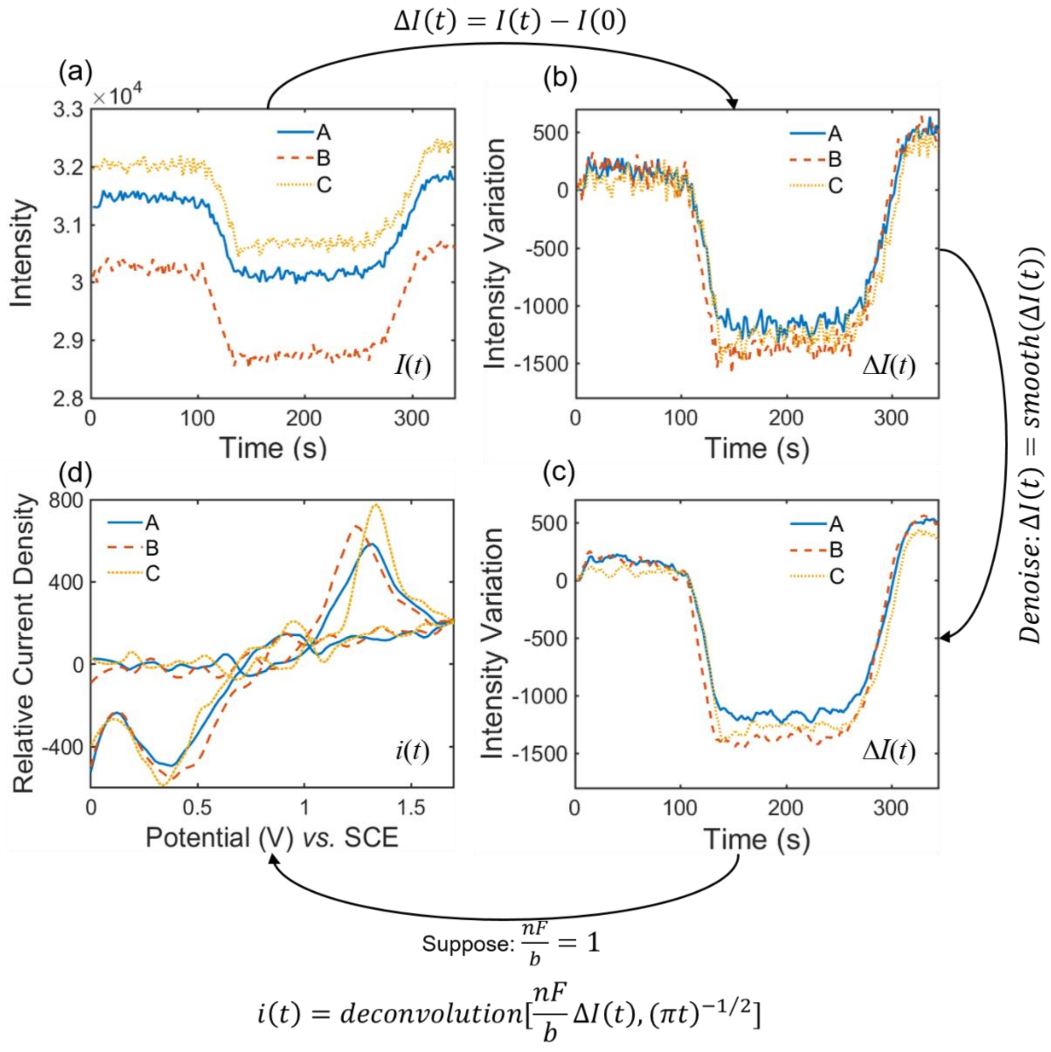

Supplementary Figure 5. (a) Time-varying averaged intensities of A, B and C during CV. (b) The intensity variations of A, B and C obtained by subtracting the initial intensity from time-varying intensities shown in (a). (c) Denoising result by smoothing the intensity variations of A, B and C in (b). (d) CV curves by convolution calculation of the intensity variations in (c). Note: suppose $\frac{nF}{b} = 1$, the calculated current density is the relative value.

In order to verify the current density measurements by deconvolution of the intensity variation, we adopt the calibration experiment to provide the current density with a physical unit. A graphite plate with smooth interface is used as the uniform and well-tunable electrode for calibrating the light intensity and the current density measurements. As shown in Supplementary Figure 6(a, d), one interface of the graphite plate (cross-sectional area: 10 × 12 mm^2) is in contact with the prism and the other interfaces are wrapped by tapes to avoid contact with the electrolyte. The graphite plate is in contact with the prism (Supplementary Figure 6(c)). As the two contact surfaces are not ideally smooth, there are gaps between the surfaces of the graphite plate and the prism, where the

electrolyte can be immersed. The electrochemical reaction current flows through only the interface in contact with the prism. Hence, dividing the current by the cross-sectional area, the current density can be obtained. Besides, compared with the above graphite plate with tape, a similar graphite plate without tape in Supplementary Figure 6(b, e) is also used as the electrode. The electrochemical reaction can occur at all contact interfaces of the graphite plate. As shown in Supplementary Figure 6(c), the calibration experiment at the potential window of 0.5 - 1.2 V is conducted on three-electrode system (the working electrode (WE): the graphite plate; the counter electrode (CE): a graphite rod; the reference electrode (RE): a saturated calomel electrode). In order to tune the current density, a series of scan rate (1 mV s^{-1}, 2 mV s^{-1}, 3 mV s^{-1}, 4 mV s^{-1}) is set in the calibration experiment.

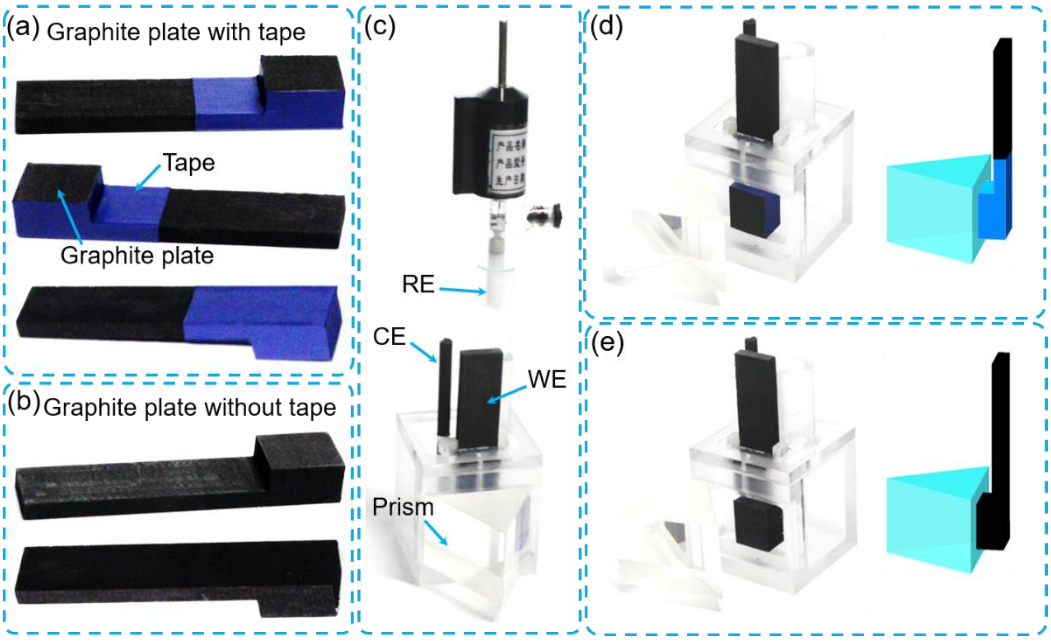

Supplementary Figure 6. A graphite plate with smooth interface used as the uniform and well-tunable electrode in the experiment. (a) Graphite plate with tape: adhesive tape is used to adhere the outer interfaces of the graphite plate except the interface (cross-sectional area: 10 × 12 mm^2) in contact with the prism. (b) Graphite plate without tape: the outer interfaces of the graphite plate are immersed with electrolyte. (c) Three-electrode system in the calibration experiment. The working electrode (WE): a graphite plate; the counter electrode (CE): a graphite rod; the reference electrode (RE): a saturated calomel electrode. (d) The graphite plate with tape is in contact with the prism. (e) The graphite plate without tape is in contact with the prism.

Since the electrode (graphite plate) is in contact with the prism and the other surface interfaces are wrapped by tape to avoid contact with the electrolyte, the concentration change of the redox

couple during the CV cannot be updated in time at the high scan rate, and the electrode activity cannot be fully exerted. As shown in Supplementary Figure 7, time-varying averaged TIR intensities of the full images during CV decrease firstly corresponding to oxidation process (VO^{2+} to VO_2^+) and then increase corresponding to reduction process (VO_2^+ to VO^{2+}). As the electrolyte in the gap between the graphite plate and the prism cannot be updated effectively and deviates from the original state, it can be seen that the intensity difference of point O and R is very small while that of point O' and R' is Δ. The reaction reversibility of the thermal activated graphite felt (TGF) is better than that of the graphite plate, owing to the porous structure of the TGF which is beneficial to the renewal of electrolyte. However, only one interface of the graphite plate with tape in contact with the prism serves as a reaction site and the electrolyte renewal is poor. In order to determine the calibration process, we adopt the linear sweep voltammetry (LSV) to reduce the electrolyte renewal difference by shortening the time instead of the CV. Furthermore, the different peak oxidation current densities of the LSV at different scan rates are obtained to achieve calibration.

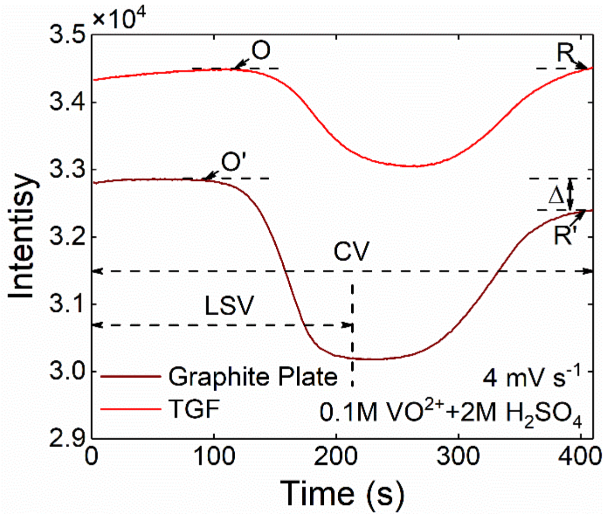

Supplementary Figure 7. Comparison of time-varying averaged TIR intensities of the full images of the graphite plate and TGF recorded by the TIRi sensor during CV.

As shown in Supplementary Figure 8(a), the LSV curves of the graphite plate with tape in the positive electrolyte of 0.1 M VO^{2+} and 2 M H_2SO_4 at different scan rates (1 mV s^{-1}, 2 mV s^{-1}, 3 mV s^{-1}, 4 mV s^{-1}) recorded by electrochemical workstation (EW) are obtained, whose peak oxidation currents (marked by arrows) increase along with the increasing scan rates. Besides, by convolution calculation of the averaged intensity variations of the full images recorded by the TIRi sensor, LSV curves are plotted in Supplementary Figure 8(b), whose peak oxidation currents demonstrate a

similar trend with those by EW in Supplementary Figure 8(a). It should be pointed out that the small current in the initial stage of LSV curve in Supplementary Figure 8(a) is from the non-Faraday current of the electrochemical workstation. This current does not appear in Supplementary Figure 8(b) because the TIRi sensor is to obtain the Faraday current from the redox reaction. Picking out the peak oxidation currents from the LSV curves in Supplementary Figure 8(a, b) and calculating their densities, the current densities ($i_{pa(EW)}$) by EW and the relative current densities ($i_{pa(TIR)}$) by the TIRi sensor are plotted in Supplementary Figure 8(c). They are linear with the square root of scan rates ($v^{1/2}$), proving the Randles-Sevcik equation. In Supplementary Figure 8(d), the mapping of the relative peak oxidation current density $|i_{pa}|$ demonstrates a relatively uniform distribution at each scan rate. The average value of $|i_{pa}|$ increases with the scan rate. The standard deviations (Std) of $|i_{pa}|$ are also labelled in Supplementary Figure 8(d) and used as the error bars in Supplementary Figure 8(c). LSV curves of the graphite plate with and without tape at the scan rates of 1 mV s^{-1} recorded by EW are compared in Supplementary Figure 8(e), whose i_{pa} of the graphite plate without tape is about 4.2581 times (10.2191 mA / 2.3999 mA = 4.2581) of that with tape. The whole electrolyte immersed interface of the graphite plate without tape is 518 mm^2 and the interface in contact with the prism is 120 mm^2. Their ratio is 4.3167, which is almost consistent with the ratio (4.2581) of i_{pa}. It can be confirmed that the electrochemical reaction takes place on the interface of the electrode and the electrochemical workstation collects the overall current of the electrode. According to the result of Supplementary Figure 8(e), the current densities in the manuscript should be obtained by dividing the currents by the surface area of the graphite felt with porous structure. Supplementary Figure 8(f) shows LSV curves of the graphite plate with and without tape at the scan rates of 1 mV s^{-1} by convolution calculation of the intensity variations of the full images recorded by the TIRi sensor. The relative peak oxidation current density $|i_{pa}|$ (461.3607) of the graphite plate with tape is similar with that (486.5564) without tape. Their difference (25.1957) is within the range of Std (47.7241). It indicates that the relative current densities of the graphite plate with (Supplementary Figure 8(d)) and without (Supplementary Figure 8(g)) tape are consistent by convolution calculation of the intensity variations recorded by the TIRi sensor, owing to only recording the response of the interface in contact with the prism. Hence, the current densities of the graphite plate with tape are obtained by dividing the EW currents by the cross-sectional area (10×12 mm^2), which should be

equal to the relative current densities obtained by the TIRi sensor. In order to verify it, the relationship between the current density ($i_{pa(EW)}$) recorded by EW and the relative current density ($i_{pa(TIR)}$) obtained by the TIRi sensor is shown in Supplementary Figure 9, and the quantitative equation is $i_{pa(EW)} = 0.0044 i_{pa(TIR)}$. So the relative current density obtained by the TIRi sensor can be converted to the current density (mA cm^{-2}).

In order to derive the current density according to the equation $i(t) = deconvolution[\frac{nF}{b}\Delta I(t), (\pi t)^{-1/2}]$, these parameters ($n$, F, b, π) and variables ($\Delta I(t)$, t) should be known. Among them, the parameters (n, F, π) are known. $\Delta I(t)$ can be obtained by the TIRi sensor and t can be recorded. The parameter (b) is the only unknown one, which is about the diffusion coefficients of the oxidized and reduced species (D_O, D_R) and the refractive index changes per unit concentration of the oxidized and reduced species (α_O, α_R). For the sake of simplicity, the diffusion coefficients are not obtained in this work. We calculate the relative current density by supposing nF/b =1 in Supplementary Figure 5. Hence, the calculated relative current density is not absolute value. Compared to measuring the diffusion coefficient, we obtain the current density with a physical unit through the calibration experiment. According to the above quantitative relationship between the current density ($i_{pa(EW)}$) recorded by EW and the relative current density ($i_{pa(TIR)}$) obtained by the TIRi sensor ($i_{pa(EW)} = 0.0044 i_{pa(TIR)}$), the current density with a physical unit recorded by the TIRi sensor can be achieved.

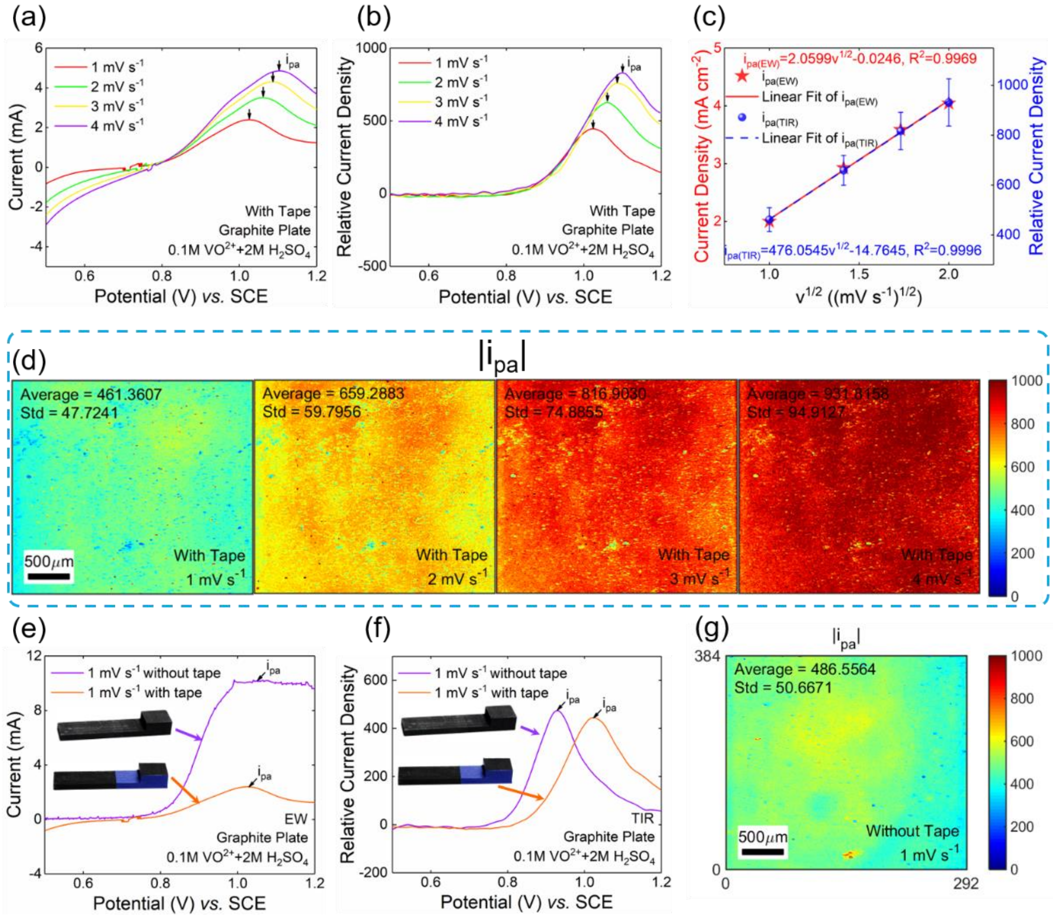

Supplementary Figure 8. (a) Linear sweep voltammetry (LSV) curves of the graphite plate with tape in the positive electrolyte of 0.1 M VO^{2+} and 2 M H_2SO_4 at different scan rates (1 mV s^{-1}, 2 mV s^{-1}, 3 mV s^{-1}, 4 mV s^{-1}) recorded by electrochemical workstation (EW). (b) LSV curves by convolution calculation of the intensity variations of the full images recorded by the TIRi sensor. (c) The mapping of the peak oxidation current densities $|i_{pa}|$ at different scan rates obtained from (a) and (b). Error bar: the standard deviations (Std) of $|i_{pa}|$ from (d). (d) The mapping of $|i_{pa}|$ at different scan rates and their average values and standard deviations. (e) LSV curves of the graphite plate with and without tape in the positive electrolyte of 0.1 M VO^{2+} and 2 M H_2SO_4 at the scan rates of 1 mV s^{-1} recorded by EW. (f) LSV curves of the graphite plate with and without tape at the scan rates of 1 mV s^{-1} by convolution calculation of the intensity variations of the full images recorded by the TIRi sensor. (g) The mapping of $|i_{pa}|$ at the scan rates of 1 mV s^{-1} and the average value and standard deviation

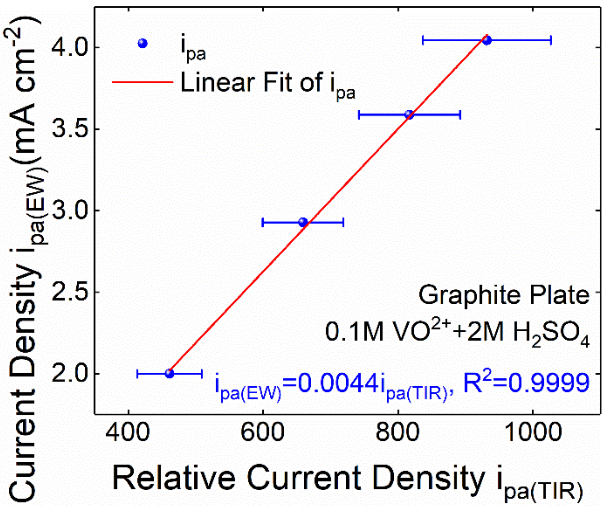

Supplementary Figure 9. The relationship between the current density ($i_{pa(EW)}$) recorded by EW and the relative current density ($i_{pa(TIR)}$) obtained by the TIRi sensor.

6. Simulation of the cyclic voltammetry process.

To prove the semi-infinite diffusion model and the convolution relationship in Supplementary Figure 5, numerical simulation is provided. We utilize the simulation software COMSOL Multiphysis 5.2 to simulate the CV process of VO^{2+} - VO_2^+. To simplify this process, the horizontal and vertical 2 - dimension (2D) distributions are studied here. The Bulter-Volmer equation is used to describe the reaction on the electrode as:

$$i = FAk^0[C_O(0,t)e^{-\alpha f(E-E^\theta)} - C_R(0,t)e^{-(1-\alpha)f(E-E^\theta)}]$$

where k^0 is the standard rate constant. E^θ is the standard electrode potential. α is transfer coefficient. $f = F/RT$. For reversible reaction, the Bulter-Volmer equation can be converted to the Nernst equation. In this work, α is 0.5. E^θ is 0.8 V. k^0 is 8.5×10^{-6} m s^{-1} for carbon electrode. E (potential window) is 0.5 V - 1.2 V (vs. SCE). The scan rate (v) is 0.001 V s^{-1}. The initial concentration $C_O(0,t)$ of VO^{2+} is 100 mol m^{-3}. The diffusion coefficient[3] of the oxidized species (D_O) or the reduced species (D_R) is 3.9×10^{-10} m^2 s^{-1}. The temperature (T) is 298.15 K.

As shown in Supplementary Figure 10(a, b), two kinds of electrodes are simulated to compare the concentration distribution of electrolyte. One is the graphite felt (thickness: 5.4 mm) in contact with the prism in Supplementary Figure 10(a). We assume that the graphite fibers (diameter: 0.013 mm) are distributed in arrays and the horizontal/vertical distance between graphite fibers is 0.163 mm according to the densities of graphite felt and graphite fibers. The other is the graphite film on the prism which is similar to the gold film on the prism as the SPR sensor in Supplementary Figure

10(b). The CV processes of these two electrodes are simulated and compared. The point Q1 (0, 12.063) in Supplementary Figure 10(a, b) is selected as region of interest at which the fiber is in contact with the prism. The time-varying concentrations of VO^{2+} at point Q1 in the CV of graphite felt and graphite film are demonstrated in Supplementary Figure 10(c), which is almost consistent. It illustrates that regardless of whether the electrode acts as a sensing film layer, the concentration variation of the reactants in the contact interface is very similar as long as the electrode is in direct contact with the prism, indicating that the current density distribution is similar. By convolution calculation of data in Figure R(c), the corresponding CV curves at point Q1 is obtained and show their similarity. It preliminarily verifies the feasibility of applying the convolution model to the calculation of the current density distribution of the graphite felt during CV.

Besides, the time-varying concentration distributions of VO^{2+} in the CV of graphite felt and graphite film are shown in Supplementary Figure 10(e, f), Supplementary Video 4 and Supplementary Video 5, which are decrease at first and recover later on the electrode. The concentration variation of VO^{2+} occurs locally in the vicinity of the electrode and most of the electrolyte concentration remaining in the electrolytic cell is constant. The enlarged view of the region masked by the purple rectangle is located at the bottom right corner, which shows that the diffusion length (1 mm) is much smaller than the length (27 mm) of the electrolytic cell. Hence, it verifies the feasibility of the semi-infinite diffusion model.

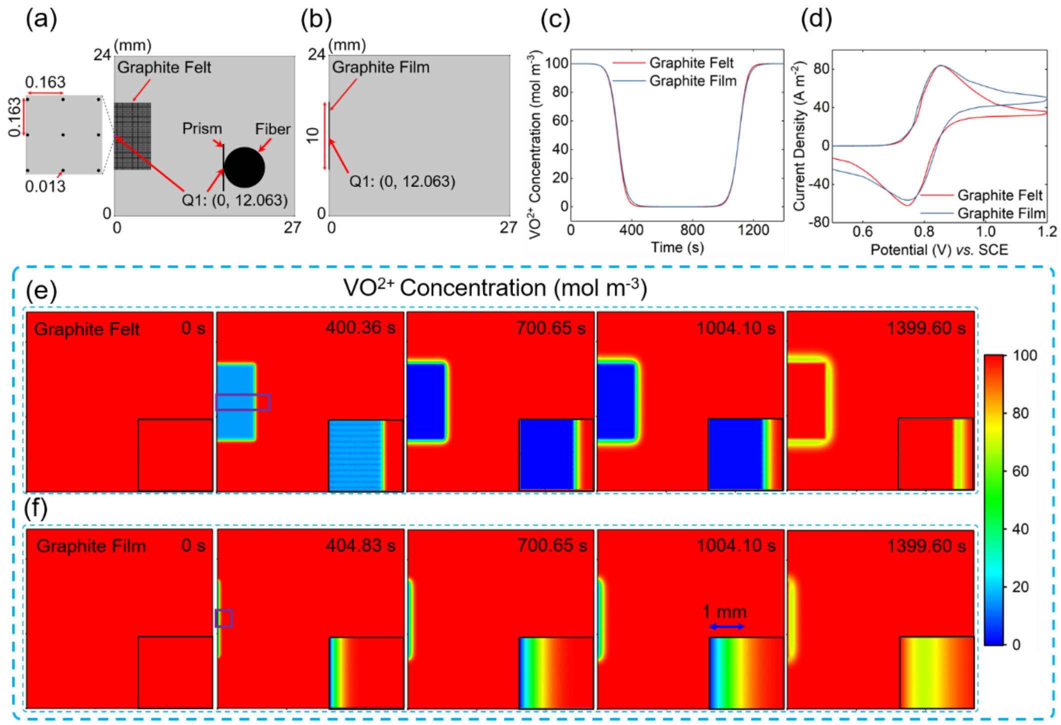

Supplementary Figure 10. (a) Vertical schematic of the graphite felt in the fluid reservoir. Horizontal and vertical distances between graphite fibers: 0.163 mm. The diameter of the fiber: 0.013 mm. (b) Vertical schematic of the graphite film (similar to gold film) in the fluid reservoir. (c) The time-varying concentrations of VO^{2+} at point Q1 in the cyclic voltammetry (CV) of graphite felt and graphite film. (d) The corresponding CV curves at point Q1 by convolution calculation of data in (c). The time-varying concentration distributions of VO^{2+} in the CV of (e) graphite felt and (f) graphite film. Figure at the bottom right corner: the enlarged view of the region masked by the purple rectangle.

We compare the time-varying concentrations and the corresponding CV curves at different points (fiber contact point and the void region) of the contact interface and at the local/overall region. As shown in Supplementary Figure 11(a, b), the time-varying concentrations of VO^{2+} and the corresponding CV curves at point Q1 (fiber contact point), Q2 and Q3 (the void region) in the CV of graphite felt are different. Meanwhile, the CV curve at point Q1 (fiber contact point) has larger peak current density and smaller peak potential separation value than those (Q2, Q3) at the void region, which illustrates the reaction occurs on the fibers and the species diffuse towards the void region. To compare the situation of the local and overall difference, the time-varying concentrations of VO^{2+} and the corresponding CV curves at point Q1 (fiber contact point) and region M (the average of the whole graphite felt) are also different in Supplementary Figure 11(c, d) because the

average value of the whole graphite felt is from the fiber contact points and the void regions.

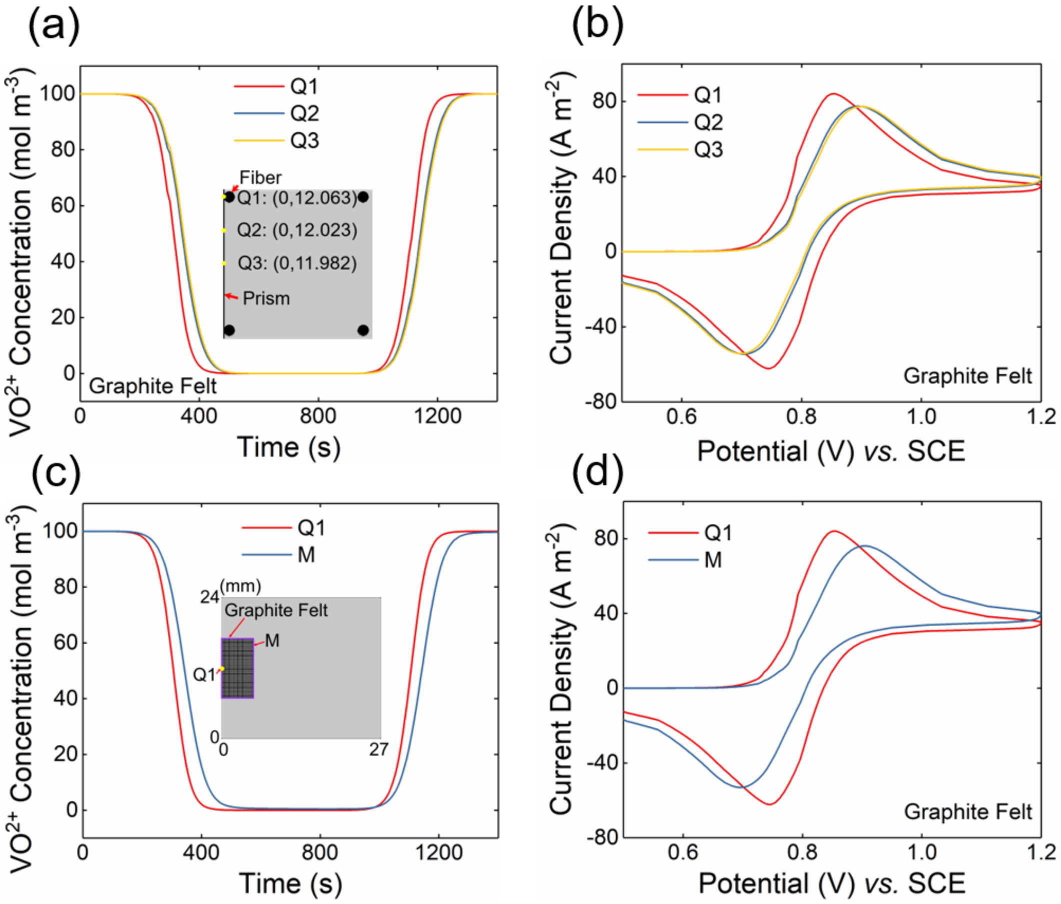

Supplementary Figure 11. (a) The time-varying concentrations of VO^{2+} at point Q1, Q2 and Q3 (labelled as yellow points) in the cyclic voltammetry (CV) of graphite felt. (b) The corresponding CV curves at point Q1, Q2 and Q3 by convolution calculation of data in (a). (c) The time-varying concentrations of VO^{2+} at point Q1 (yellow point) and region M (the average of the whole graphite felt) in the CV of graphite felt. (d) The corresponding CV curves at point Q1 and region M by convolution calculation of data in (c).

For further studying the contributions of the detection signal, we have studied the effects of diffusion effect and different electrode reaction rates on concentration variation of the electrolyte, which are described in the following three aspects.

(1). The diffusion effect between fiber arrays on concentration variation in vertical direction.

As shown in Supplementary Figure 12(a, b), the graphite fibers with 1 array and 2 arrays are compared to consider the diffusion effect between fiber arrays on the concentration distribution of electrolyte. In Supplementary Figure 12(a), the graphite fibers with 1 array are in contact with prism. In Supplementary Figure 12(b), one array is in contact with prism and the other is 1 mm (diffusion

length) from the first array. It can be seen that the time-varying concentrations of VO^{2+} at point Q1, Q2 and Q3 in the CV of graphite fibers with 1 array in Supplementary Figure 12(c) and the corresponding CV curves in Supplementary Figure 12(d) are slightly different from those with 2 arrays respectively. It attributes to the fact that the electrolyte which undergoes the redox reaction on the second array diffuses to the first array, affecting its concentration distribution. The enlarged views (the region masked by the purple rectangle at the bottom right corner) of the time-varying VO^{2+} concentration distributions in the CV of graphite fibers with 1 array in Supplementary Figure 12(e), Supplementary Video 6 and graphite fibers with 2 arrays in Supplementary Figure 12(f) and Supplementary Video 7 clearly demonstrate the diffusion effect between fiber arrays on concentration variation.

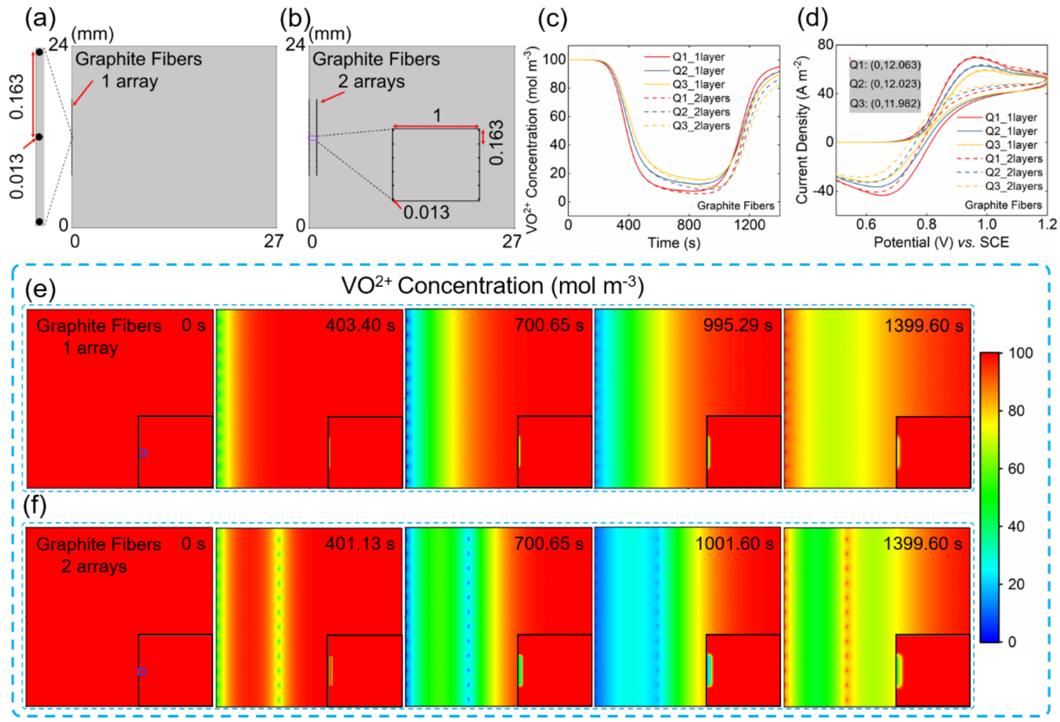

Supplementary Figure 12. (a) Vertical schematic of the graphite fibers with 1 array in the fluid reservoir. The distance between graphite fibers: 0.163 mm. The diameter of the fiber: 0.013 mm. (b) Vertical schematic of the graphite fibers with 2 arrays in the fluid reservoir. The distance between two arrays: 1 mm. (c) The time-varying concentrations of VO^{2+} at point Q1, Q2 and Q3 in the cyclic voltammetry (CV) of graphite fibers with1 array and graphite fibers with 2 arrays. (d) The corresponding CV curves at point Q1, Q2 and Q3 by convolution calculation of data in (c). The enlarged views (the region masked by the purple rectangle at the bottom right corner) of the time-varying VO^{2+} concentration distributions in the CV of (e) graphite fibers with 1 array and (f) graphite fibers with 2 arrays.

(2). The diffusion effect between fiber distribution on concentration variation in horizontal direction.

As shown in Supplementary Figure 13(a), the point F1 at the region with a tight distribution of fibers and the point F2 at the region with a sparse distribution of fibers are selected as region of interest to consider the diffusion effect between fiber distribution on the concentration distribution of electrolyte. It can be seen that the time-varying concentrations of VO^{2+} at point F1 and F2 in Supplementary Figure 13(b) are slightly different and the corresponding CV curve at point F1 has larger peak current than that at point F2 in Supplementary Figure 13(c), which attributes to the point F1 at the region with a tight distribution of fibers. The time-varying VO^{2+} concentration distributions in the CV of graphite fibers in Supplementary Figure 13(d) and Supplementary Video 8 clearly demonstrate the diffusion effect between fibers on concentration variation.

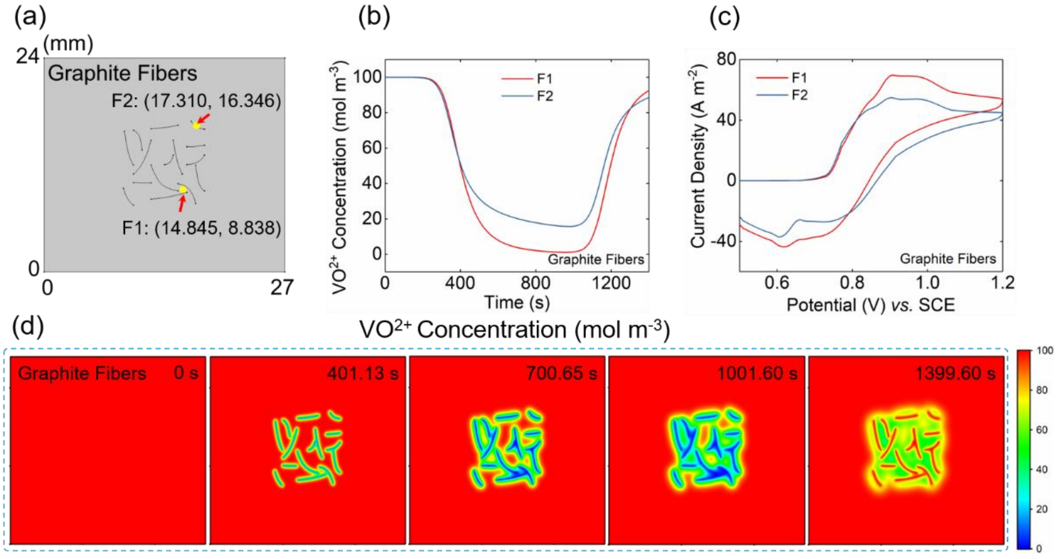

Supplementary Figure 13. (a) Horizontal Schematic of the graphite fibers (random distribution) in the fluid reservoir. F1: the point at the region with a tight distribution of fibers. F2: the point at the region with a sparse distribution of fibers. (b) The time-varying concentrations of VO^{2+} at point F1 and F2 in the cyclic voltammetry (CV) of graphite fibers. (c) The corresponding CV curves at point F1 and F2 by convolution calculation of data in (b). (d) The time-varying concentration distribution of VO^{2+} in the CV of graphite fibers.

(3). Contribution of graphite fibers with different reaction rates to concentration variation.

In order to study the effect of electrode reaction rates on concentration variation of the electrolyte, four graphite fibers with different reaction rates (k_I^0: 8.5×10^{-6} m s^{-1}, k_{II}^0: 1.3×10^{-6} m s^{-1}, k_{III}^0: 6.8×10^{-7} m s^{-1}, k_{IV}^0: 3×10^{-9} m s^{-1})[3] in random distribution are chosen as shown in Supplementary Figure

14(a). It can be seen in Supplementary Figure 14(b) that the time-varying concentration of VO^{2+} in the CV of the graphite fiber with largest reaction rate decreases and recovers with the sharpest slope. Besides, in Supplementary Figure 14(c), the CV curve of the graphite fiber with largest reaction rate has the largest peak current density and the smallest peak potential separation value, which means that it has the best activity and reversibility. The time-varying VO^{2+} concentration distributions in the CV of graphite fibers with different reaction rates in Supplementary Figure 14(d) and Supplementary Video 9 show the effect of graphite fibers with different reaction rates on concentration variation.

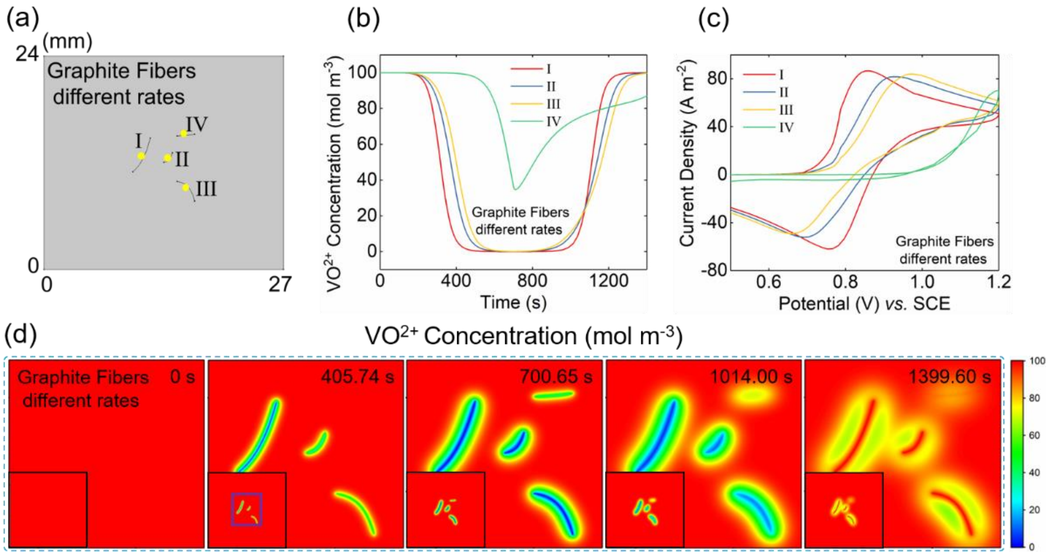

Supplementary Figure 14. (a) Horizontal Schematic of four graphite fibers (random distribution) with different reaction rates in the fluid reservoir. (b) The time-varying concentrations of VO^{2+} at point I, II, III, and IV in the cyclic voltammetry (CV) of graphite fibers. (c) The corresponding CV curves at at point I, II, III, and IV by convolution calculation of data in (b). (d) The time-varying concentration distribution of VO^{2+} in the CV of graphite fibers with different reaction rates.

Hence, as mentioned above, the CV process of the electrode in the work satisfies the initial condition, semi-infinite diffusion condition and flux balance condition, and thus this convolution relationship is obtained. Meanwhile, it is verified that the detection signal is contributed by the concentration variation of the electrolyte from the electrochemical reaction of the local graphite fibers within the local penetration depth of the evanescent field, and the concentration variation of the diffused electrolyte from the reaction of the graphite fibers within the diffusion layer.

7. Current density distribution in and out of the contact regions between the graphite fiber

and the prism.

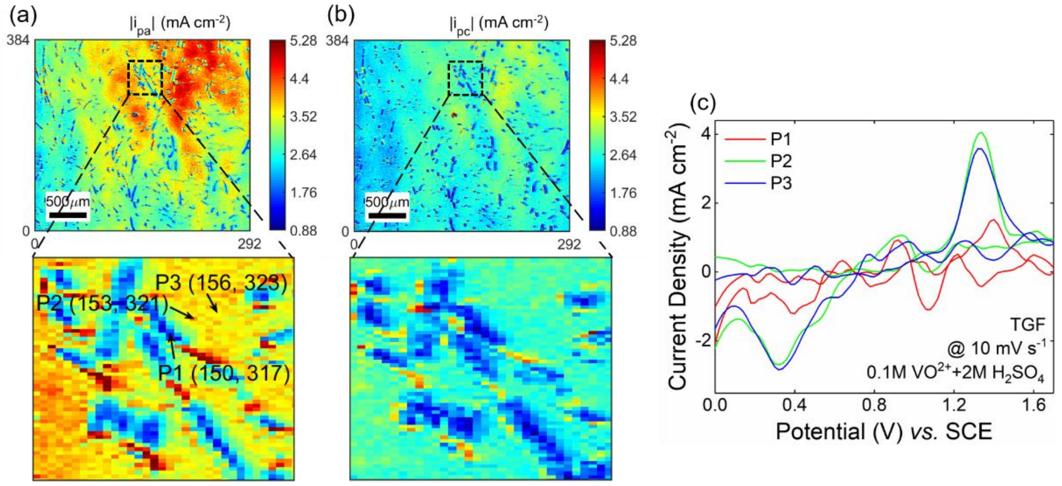

Supplementary Figure 15. The mappings of (a) the peak oxidation current densities ($|i_{pa}|$), (b) the peak reduction current densities ($|i_{pc}|$) and their enlarged views. (c) The cyclic voltammetry (CV) curves of the TGF in the positive electrolyte of 0.1 M VO^{2+} and 2 M H_2SO_4 at a scan rate of 10 mV s^{-1} at point P1, P2 and P3.

The method provided herein is based on the reflected intensity variations caused by the electrolyte concentration variations during the electrochemical reaction to obtain a current density distribution. Specifically, in a TIRi system, the concentration variation of the electrolyte causes a change in its refractive index. So the reflected intensity changes in the total internal reflection condition. The current density distribution is thus obtained by convolution calculation of the intensity variation. As the active site for electrochemical reaction, graphite felt should actually have a large current density. Since the total reflection at the areas where the graphite fiber in direct contact with the prism is not satisfied (refractive index: $n_{graphite} = 2.8363$; $n_{electrolyte} = 1.3566$; $n_{prism} = 1.75$ at the wavelength of 632.8 nm)[4] and there is no electrolyte, the method cannot give an accurate current density by the electrolyte concentration variations at these areas. Because the TIRi system is tuned with a suitable incidence angle based on the initial refractive index of the electrolyte, the method can provide current density distribution in the associated void areas immersed in the electrolyte. The current density of these areas is mainly from the surrounding graphite fibers, which can reflect the activity distribution of the surrounding graphite fibers. In addition, since the refractive indices of graphite fibers and electrolytes differ greatly, the image captured by the TIRi system can achieve higher contrast and better quality. Hence, the method can not only image the surface of the graphite felt, but also indirectly reflect the activity distribution of the graphite felt.

As shown in Supplementary Figure 3(c), it can be seen that the regions with graphite felt fiber contact have smaller sensitivity owing to the intensity beyond the almost linear range. This attributes to the fact that the lower reflected light intensity at the regions where the graphite fibers are in contact with the prism is not within the sensitivity linear range. On the contrary, since the intensity of the void region immersed by the electrolyte is in the linear range of the TIRi sensor, the void region is considered. The concentrations of vanadium ions with different valence states change and diffuse into the void region after the redox reaction of the electrolyte on the graphite fiber, so that the relative current density near the graphite fiber (the void region) can be obtained to reflect the activity of the graphite fibers at this region.

To further verify the above description, the mappings of (a) the peak oxidation current densities ($|i_{pa}|$), (b) the peak reduction current densities ($|i_{pc}|$) are enlarged as shown in Supplementary Figure 15(a, b). It can be seen that the current density at the contact region between the graphite fiber and the prism is low (the light intensity is not in the linear range) while the current density near the graphite fiber is higher. The CV curves of the thermal activated graphite felt (TGF) in the positive electrolyte of 0.1 M VO^{2+} and 2 M H_2SO_4 at a scan rate of 10 mV s^{-1} at point P1, P2 and P3 (labelled in Supplementary Figure 15(a)) are plotted in Supplementary Figure 15(c). Compared with CV curves at the void regions (P2, P3), the CV curve at the contact region between the graphite fiber and the prism (P1) has no obvious oxidation peak and reduction peak. So it is not suitable to compare the CV curves of the contact regions and the void regions in this work. The TIRi sensor not only provides imaging the fibers of the graphite felt, but also measures the activity distribution out of the contact regions between the graphite fiber and the prism. Ultimately, the goal is to reveal that the graphite fiber acts as a redox reaction site, and the electrolyte diffuses to the void area after the reaction.

8. Cyclic voltammetry detection of the TGF in the positive electrolyte with different concentrations of VO^{2+} and 2 M H_2SO_4.

CV measurement is conducted on the TGF in the positive electrolyte with different concentrations of VO^{2+} and 2 M H_2SO_4 at a scan rate of 2 mV s^{-1}, whose results recorded by the EW are plotted in Supplementary Figure 16(a). It is seen that the oxidation and reduction current densities raise gradually along with the increasing concentration of VO^{2+}. The intensity variation curves of a single

point A (43, 224) in the TGF with different concentrations of VO^{2+} and 2 M H_2SO_4 are recorded by the TIRi sensor and displayed in Supplementary Figure 16(b). By denoising and deconvolution calculation of the data in Supplementary Figure 16(b), the CV curves of the single point A (43, 224) in the TGF are plotted in Supplementary Figure 16(c), which are in accordance with that recorded by the EW. By extracting the peak oxidation current densities i_{pa}, the peak reduction current densities i_{pc} and their corresponding potentials from the CV curves recorded by the EW and by the TIRi sensor, the i_{pa} and i_{pc} at different concentrations of VO^{2+} are plotted in Supplementary Figure 16(d). Besides, the $|i_{pc}/i_{pa}|$ and the peak potential separation value $|\Delta E|$ at different concentrations of VO^{2+} are plotted in Supplementary Figure 16(e, f), respectively. The consistency of the data in Supplementary Figure 16(d - f) recorded by the EW and by the TIRi sensor determines the feasibility of the TIRi sensor. The mappings of the peak oxidation current densities $|i_{pa}|$ at different concentrations of VO^{2+} are displayed in Supplementary Figure 16(g), which shows the distinguished distributions of the peak oxidation current densities.

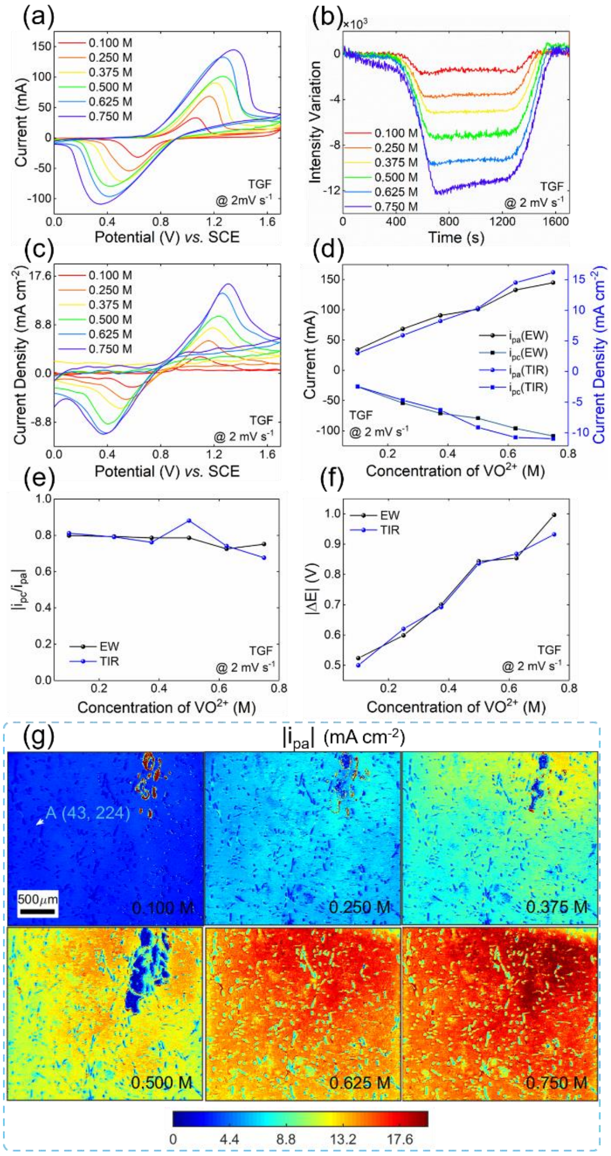

Supplementary Figure 16. (a) CV curves of the TGF in the positive electrolyte with different concentrations of VO^{2+} and 2 M H_2SO_4 at a scan rate of 10 mV s^{-1} recorded by the electrochemical workstation (EW). (b) Intensity variations of point A (43, 224) recorded by the TIRi sensor during the CV at different concentrations of VO^{2+}. (c) CV curves by convolution calculation of the data in (b) at different concentrations of VO^{2+}. (d) The peak oxidation current densities i_{pa} and the peak reduction current densities i_{pc} measured by the EW and the TIRi sensor at different concentrations of VO^{2+}. (e) The $|i_{pc}/i_{pa}|$ measured by the EW and the TIRi sensor at different concentrations of VO^{2+}. (f) The peak potential separation value $|\Delta E|$ measured by the EW and the TIRi sensor at different concentrations of VO^{2+}. (g) The mappings of the peak oxidation current densities $|i_{pa}|$ at different concentrations of VO^{2+}.

9. Region of interests (ROI) for current density curves.

As shown in Supplementary Figure 17(a), the size of an image captured by CCD is 1460 × 1920 pixels. In order to reduce the intensity noise, the intensities of each 5×5 pixels are averaged into the

intensity of one point as shown in the enlarged views of Supplementary Figure 17(a, b). We label each point as region of interest (ROI).

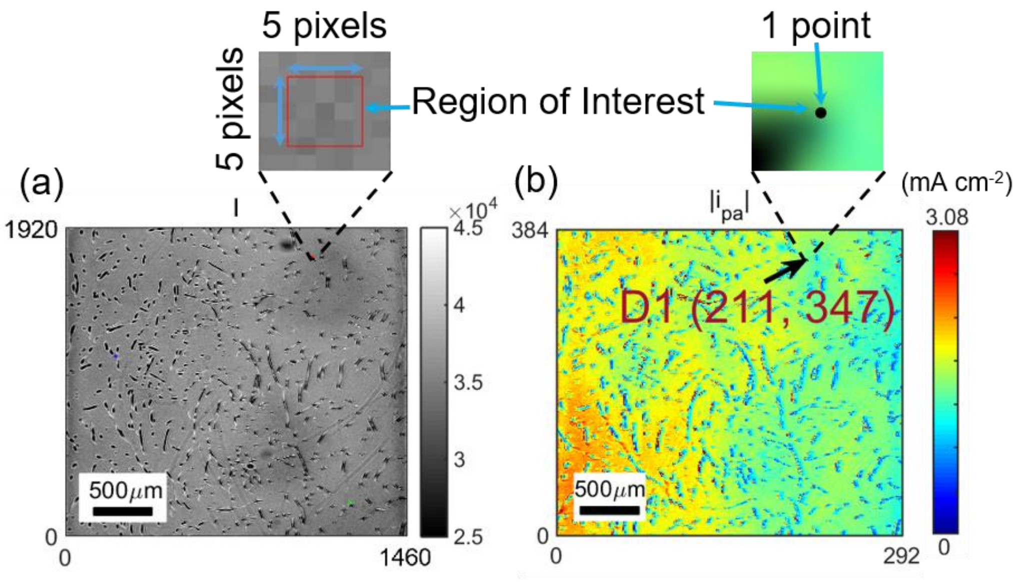

Supplementary Figure 17. (a) An image of the GF captured by CCD. (b) The mapping of the peak oxidation current densities $|i_{pa}|$ of the GF at the VO^{2+} concentrations of 0.100 M.

(1). Region of interest for the curves in Fig. 3b.

The time-varying intensities of point D1 (211, 347) (randomly selected point, labelled in Supplementary Figure 17(b)) for the GF, TGF and PGF are obtained. The intensity variations of the single point D1 (211, 347) are plotted in Figure 3(b) by subtracting from the initial intensity from the obtained time-varying intensities. The point D1 (211, 347) is the region of interest to obtain the curves in Fig. 3b.

(2). Comparison of full image based curves and segment based local curves.

To further study the difference of optical curves between the full image and the segment, the intensity variations and CV curves from full-image and the segment are plotted in Supplementary Figure 18. The intensity variations of the GF, TGF and PGF of point D1 (211, 347) recorded by the TIRi sensor and the corresponding CV curves are with noise while that of the full image are smooth. The local intensity curves and current density curves are similar with those from entire view-of-field (full-image).

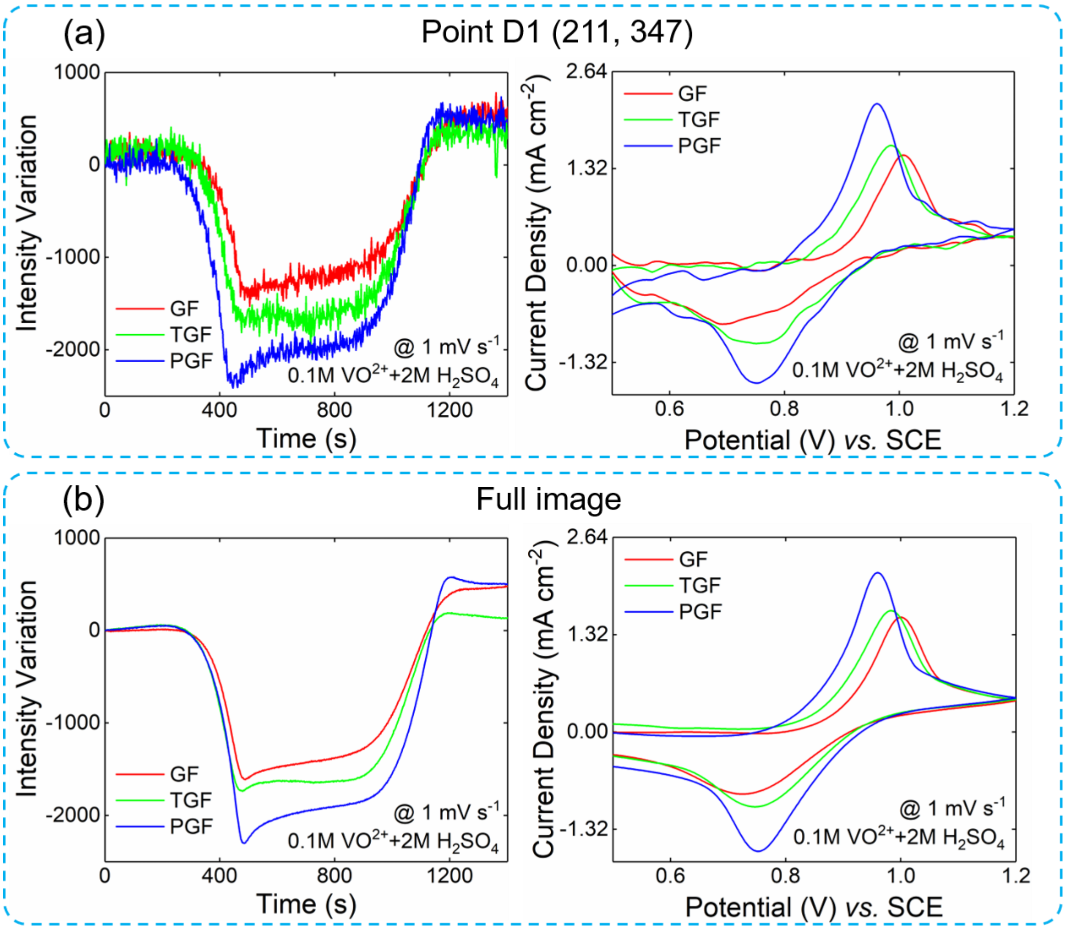

Supplementary Figure 18. TIR intensity variations of the GF, TGF and PGF recorded by the TIRi sensor and the corresponding CV curves of (a) point D1 and (b) the full image.

10. The electrochemical activity distribution of the thin surface layer of graphite fibers.

The electroactive area of a bulk electrode (graphite felt) is the surface areas of graphite felt fibers that participate in electrochemical redox reaction (as the electrochemical reaction sites). Traditional electrochemical techniques to measure it are cyclic voltammetry, chronovoltammetry and so on. For example, in the cyclic voltammetry process, the scan rate is changed to obtain different CV curves. According to the relationship of the peak current in proportion to the square root of the scan rate, the electroactive area of a bulk electrode is obtained. Besides, in the chronovoltammetry process, the electroactive area of a bulk electrode can also be obtained by the Cottrell equation[1].

As shown in Supplementary Figure 19, graphite felt is a porous structure. The thickness of the graphite felt used in this work is 5.4 mm. According to the literature which is widely cited[3], the diffusion coefficient of VO^{2+} - VO_2^+ reaction on carbon electrodes is D = 3.9×10^{-10} m^2 s^{-1}. So the diffusion length is $L = \sqrt{2Dt_{max}} = \sqrt{2 \times 3.9 \times 10^{-10} \times 2 \times (1.2 - 0.5)/0.001}$ (m) = 1.0450

(mm). The TIRi sensor only images a portion of the fibers in contact with the prism (the penetration depth: 938 nm) on the outer surface of the graphite felt (red trapezoid in Supplementary Figure 19(a)) and provides the local current density, which is affected by the fibers in the diffusion layer (thickness: the diffusion length ~1 mm), including local current and diffusion current. For example, the reactant and product concentrations at the point G within the penetration depth is affected by electrochemical reaction from the local fibers and the diffusion process within the diffusion length (semi-sphere C in Supplementary Figure 19(b)). The current density at the point G has the same situation. Therefore, the image reflects the electrochemical activity distribution of the thin surface layer of graphite fibers.

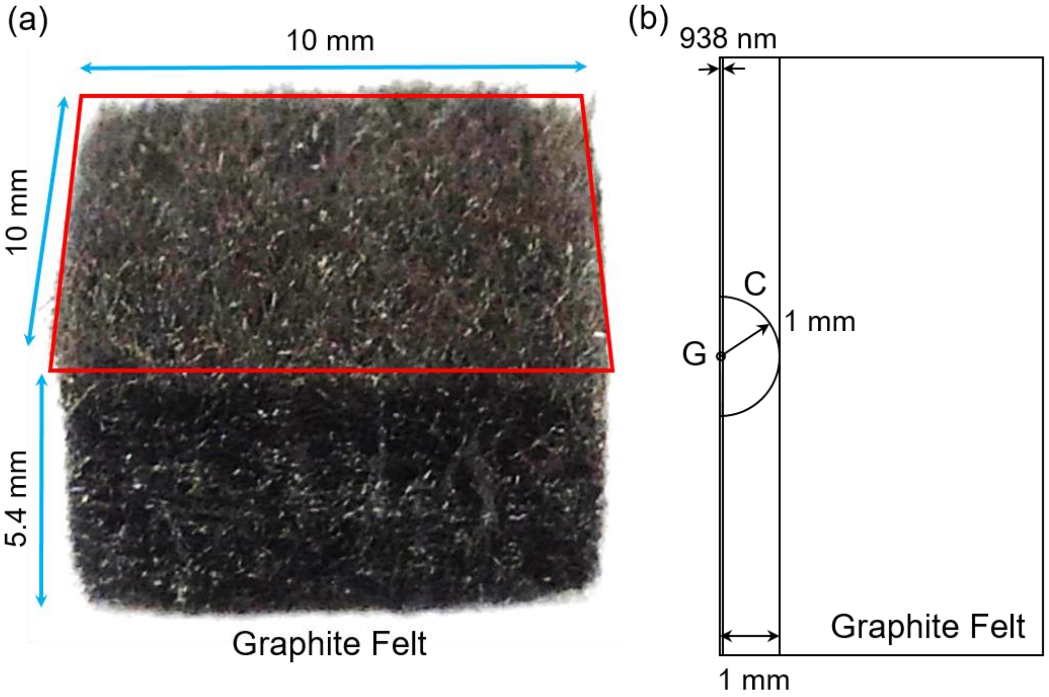

Supplementary Figure 19. (a) Photograph of a graphite felt. (b) Schematic of a graphite felt. The penetration depth of the incident light: 938 nm. The diffusion length: 1 mm.

11. Parallel measurements of current density distribution by the TIRi sensor.

In order to provide statistical data and stability of the sensor in multiple measurements, we have performed the CV experiment (the positive electrolyte: 0.1 M VO^{2+} and 2 M H_2SO_4; the potential window: 0.5 - 1.2 V; scan rate: 1 mV s^{-1}) on four GFs (10 × 10 × 5.4 mm^3) from the same batch of graphite felts respectively and the images are captured at four different view of fields for each GF. So there are 16 measurements for GFs and four parameters ($|i_{pa}|$, $|i_{pc}|$, $|i_{pc}/i_{pa}|$ and $|\Delta E|$) for each measurement. The same experiments are performed on TGFs and PGFs. As shown in

Supplementary Figure 20(a, d, g), the peak oxidation current densities $|i_{pa}|$ of the GF, TGF and PGF are mapped at four different view of fields (1, 2, 3 and 4) respectively and their distributions are plotted in Supplementary Figure 20(b, e, h). By Gaussian fitting of the distributions, their peak positions and full widths at half maximum (FWHM) are quantitatively shown in Supplementary Figure 20(c, f, i). As shown in Supplementary Figure 20(b, e, h), the $|i_{pa}|$ distributions are relatively consistent at different view of fields. At the same time, by comparing the $|i_{pa}|$ values of GF, TGF and PGF, it can be seen that the $|i_{pa}|$ differences of GF, TGF and PGF can be distinguished from the small $|i_{pa}|$ differences at different view of fields. Meanwhile, it can be obtained that the PGF has the highest activity and GF has the lowest. The similar distinguished results of $|i_{pc}|$, $|i_{pc}/i_{pa}|$ and $|\Delta E|$ are shown in Supplementary Figure 21 - 23.

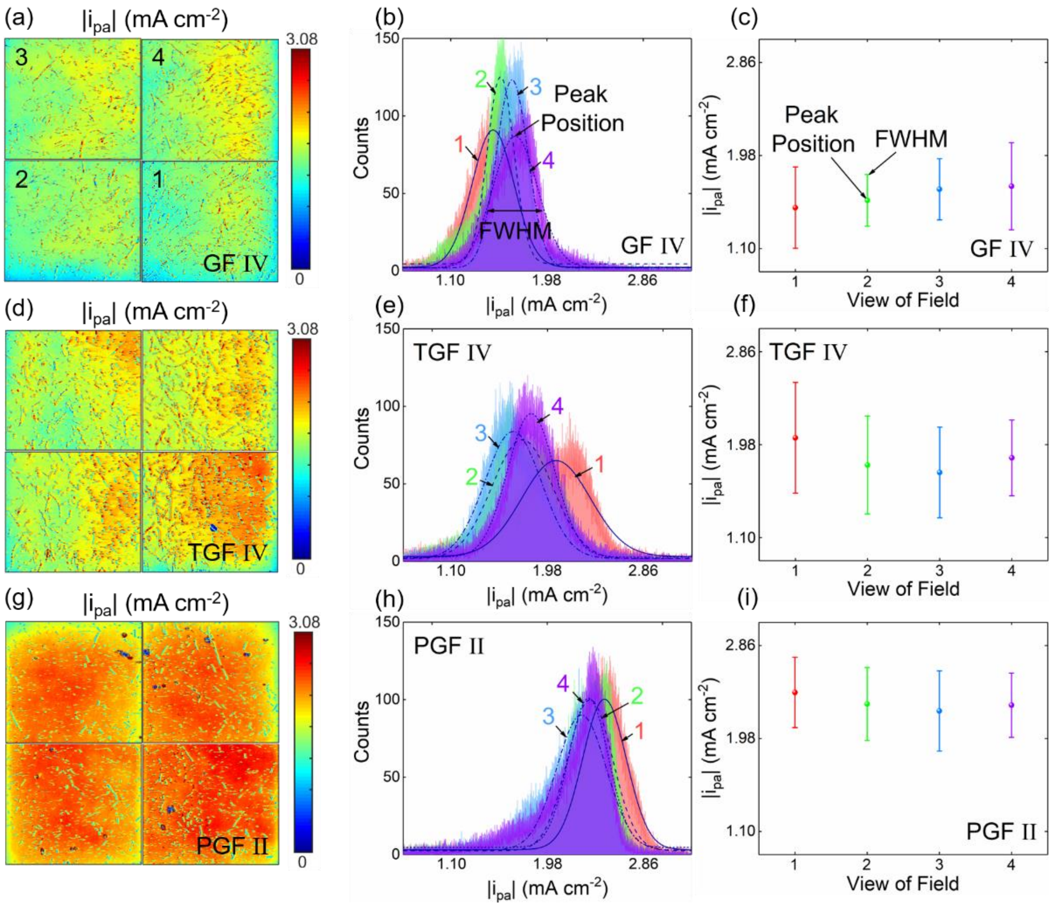

Supplementary Figure 20. The mapping of the peak oxidation current densities $|i_{pa}|$ of the (a) GF, (d) TGF and (g) PGF at four different view of fields (1, 2, 3 and 4) by the TIRi sensor respectively (positive electrolyte: 0.1 M VO^{2+} and 2 M H_2SO_4; scan rate: 1 mV s^{-1}). The $|i_{pa}|$ distributions of the (b) GF, (e) TGF and (h) PGF at four different view of fields respectively. The peak position and FWHM comparison of $|i_{pa}|$ distributions of the (c) GF, (f) TGF and (i) PGF at four different view of fields.

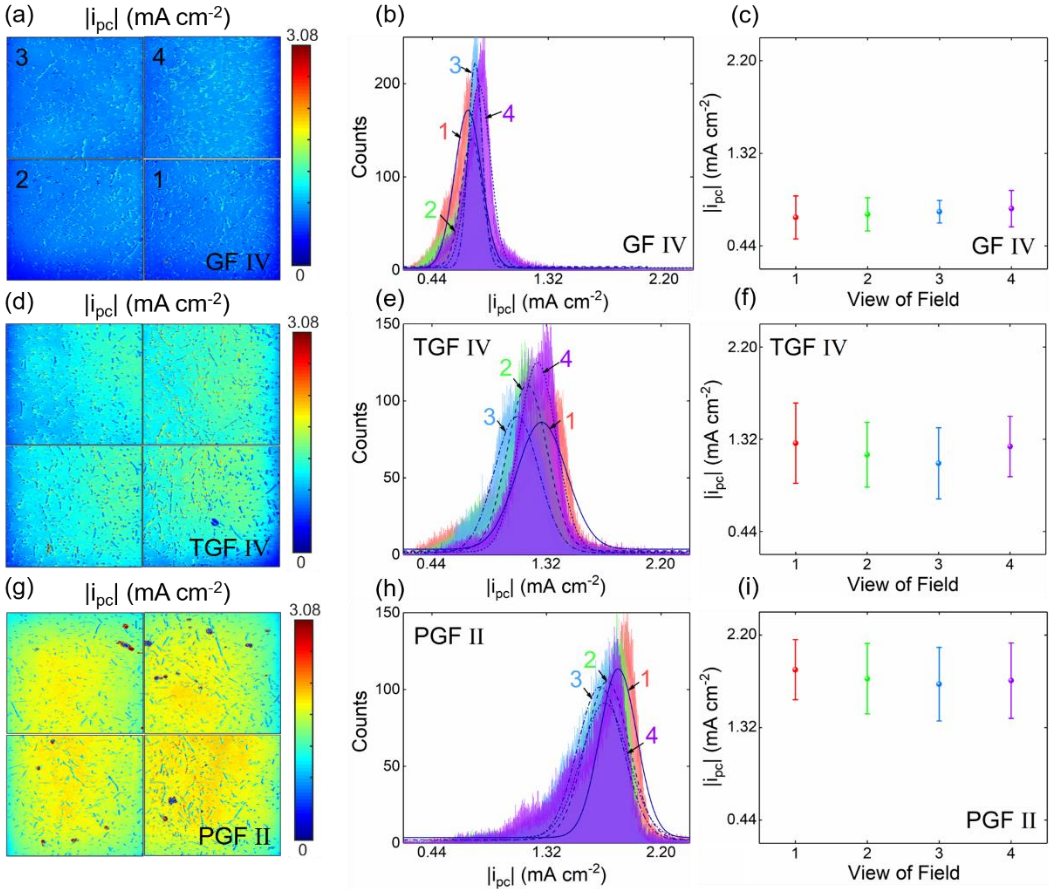

Supplementary Figure 21. The mapping of the peak reduction current densities $|i_{pc}|$ of the (a) GF, (d) TGF and (g) PGF at four different view of fields (1, 2, 3 and 4) by the TIRi sensor respectively (positive electrolyte: 0.1 M VO^{2+} and 2 M H_2SO_4; scan rate: 1 mV s^{-1}). The $|i_{pc}|$ distributions of the (b) GF, (e) TGF and (h) PGF at four different view of fields respectively. The peak position and FWHM comparison of $|i_{pc}|$ distributions of the (c) GF, (f) TGF and (i) PGF at four different view of fields.

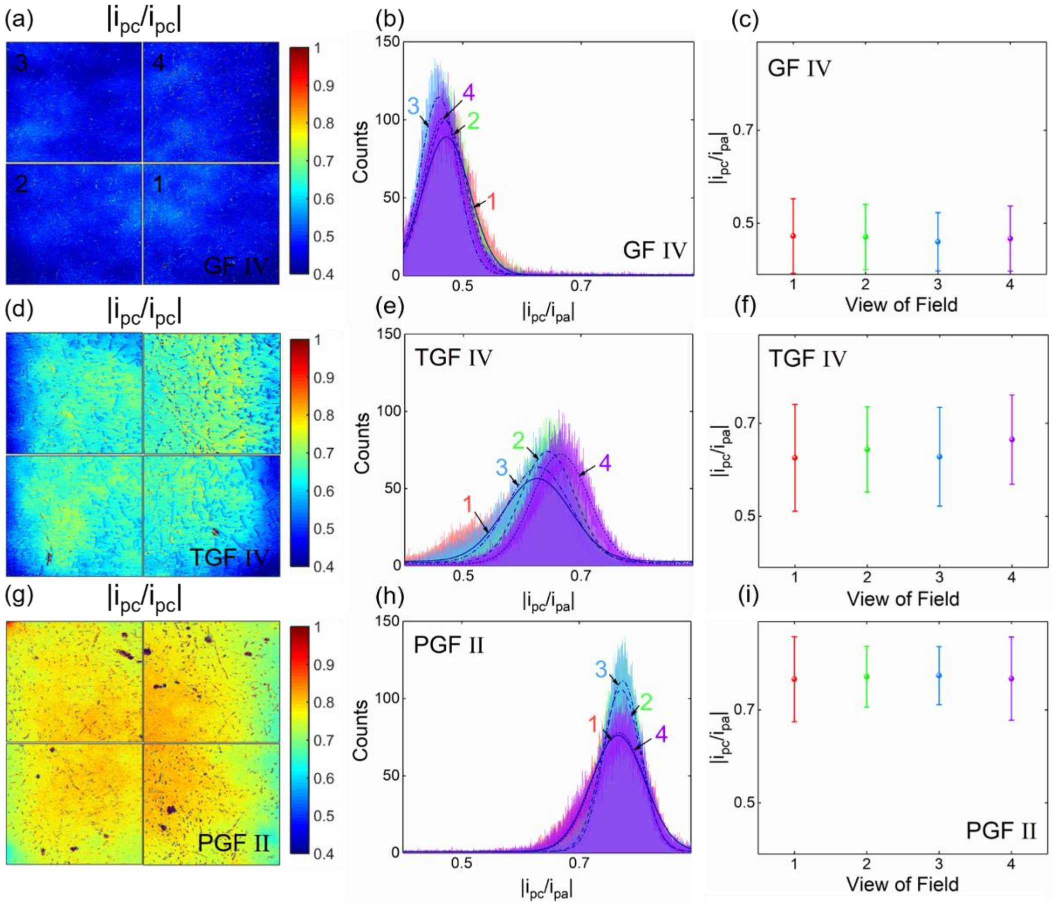

Supplementary Figure 22. The $|i_{pc}/i_{pa}|$ mapping of the (a) GF, (d) TGF and (g) PGF at four different view of fields (1, 2, 3 and 4) by the TIRi sensor respectively (positive electrolyte: 0.1 M VO^{2+} and 2 M H_2SO_4; scan rate: 1 mV s^{-1}). The $|i_{pc}/i_{pa}|$ distributions of the (b) GF, (e) TGF and (h) PGF at four different view of fields respectively. The peak position and FWHM comparison of $|i_{pc}/i_{pa}|$ distributions of the (c) GF, (f) TGF and (i) PGF at four different view of fields.

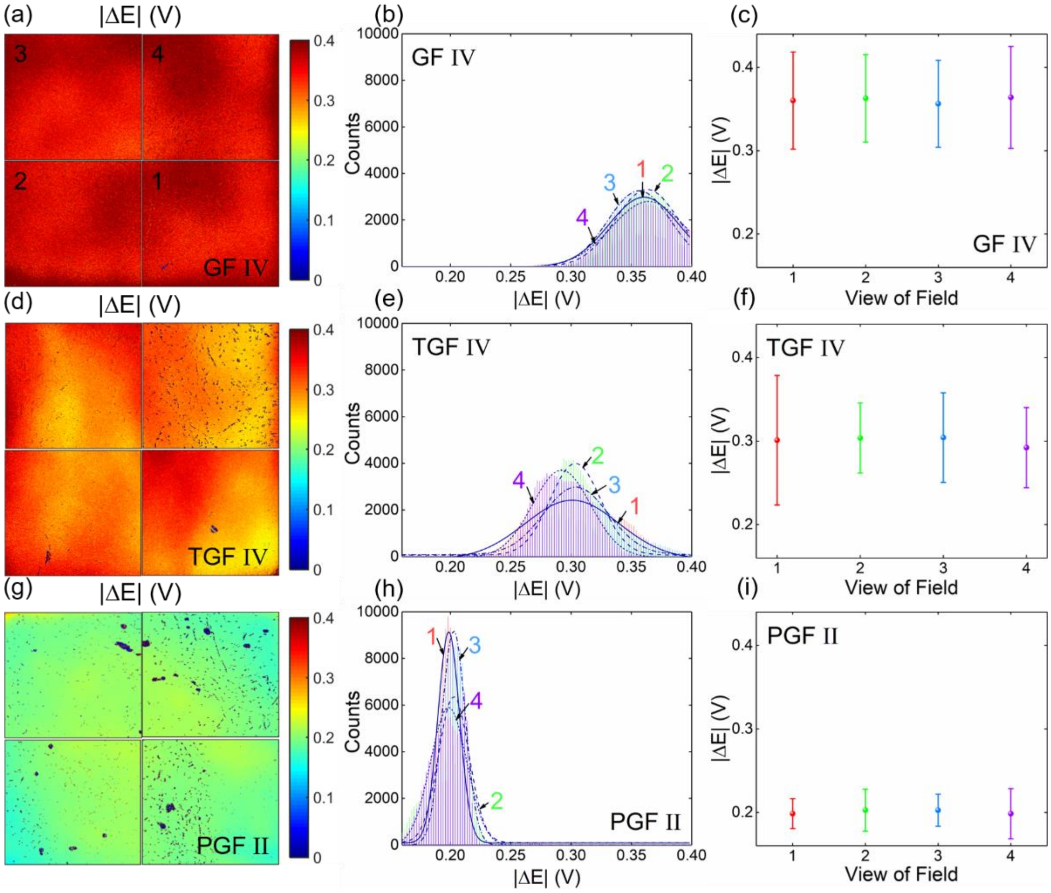

Supplementary Figure 23. The mapping of the peak potential separation value |ΔE| of the (a) GF, (d) TGF and (g) PGF at four different view of fields (1, 2, 3 and 4) by the TIRi sensor respectively (positive electrolyte: 0.1 M VO^{2+} and 2 M H_2SO_4; scan rate: 1 mV s^{-1}). The |ΔE| distributions of the (b) GF, (e) TGF and (h) PGF at four different view of fields respectively. The peak position and FWHM comparison of |ΔE| distributions of the (c) GF, (f) TGF and (i) PGF at four different view of fields.

By combining the results of the parallel measurements of four graphite felts from the same batch at four different view of fields respectively, it can be seen that the peak positions of four parameters ($|i_{pa}|$, $|i_{pc}|$, $|i_{pc}/i_{pa}|$ and |ΔE|) of each graphite felt from the same batch are relatively consistent, and the four parameters between GF, TGF and PGF can be effectively distinguished. Because the FWHM between GF, TGF and PGF reflects the activity and reversibility differences of the graphite felt itself at the same view of field, it can be accepted that the overlap of the FWHM between GF, TGF and PGF exits in Supplementary Figure 24.

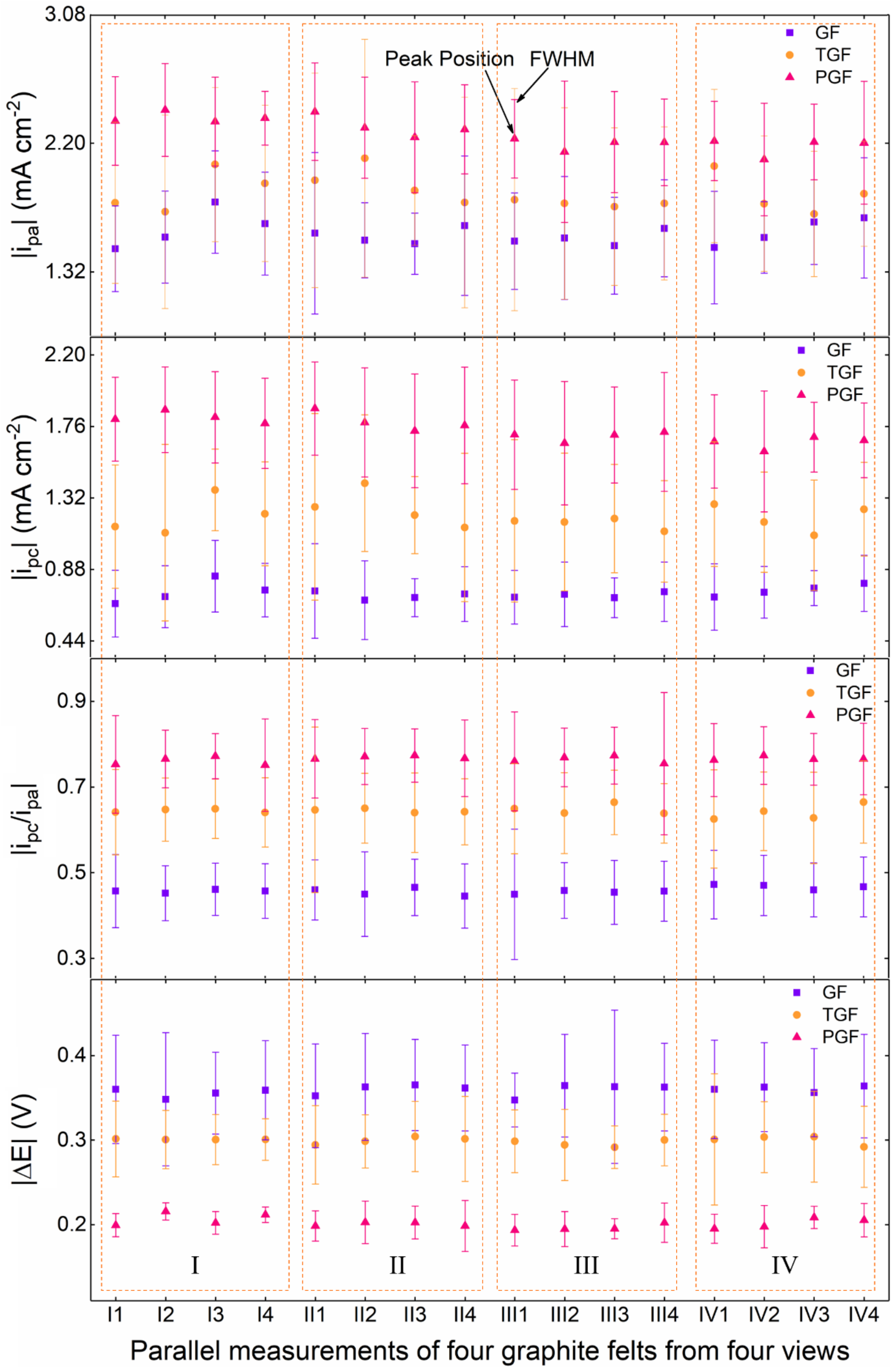

Supplementary Figure 24. The peak position and FWHM comparison of $|i_{pa}|$, $|i_{pc}|$, $|i_{pc}/i_{pa}|$ and $|\Delta E|$ distributions of the four GFs, four TGFs and four PGFs (I, II, III and IV) from the same batches of graphite felts at four different view of fields (1, 2, 3 and 4).

To measure the variations of the four parameters among different view of fields, the average and standard deviation (Std) of $|i_{pa}|$, $|i_{pc}|$, $|i_{pc}/i_{pa}|$ and $|\Delta E|$ distributions of the four GFs, four TGFs and four PGFs (I, II, III and IV) from the same batches of graphite felts are obtained as shown in Supplementary Figure 25. The variations of the four parameters among different view of fields do not affect the activity discrimination of GF, TGF and PGF. For example, as shown in Supplementary Figure 25(a), the variations of $|i_{pa}|$ among different view of fields are smaller than the $|i_{pa}|$ differences between GF, TGF and PGF.

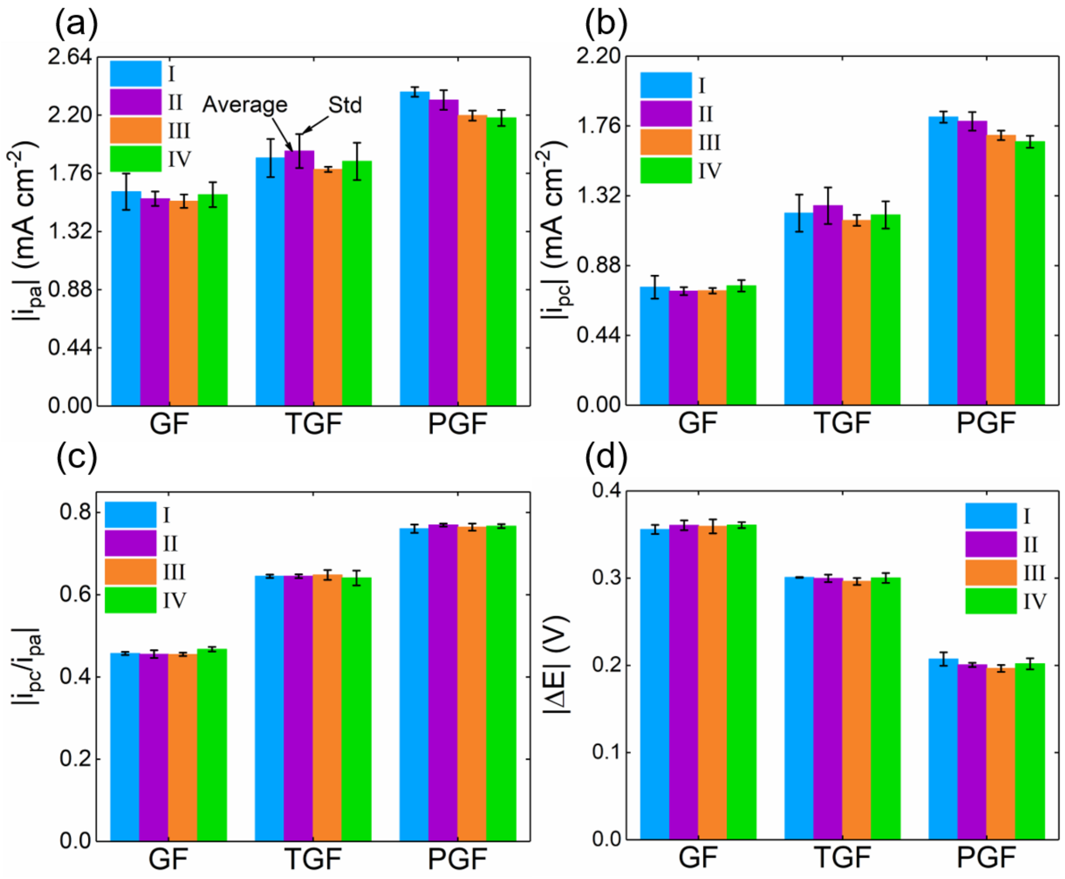

Supplementary Figure 25. The average and standard deviation (Std) comparison of (a) $|i_{pa}|$, (b) $|i_{pc}|$, (c) $|i_{pc}/i_{pa}|$ and (d) $|\Delta E|$ distributions of the four GFs, four TGFs and four PGFs (I, II, III and IV) from the same batches of graphite felts. Average: the average of the peak positions of $|i_{pa}|$, $|i_{pc}|$, $|i_{pc}/i_{pa}|$ and $|\Delta E|$ distributions at four different view of fields. Std: the standard deviation of the peak positions of $|i_{pa}|$, $|i_{pc}|$, $|i_{pc}/i_{pa}|$ and $|\Delta E|$ distributions at four different view of fields.

To identify the variation among multiple parallel measurements from the same batch of graphite felts, the average and Std of $|i_{pa}|$, $|i_{pc}|$, $|i_{pc}/i_{pa}|$ and $|\Delta E|$ of the four GFs, four TGFs and four PGFs (I, II, III and IV) from the same batches of graphite felts are compared and plotted in Supplementary

Figure 26. It can be seen that the variations of $|i_{pa}|$, $|i_{pc}|$, $|i_{pc}/i_{pa}|$ and $|\Delta E|$ among multiple parallel measurements from the same batches of graphite felts are smaller than the $|i_{pa}|$, $|i_{pc}|$, $|i_{pc}/i_{pa}|$ and $|\Delta E|$ differences between GF, TGF and PGF. It indicates that measurement results are relatively consistent under different view of fields and parallel measurements from the same batches of graphite felts. So this method is capable of distinguishing the activity and reversibility differences of GF, TGF and PGF.

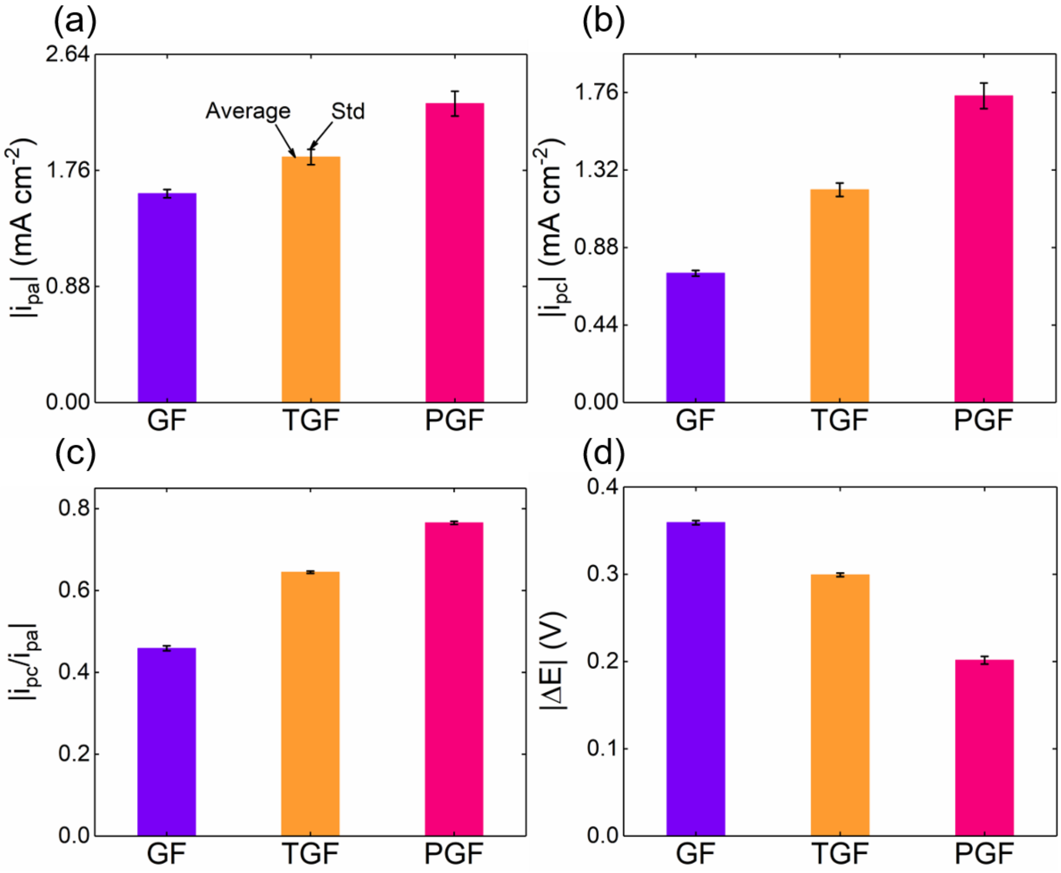

Supplementary Figure 26. The average and standard deviation (Std) comparison of (a) $|i_{pa}|$, (b) $|i_{pc}|$, (c) $|i_{pc}/i_{pa}|$ and (d) $|\Delta E|$ distributions of the GF, TGF and PGF. Average: the average of $|i_{pa}|$, $|i_{pc}|$, $|i_{pc}/i_{pa}|$ and $|\Delta E|$ of the four GFs, four TGFs and four PGFs (I, II, III and IV) from the same batches of graphite felts. Std: the standard deviation of $|i_{pa}|$, $|i_{pc}|$, $|i_{pc}/i_{pa}|$ and $|\Delta E|$ of the four GFs, four TGFs and four PGFs (I, II, III and IV) from the same batches of graphite felts.

12. Different potential windows in the long-term cyclic voltammetry.

The purpose of selecting different potential windows is:

(1). To determine the onset potential by directly counting the number of bubbles, it is necessary to start the oxygen evolution reaction under a narrow potential window (such as: 0 V - 1.7 V). So the

bubble generation is more moderate, which is convenient for statistical calculation of bubbles.

(2). To study the relationship between the oxygen evolution reaction and the electrochemical activation effect of the electrode, the oxygen evolution reaction in a large potential window is required to generate a large number of oxygen bubbles, resulting in a higher degree of electrode activation.

Therefore, this work selects two different potential windows to achieve the above purpose.

As shown in Supplementary Figure 27, in the potential scan range of 0 V - 1.7 V, the oxygen evolution reaction (dashed rectangle in Supplementary Figure 27(a)) in the CV process has just started, and bubble generation is moderate for the current is relatively small. The counts of bubbles in long-term CV are plotted in Supplementary Figure 28. Supplementary Figure 28(a) shows the time-varying counts of bubbles in the 1st - 16th cycles, which gradually increase accompanied by periodic fluctuations and finally reach a stable equilibrium state. The time-varying counts of bubbles (Supplementary Figure 28(b)) in the 8th - 10th cycles show the periodical increase and decrease. By counting the bubbles， the onset potential of the OER can be determined. It indicates that the TIRi sensor has potential to determine the onset potential of the OER by mapping the bubble kinetics directly. However, when the potential scan range is enlarged to 0 V - 2.0 V, the oxygen evolution reaction is more intense and the current is relatively large (dashed rectangle in Supplementary Figure 27(b)). The time-varying counts of bubbles in the 1st - 16th cycles are plotted in Supplementary Figure 28(c) and that in the 8th - 10th cycles with strong jitter are plotted in Supplementary Figure 28(d), which cannot be used to determine the onset potential of the OER. In order to further study the relationship between the activity of the graphite felt and the oxygen evolution reaction, the potential scan range is enlarged to 0 V - 2.0 V. The larger potential scan window can intensify the oxygen evolution reaction and increase the electrochemical activation effect of the graphite felt. Supplementary Video 1 and Supplementary Video 3 show the difference of the time-varying counts of bubbles in the potential window of 0 V - 1.7 V and 0 V - 2.0 V respectively.

Hence, the potential scan range of 0 V - 1.7 V is for studying the bubble kinetics of the oxygen evolution reaction while that of 0 V - 2.0 V is for studying the activity of the graphite felt with the oxygen evolution reaction.

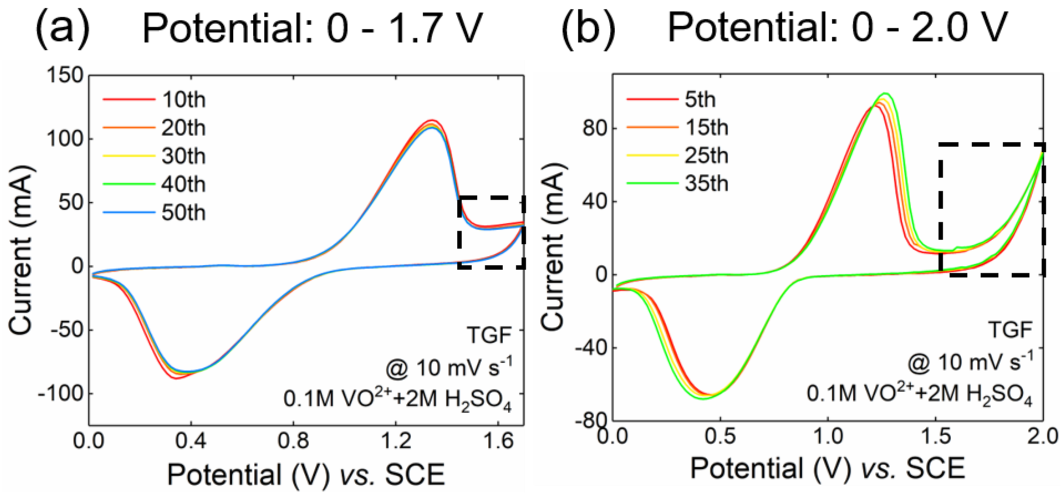

Supplementary Figure 27. CV curves of the TGF in the positive electrolyte with 0.1 M VO^{2+} and 2 M H_2SO_4 at a scan rate of 10 mV s^{-1} recorded by the electrochemical workstation (EW): (a) 0 V - 1.7 V; (b) 0 V - 2.0 V.

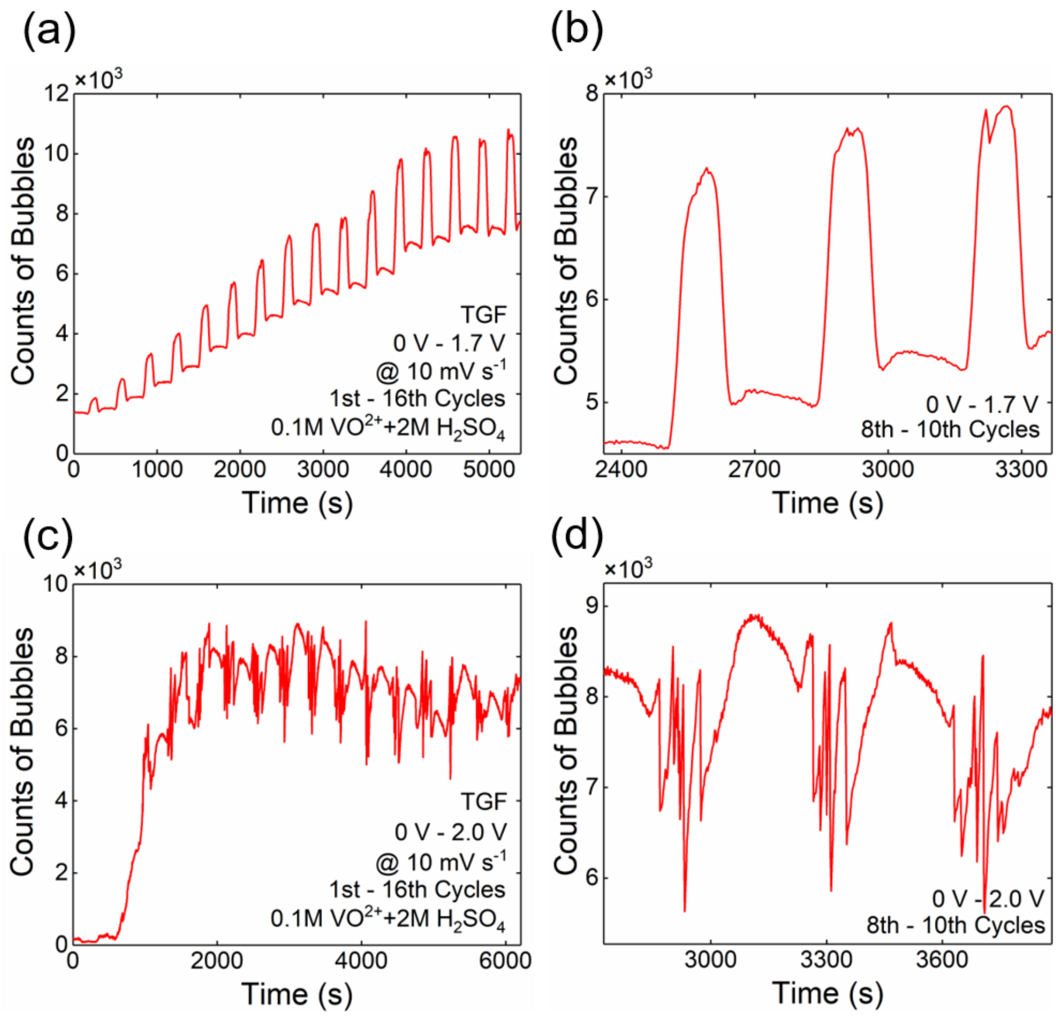

Supplementary Figure 28. The counts of bubbles in the 1st to 16th cycles during long-term CV of the TGF in the positive electrolyte of 0.1 M VO^{2+} and 2 M H_2SO_4 at a scan rate of 10 mV s^{-1} recorded by the TIRi sensor at the

potential window of (a) 0 V - 1.7 V and (c) 0 V - 2.0 V. The enlarged view of the counts of bubbles in the 8th to 10th cycles at the potential window of (b) 0 V - 1.7 V and (d) 0 V - 2.0 V.

13. Comparison of the bubble distributions from the local bubble dynamics and the onset potentials of oxygen evolution/reduction reaction at different cycles.

In order to confirm the bubble dynamics, we compare the bubble distributions from the local bubble dynamics, and the onset potentials of oxygen evolution/reduction reaction at different cycles.

(1). Three types of the local bubble dynamics.

As shown in Supplementary Figure 29(a), we label three rectangles (red; green; blue) as regions of interest to compare the bubble generation and distribution in different regions. There are three types of bubble dynamics in the long-term CV experiment, which is the same with that in Figure 4. The first type (red rectangle in Supplementary Figure 29(a)) is shown in Supplementary Figure 29(b). The bubbles appear and grow from the first cycle as the bubbles are in contact with the prism. Then, the time-varying counts of bubbles gradually increase, and fluctuate periodically. Finally, the equilibrium state is reached along with periodic fluctuation. The second type (green rectangle in Supplementary Figure 29(a)) is shown in Supplementary Figure 29(c). The bubbles are not imaged in the first few cycles because they are not in contact with the prism, but they continue growing to be in contact with the prism and detected by CCD. Then they also gradually grow larger and finally reach the equilibrium state accompanied by periodic fluctuation. The third type (blue rectangle in Supplementary Figure 29(a)) is shown in Supplementary Figure 29(d). The bubbles are also not imaged in the first few cycles because they are not in contact with the prism, but they grow up to be detected in contact with the prism accompanied by periodic fluctuation and then come out of contact with the prism, so that they are not imaged in the last few cycles. Hence, in order to effectively study the bubble dynamics by observing the change in the counts of bubbles at any time, the time periods when the local bubble is out of contact with the prism in Supplementary Figure 29(c, d) can be avoided as much as possible if the bubbles are counted in the statistics from the full image.

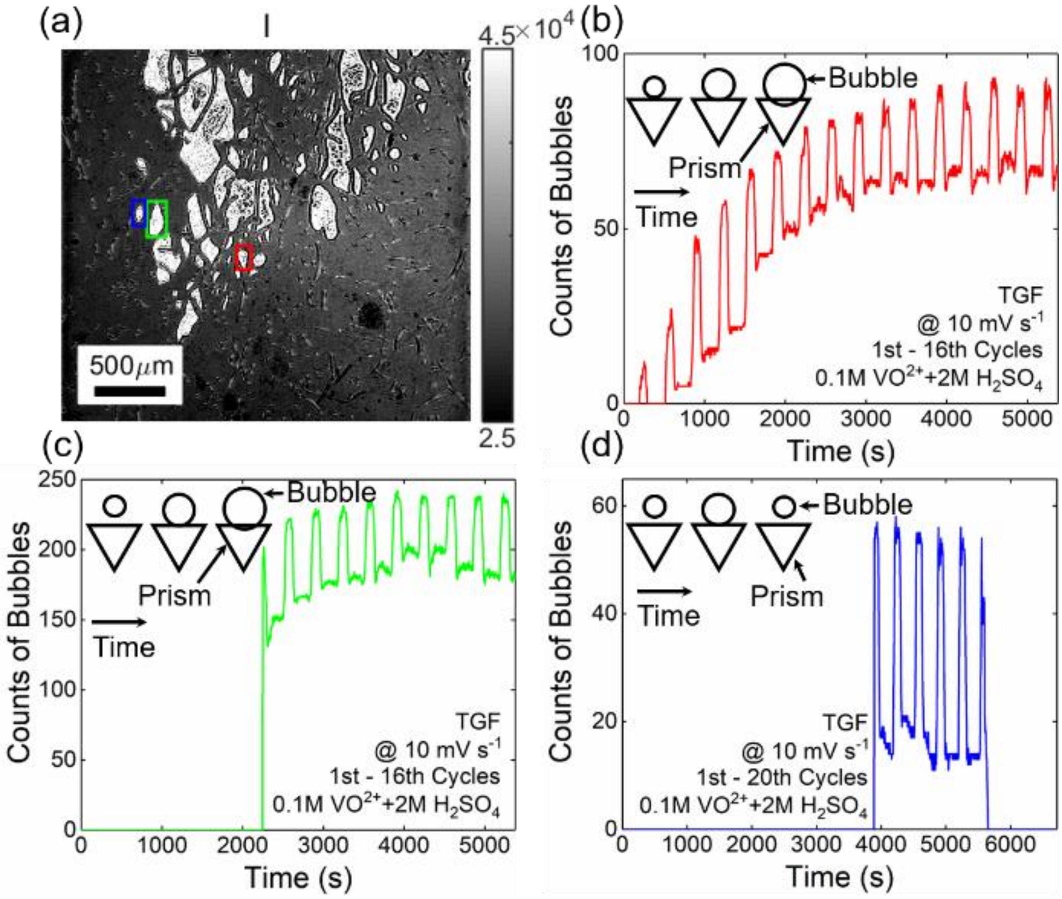

Supplementary Figure 29. (a) One image captured by CDD in the long-term CV. The red, green and blue rectangles are regions of interest to compare the bubble distribution dynamics. (b, c, d) Three types of bubble dynamics in the long-term CV.

(2). The onset potentials of oxygen evolution/reduction reaction at different cycles.

In order to further verify the feasibility of visually determining the onset potential of the oxygen evolution/reduction reaction by the counts of bubbles, we have plotted the kinetic curves of the counts of bubbles in different cycles (e.g. 5th, 10th, 15th, 20th, 25th, 35th, 45th) during long-term CV as shown in Supplementary Figure 30(a) and pointed out their respective onset oxygen generation/consumption potentials. The results show that the kinetic curves of the counts of bubbles and the obtained onset potentials are relatively consistent. This preliminary result indicates the feasibility of the above method to determine the onset potential of the oxygen evolution/reduction reaction. Furthermore, the specific values of the onset oxygen generation/consumption potentials are shown in Supplementary Figure 30(b). The relatively consistence of these data further indicates the feasibility of the method. Meanwhile, the difference of these data comes from methodological noise, data processing noise on the one hand, and the variation of electrode activity during long-

period cycles on the other hand.

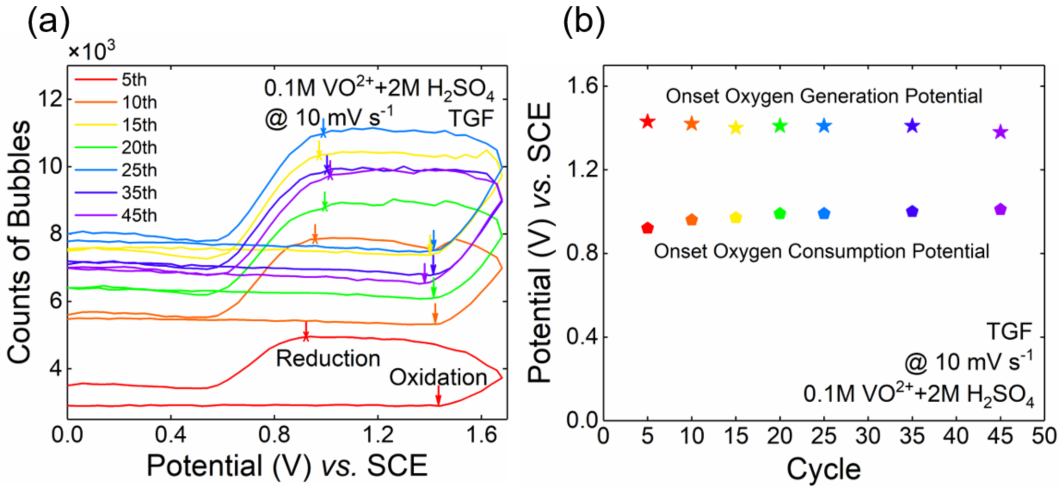

Supplementary Figure 30. (a) The counts of bubbles via the sweep potential in the different CV cycles (the electrode: TGF; the positive electrolyte: 0.1 M VO^{2+} and 2 M H_2SO_4; the scan rate: 10mV s^{-1}). Arrows point out the onset oxygen generation/consumption potentials. (b) The onset oxygen generation/consumption potentials in the different cycles.

14. Bubble generation in the region with high activity.

As shown in Supplementary Figure 31, the region labelled by four dashed rectangles is the region R with comparatively high $|i_{pa}|$. According to Figure 4(c) or Supplementary Video 1, the bubbles from the oxygen evolution reaction appear on the region R. It means that the region R with high activity is more likely to generate bubbles. As the onset oxygen evolution potential is among the potential scan range (0 V - 1.7 V, Figure 4(b)) and the counts of bubbles in the 1st cycle (Figure 4(a)), the oxygen evolution reaction begins at the 1st cycle.

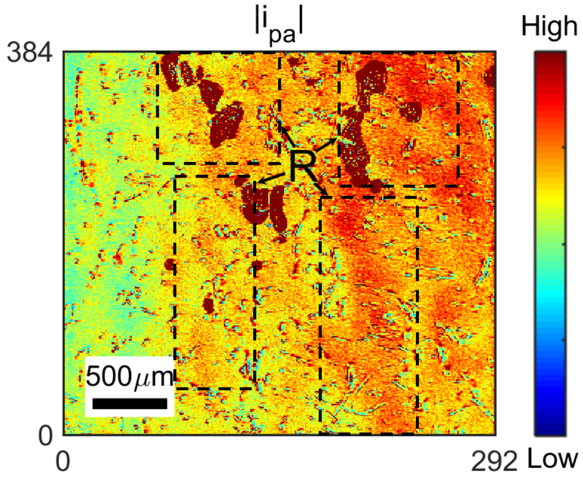

Supplementary Figure 31. The mapping of the peak oxidation current densities $|i_{pa}|$ of the TGF in the 1st cycle.

Four dashed rectangles: the region R with comparatively high $|i_{pa}|$.

15. The influence of the bubble on the local electrochemical reactions.

In the long-term CV, bubbles would block the local electrochemical reaction (abbreviated as: BBR), as can be seen from the first few cycles (1st-5th cycles in Supplementary Figure 32) in long-term CV. In detail, the peak oxidation/reduction currents of the CV curves in the first few cycles gradually become smaller in Supplementary Figure 32(b, c, d), because the bubble generation at the beginning is in the growth stage, and thus the BBR effect is greater than the electrochemical activation effect (owing to the generation of the oxygen-containing functional group on the electrode).

As the cycles continue, the counts of bubbles become saturated and reach a stable equilibrium state as shown in Figure 4(a). It means that the BBR effect reaches the limit. At the same time, the electrochemical activation effect gradually increases and also reaches the limit. However, compared with the BBR effect, the electrochemical activation effect dominates at this time, so that the net effect makes the peak oxidation/reduction currents of the CV curve increase in Supplementary Figure 32(d) and Figure 5.

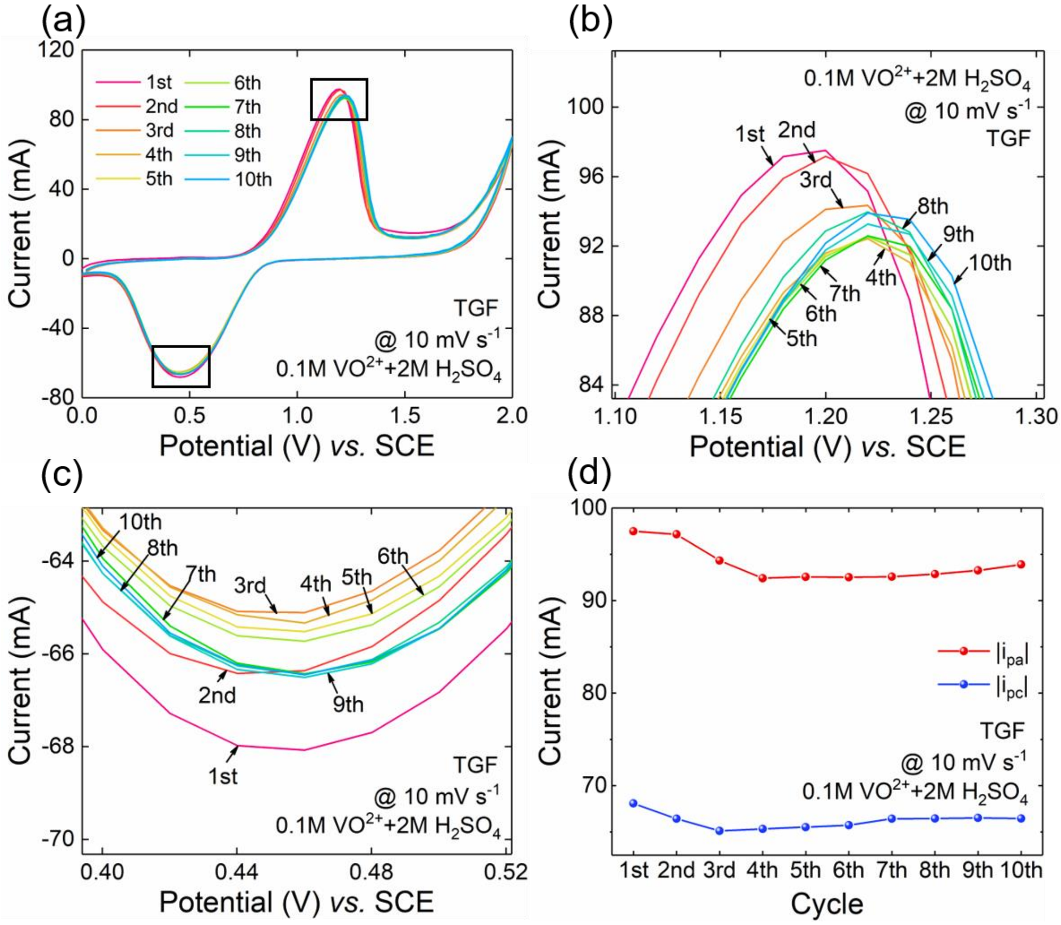

Supplementary Figure 32. (a) Long-term CV curves (1st to 10th cycles) of the TGF in the positive electrolyte of 0.1 M VO^{2+} and 2 M H_2SO_4 at a scan rate of 10 mV s^{-1} recorded by EW. (b) Enlarged view of oxidation currents in long-term CV curves (1st to 10th cycles). (c) Enlarged view of reduction currents in long-term CV curves (1st to 10th cycles). (d) The peak oxidation currents and the peak reduction currents of the CV curves in the first 10 cycles.

16. Intensity images in the first five cycles.

Supplementary Figure 33 shows the intensity images captured by the CCD camera in the first five CV cycles. It is visible that the bubbles gradually generate and then reach an equilibrium state.

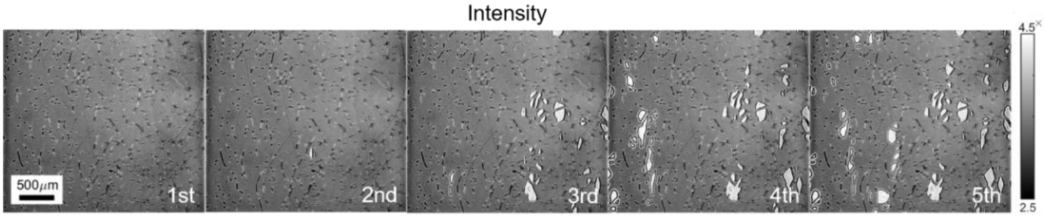

Supplementary Figure 33. Bubble generation as shown in the intensity images of the first five cycles.

17. The activity and reversibility distribution of the TGF in long-term CV.

To measure the activity and reversibility distribution of the TGF, the mappings of $|i_{pc}|$, $|i_{pc}/i_{pa}|$ and

$|\Delta E|$ are displayed in Supplementary Figure 34(a - c). The increasing $|i_{pc}|$ along with the raising cycle indicates the activity enhancement of the TGF. Besides, the decreasing $|i_{pc}/i_{pa}|$ and the increasing $|\Delta E|$ with the raising cycle reveal the reversibility worsening of the TGF.

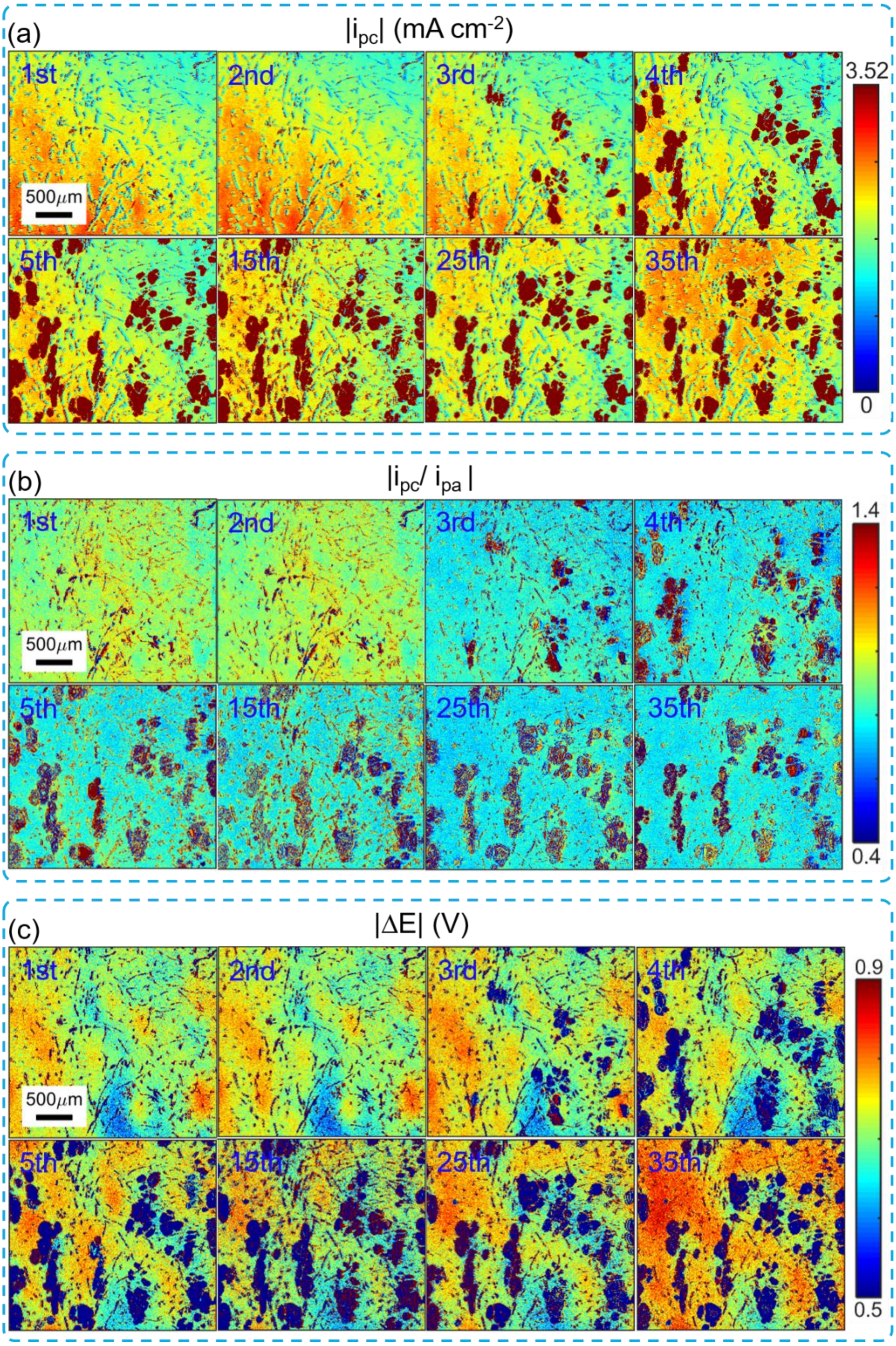

Supplementary Figure 34. (a) The mappings of the peak reduction current densities $|i_{pc}|$ of TGF electrode in

different cycles. (b) The mappings of $|i_{pc}/i_{pa}|$ in different cycles. (c) The mappings of the peak potential separation value $|\Delta E|$ in different cycles.

18. Region of interest for activity and reversibility distribution.

To exclude the fiber contact regions with low reflected intensity for its absence from the measurement range with high sensitivity, we choose the range of 1.54 - 3.41 mA cm^{-2} for $|i_{pa}|$, 1.43 - 2.926 mA cm^{-2} for $|i_{pc}|$, 0.70 - 1.21 for $|i_{pc}/i_{pa}|$ and 0.56 - 0.90 V for $|\Delta E|$ to further be divided into 17 intervals, respectively. For example, the distribution of the whole $|\Delta E|$ range for the PGF is shown in Supplementary Figure 35. The distribution in the dashed rectangle is the region of interest excluding the fiber contact area.

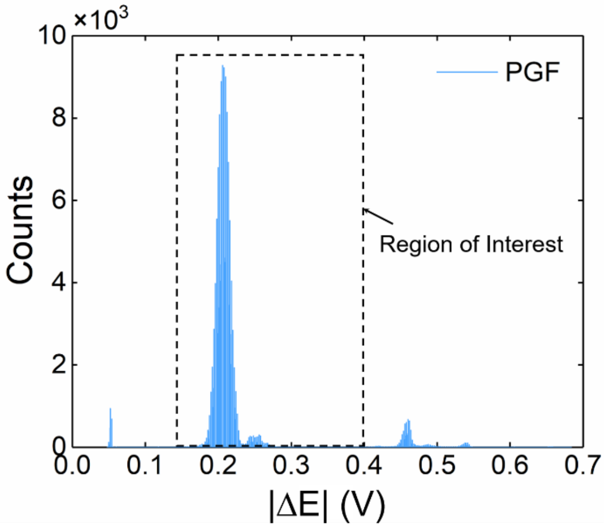

Supplementary Figure 35. Distribution of the peak potential separation value $|\Delta E|$ in the whole range for PGF. Dashed rectangle is the region of interest excluding the fiber contact area.

19. Comparison of the mappings of 17 intervals in region of interest.

Supplementary Figure 36 shows 17 $|i_{pa}|$ mappings of the 1st cycle in region of interest. The interval range and its number of $|i_{pa}|$ points are labelled in the bottom-right of each image. These images demonstrate the characteristic of the distinct regional distribution. The similar phenomenon appears in the $|i_{pc}|$ mappings in region of interest as shown in Supplementary Figure 37. It is indicated that $|i_{pa}|$ or $|i_{pc}|$ distribution may be utilized to represent the activity distribution. As for the $|i_{pc}/i_{pa}|$ distribution, 17 $|i_{pc}/i_{pa}|$ mappings of the 1st cycle in Supplementary Figure 38 do not have distinct regional distributions. However, the $|\Delta E|$ mappings of the 1st cycle with distinct regional distributions in Supplementary Figure 39 are appropriate for mapping the reversibility distribution.

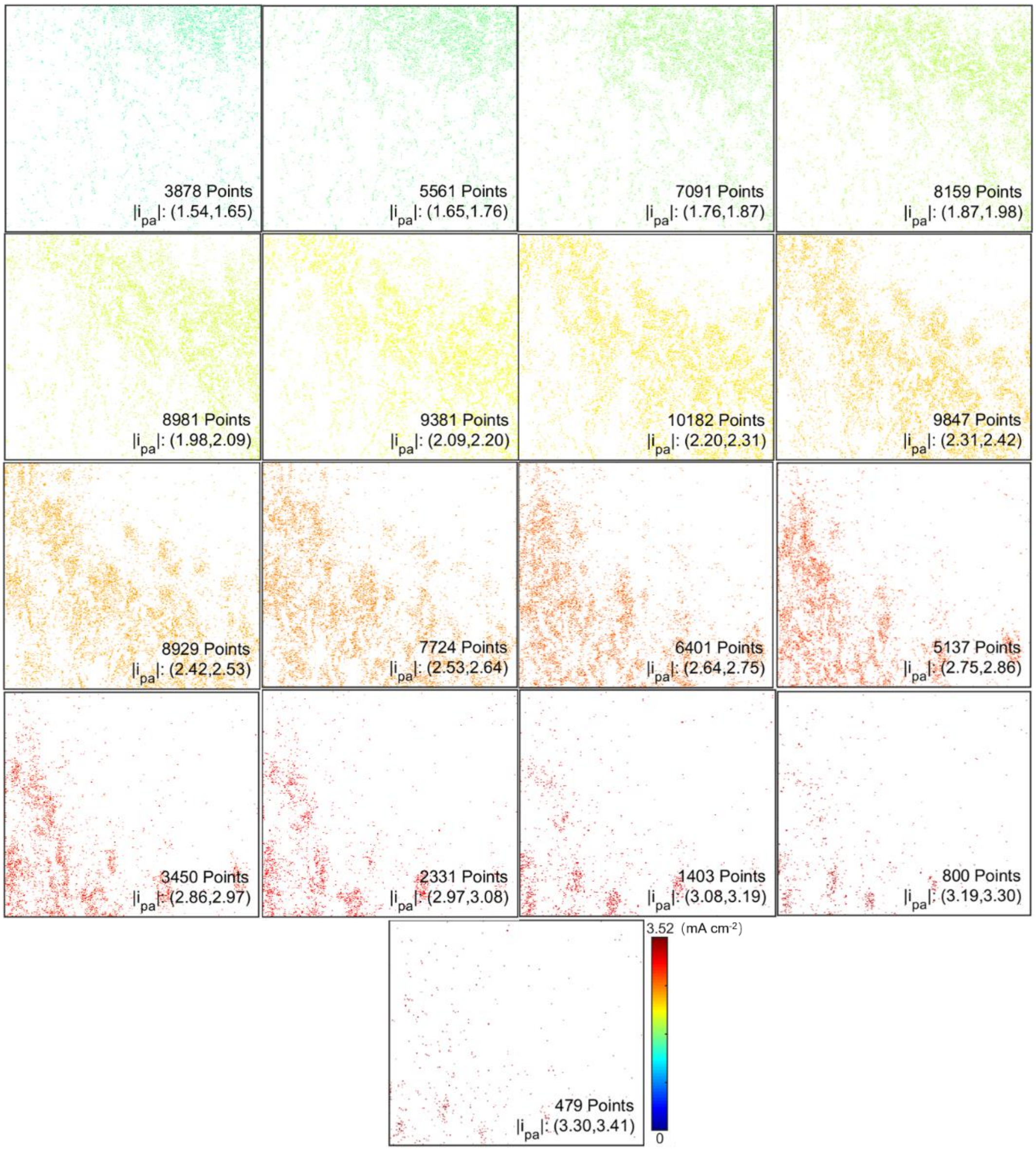

Supplementary Figure 36. The detailed $|i_{pa}|$ mappings of 17 intervals excluding the fiber contact regions at the 1st cycle.

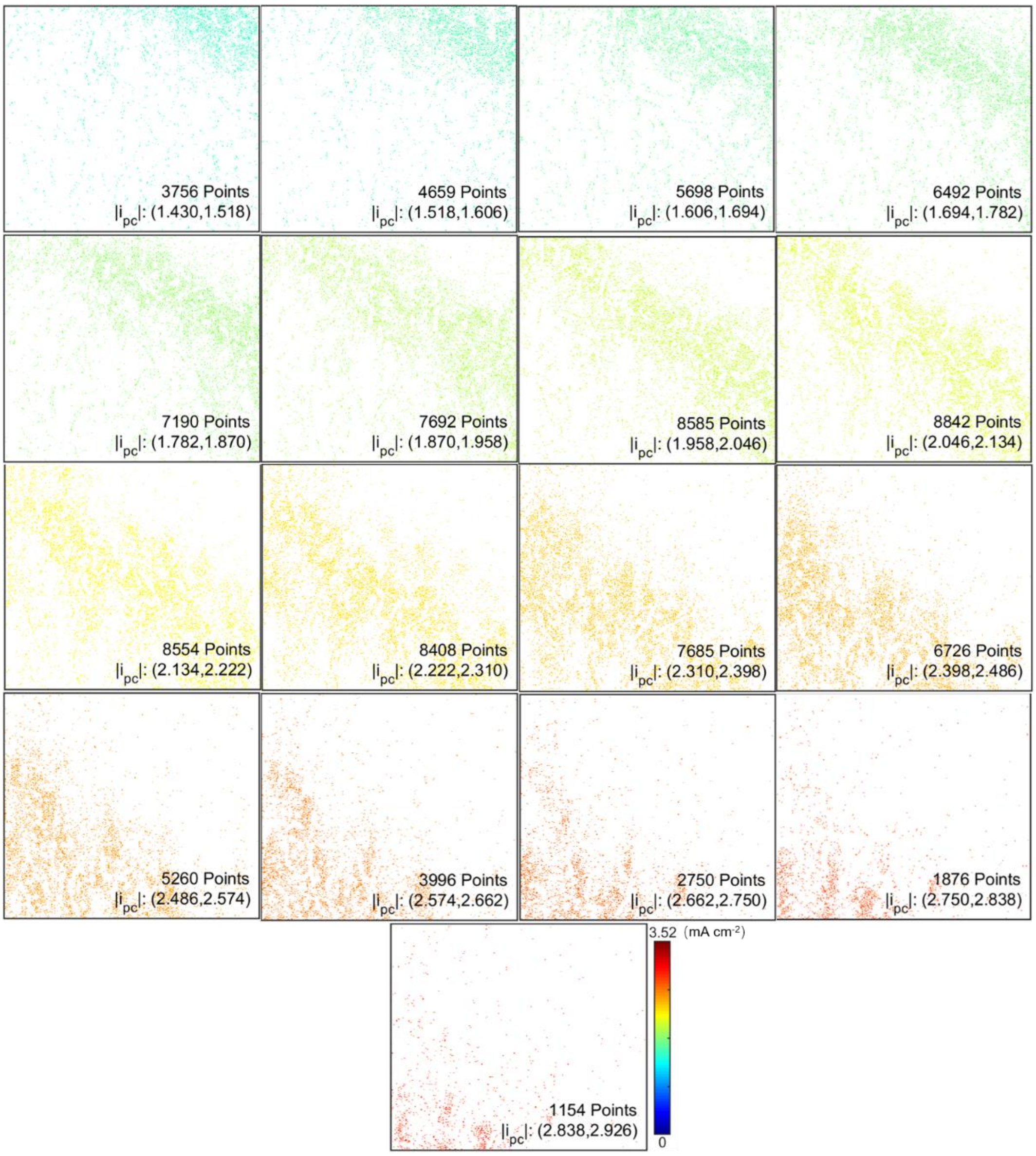

Supplementary Figure 37. The detailed $|i_{pc}|$ mappings of 17 intervals excluding the fiber contact regions at the 1st cycle.

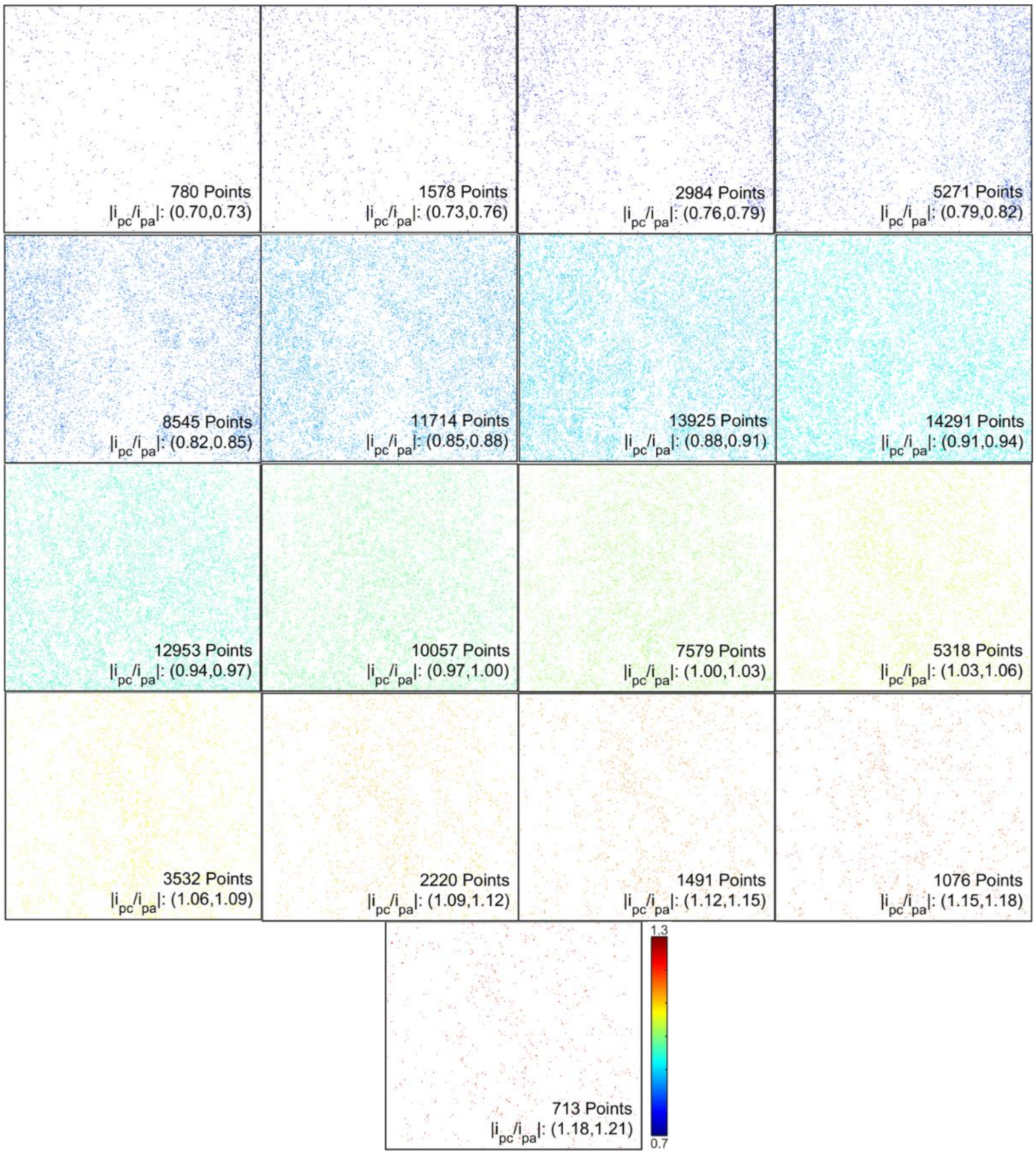

Supplementary Figure 38. The detailed $|i_{pc}/i_{pa}|$ mappings of 17 intervals excluding the fiber contact regions at the 1st cycle.

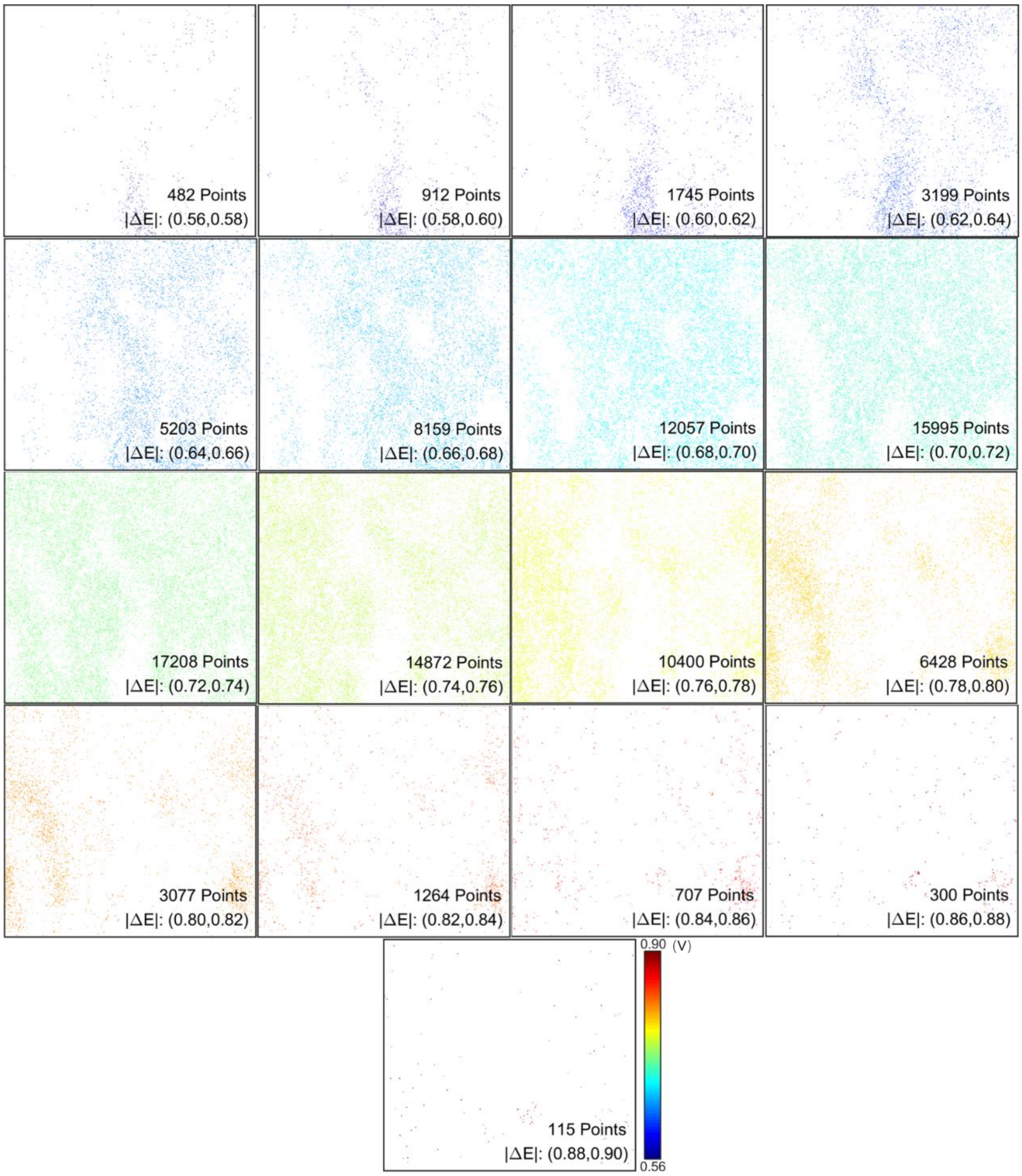

Supplementary Figure 39. The detailed |ΔE| mappings of 17 intervals excluding the fiber contact regions at the 1st cycle.

20. Probability of bubbles.

Supplementary Figure 40(a) shows the counts of points covered by bubbles and electrolyte in each $|i_{pc}|$ interval and the peak position of $|i_{pc}|$ is about 465. By calculation, the probability of bubbles versus $|i_{pc}|$ is displayed in Supplementary Figure 40(b) with the rising tendency. It indicates that the regions with high activity have larger probability of bubble generation. But the correlation is not significant (correlation coefficient: 0.8338). Supplementary Figure 40(c) shows the counts of points covered by bubbles and electrolyte in each $|i_{pc}/i_{pa}|$ interval and the peak position of $|i_{pc}/i_{pa}|$ is about

0.91. By calculation, the probability of bubbles versus $|i_{pc}/i_{pa}|$ is displayed in Supplementary Figure 40(d). Similarly, the correlation is also not significant (correlation coefficient: -0.7856).

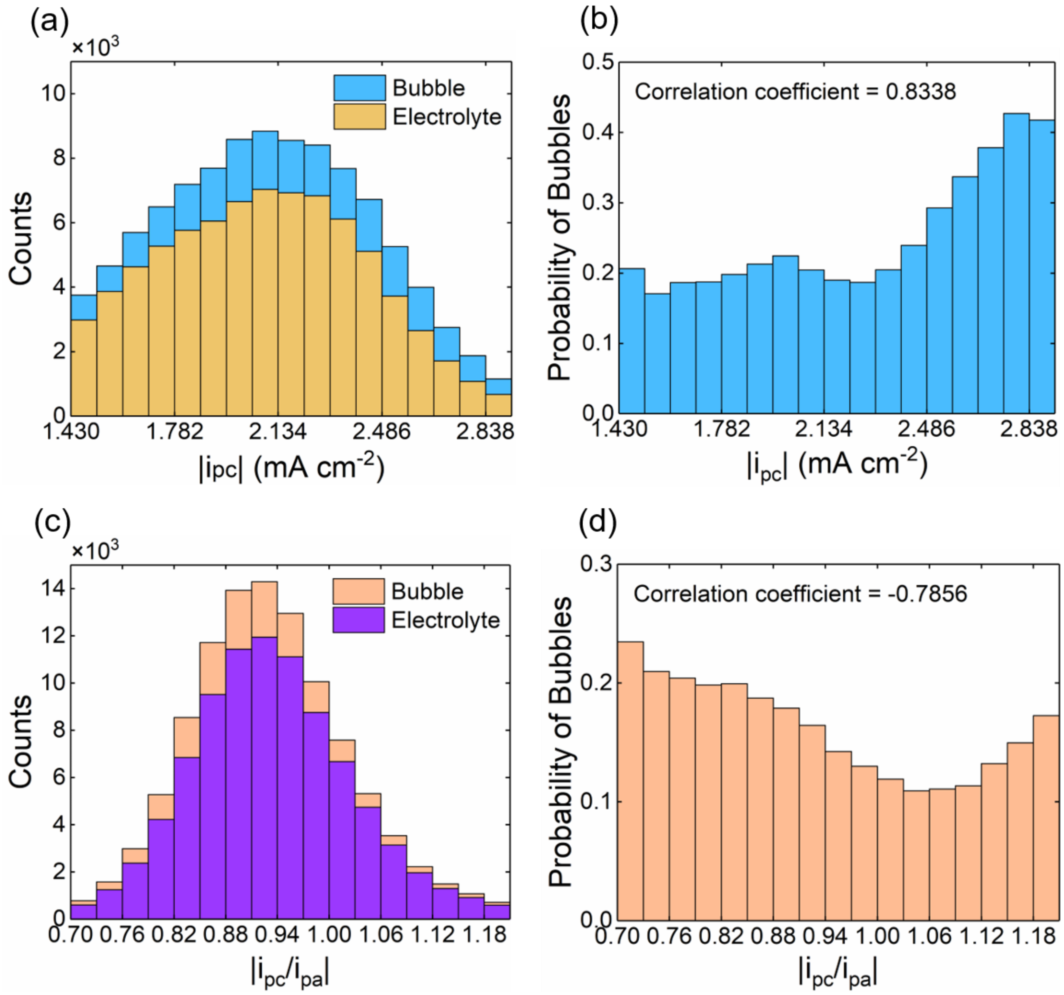

Supplementary Figure 40. (a) The counts of points covered by bubbles and electrolyte in 17 $|i_{pc}|$ intervals. (b) The probability of bubble generation in different $|i_{pc}|$ intervals. (c) The counts of points covered by bubbles and electrolyte in 17 $|i_{pc}/i_{pa}|$ intervals. (d) The probability of bubble generation in different $|i_{pc}/i_{pa}|$ intervals.

21. Bubble generation on the electrode (TGF + PGF) after the long-term CV.

Supplementary Figure 41 shows the bubble generation on the electrode (TGF + PGF) after the long-term CV. Almost all bubbles grow on the PGF instead of the TGF owing to the PGF with higher activity rather than the TGF. It indicates that bubbles are more like to generate on the electrode with higher activity.

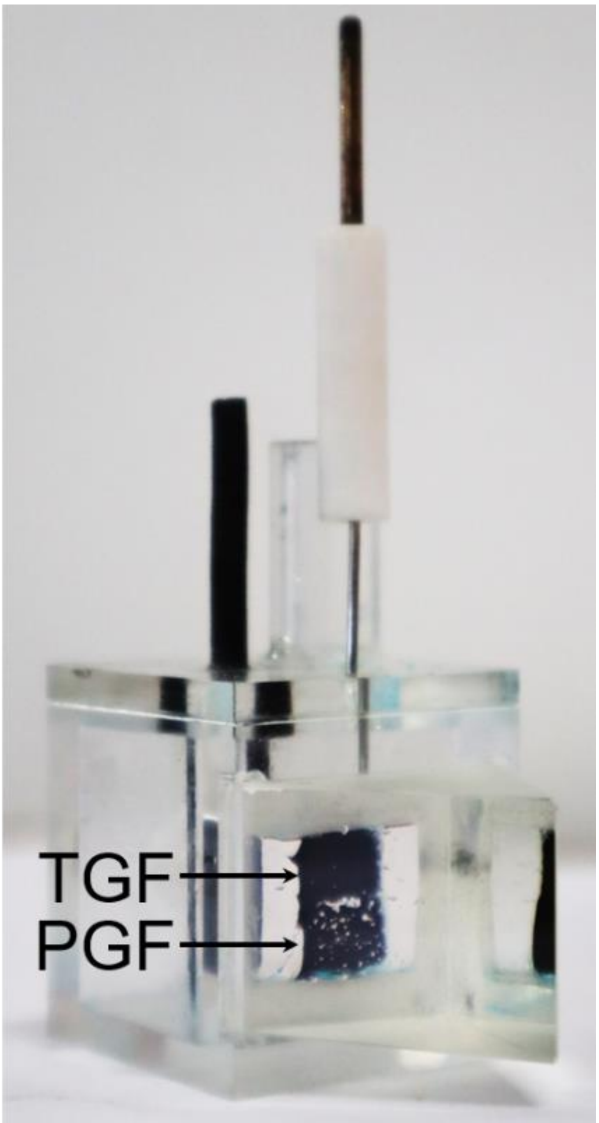

Supplementary Figure 41. Photograph of the electrode (TGF + PGF) in the reservoir-coupled sensor module after the long-term CV.

22. Bubble generation process in the first five cycles at different scan rates.

Supplementary Figure 42 shows the intensity images captured by the CCD camera in the first five CV cycles at different scan rates. It is faster to generate bubbles when the scan rate is lower as shown in Supplementary Figure 42. The CV curves of the TGF recorded by the EM at different scan rates are plotted in Supplementary Figure 43(a). It is found that the oxidation and reduction current densities of the vanadium ions raise sharply along with the increasing scan rate while the current density of the OER has a very small increase along with the increasing scan rate. The counts of bubbles generated by the OER in the first five cycles at different scan rates are plotted in Supplementary Figure 43(b). When the scan rate is small, the oxidation and reduction current

density of the vanadium ions is small while the bubble generation of the OER is fast and vice versa. This attributes to the enough current density of the OER and its longer duration time at lower scan rate.

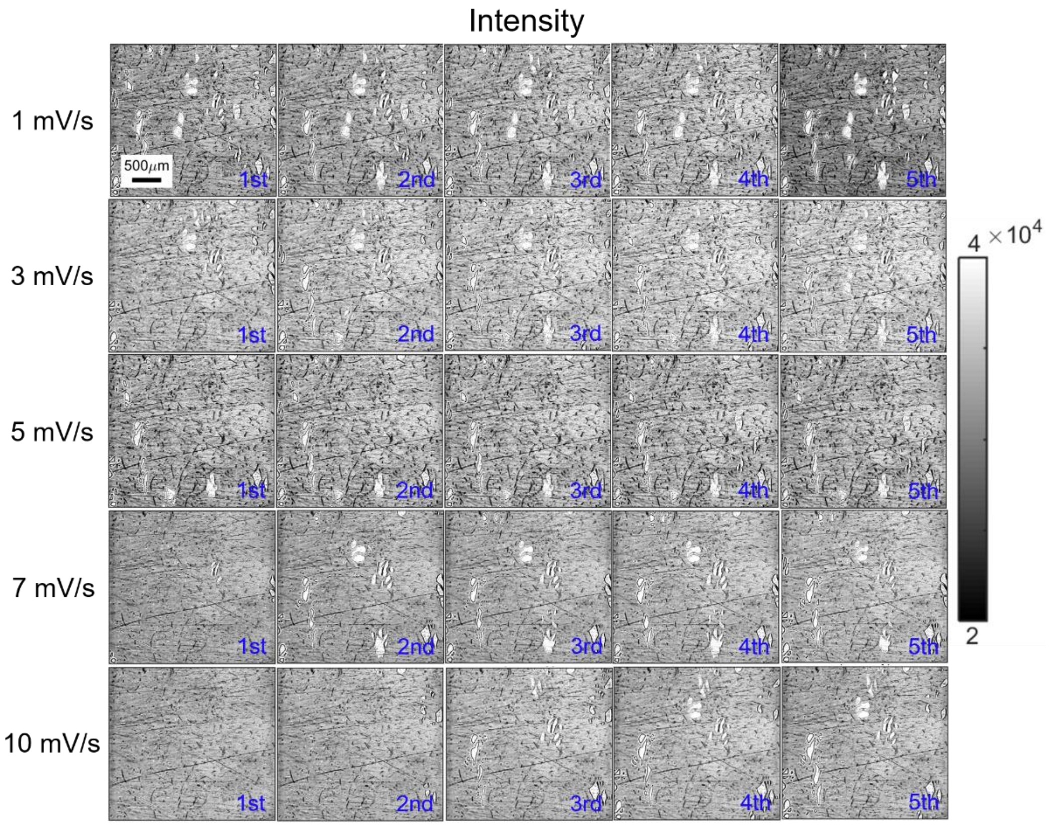

Supplementary Figure 42. Comparison of bubble generation process in the first five cycles at different scan rates.

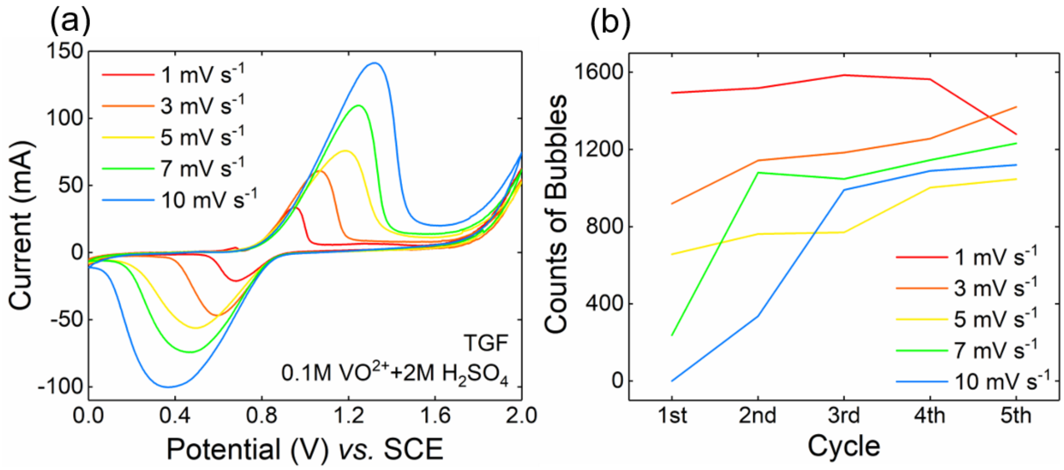

Supplementary Figure 43. (a) CV curves of the TGF in the positive electrolyte of 0.1 M VO^{2+} and 2 M H_2SO_4 at different scan rates recorded by the EM. (b) The counts of bubbles at different scan rates in first five cycles.

23. Cyclic voltammetry detection of the TGF in the positive electrolyte with 2 M H_2SO_4.

Supplementary Figure 44(a) shows the current density versus the sweep potential of the TGF in the positive electrolyte with 2 M H_2SO_4 by the EW. Supplementary Figure 44(b) shows the corresponding current density versus the sweep potential by the TIRi sensor. The similarity of the CV curves with the v infinitesimal current detected by the EW and TIRi sensor confirms that no background current is introduced in the detection by the TIRi sensor.

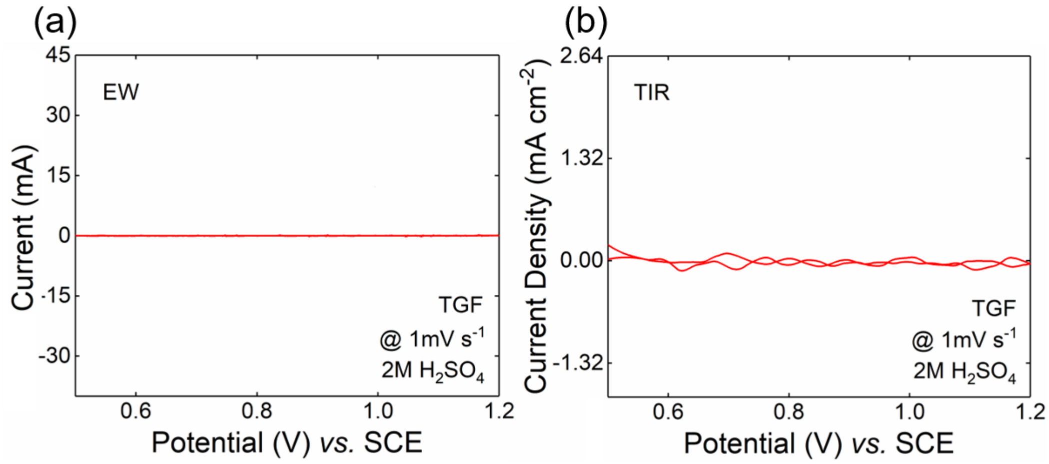

Supplementary Figure 44. CV curves of the TGF in the positive electrolyte with 2M H_2SO_4 detected by the (a) EW and (b) TIRi sensor.

24. Photograph of the TIRi sensor system for the VFB's electrode detection.

Supplementary Figure 45(a) displays the whole TIRi sensor system, including the incident light module (P), reservoir-coupled sensor module (G), imaging lens (H) and CCD camera (I). The enlarged view of the sensor module is shown in Supplementary Figure 45(b), in which the platinum electrode (G7) is the working electrode, the graphite rod (G8) is the counter electrode and the saturated calomel electrode (G9) is the reference electrode. Supplementary Figure 45(c) shows the contact state between the prism (G2) and the graphite felt (G3) connected with the platinum electrode. The graphite felt is immersed in the electrolyte held by the fluid reservoir (G5).

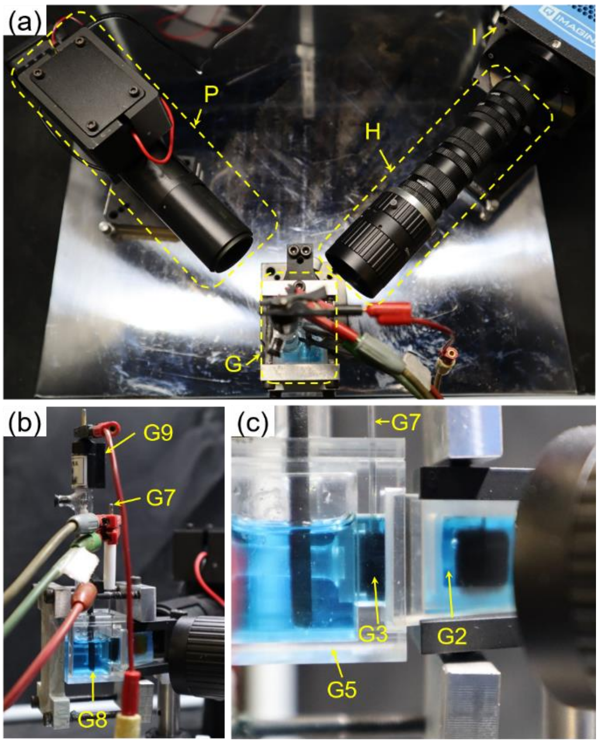

Supplementary Figure 45. (a) Photograph of the TIRi sensor system for the VFB's electrode detection. P: incident light module; G: reservoir-coupled sensor module (RCS); H: imaging lens; I: CCD camera. (b) Enlarged view of the RCS. G7: platinum electrode; G8: graphite rod; G9: saturated calomel electrode. (c) Further enlarged view of the RCS with graphite felt contact. G2: prism; G3: graphite felt; G5: fluid reservoir; G7: platinum electrode.

25. Reflectivity comparison of p and s polarized light for the TIRi sensor in theory.

Supplementary Figure 46 shows the angular spectrum of p and s polarized light for the TIRi sensor in theory. When the incident angle is larger than the angle of the total internal reflection θ_{TIR}, the total internal reflection occurs and the reflectivity is 1 for p and s polarized light. If the incident angle is smaller than θ_{TIR}, the reflectivity sharply decreases and the decrease rate of the p polarized light is larger than that of the s polarized light. It means that the reflectivity of the p polarized light is more sensitive than that of s polarized light at the incident angle near θ_{TIR}. Hence, the p polarized light with higher sensitivity is chosen in this work as the incident light of the TIRi sensor.

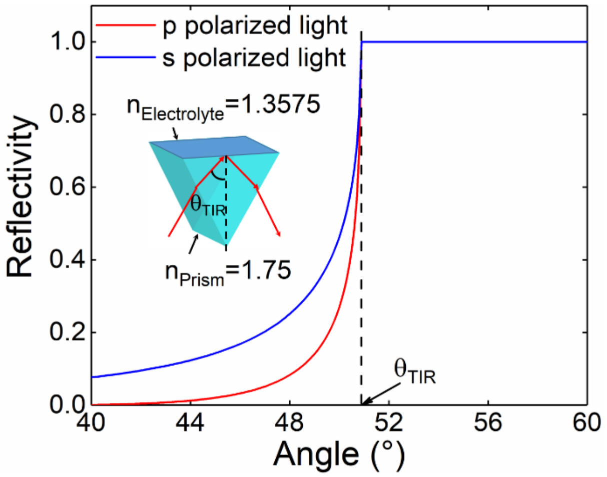

Supplementary Figure 46. Angular spectrum comparison of p and s polarized light for the TIRi sensor. Note: $n_{\mathrm{Electrolyte}}$ is the refractive index of the positive electrolyte of 0.1 M VO^{2+} and 2 M H_2SO_4 detected by Abbe refractometer. n_{Prism} is the refractive index of the prism. θ_{TIR} is the angle of total internal reflection.

26. The contact structure between the platinum electrode and the graphite felt.

As shown in Supplementary Figure 47, the platinum electrode was inserted from the middle of one side and through the graphite felt as the working electrode. According to the previous work of our group[5], compared with the conventional contact structure between the titanium (Ti) plate and the graphite felt, the contact structure between the platinum (Pt) electrode and the graphite felt mentioned above has some advantages: (1) as the current collector, the Pt wire has few active areas for the small contact area (0.314 cm^2), which has less impact on CV test. (2) the conductivity and corrosion resistance of Pt is preferable in acidic electrolyte. Besides, the contact structure can provide relatively uniform current distributions over the graphite felt for the high conductivity. In this paper, we conducted experiments based on the above contact structure.

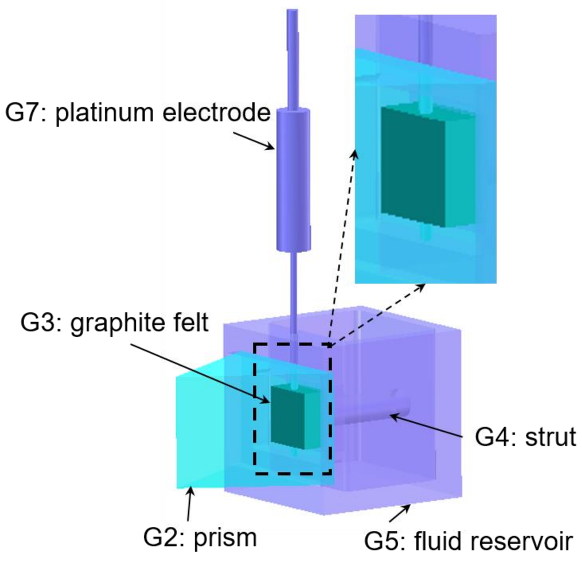

Supplementary Figure 47. Schematic of the contact structure between the platinum electrode and the graphite felt.

27. Supplementary online video

Supplementary Video 1 The entire electrochemical reaction and OER process on the TGF electrode with positive electrolyte (0.1 M VO^{2+} and 2 M H_2SO_4) are driven by a sweep potential between 0 V and 1.7 V at a scan rate of 10 mV s^{-1}. Supplementary Video 1 demonstrates the intensity and the time-varying counts of bubbles with gradually increase accompanied by periodic fluctuations in different cycles.

Supplementary Video 2 The entire electrochemical reaction and OER process on the TGF electrode with positive electrolyte (0.1 M VO^{2+} and 2 M H_2SO_4) by a sweep potential between 0 V and 1.7 V at a scan rate of 10 mV s^{-1}. The CV curves (top left in Supplementary Video 2) display the oxygen evolution/reduction reaction of 2 M H_2SO_4 electrolyte on the TGF driven by a potential sweep between 0 V and 1.7 V at a scan rate of 10 mV s^{-1}. The combined image (top right in Supplementary Video 2) combines the $|i_{pa}|$ mapping of the 1st cycle and the bubble covered regions of the average intensity image (blue areas). The enlarge views of two dashed rectangles are displayed in bottom left and right of Supplementary Video 2, which show the time-varying counts of bubbles with gradually increase accompanied by periodic fluctuations. Figure 4(c) in the manuscript is the snapshots from the top right in Supplementary Video 2.

Supplementary Video 3 The entire electrochemical reaction and OER process on the TGF electrode with positive electrolyte (0.1 M VO^{2+} and 2 M H_2SO_4) are driven by a sweep potential between 0 V and 2.0 V at a scan rate of 10 mV s^{-1}. Supplementary Video 3 demonstrates the intensity

and the time-varying counts of bubbles with gradually increase accompanied by dramatic variation in different cycles.

Supplementary Video 4 - 9 The time-varying concentration distributions of VO^{2+} in the CV of graphite felt (Supplementary Video 4), graphite film (Supplementary Video 5), graphite fibers 1 array (Supplementary Video 6), graphite fibers 2 array (Supplementary Video 7) in vertical dimension and graphite fibers (Supplementary Video 8), graphite fibers with different rates (Supplementary Video 9) in horizontal dimension.

Supplementary References

1. Bard, A. J., Faulkner, L. R. *Electrochemical methods: fundamentals and applications*, 2nd edn. Wiley, New York (2001).

2. Shan, X., Patel, U., Wang, S., Iglesias, R., Tao, N. Imaging local electrochemical current via surface plasmon resonance. *Science* **327**, 1363-1366 (2010).

3. Yamamura, T., Watanabe, N., Yano, T., Shiokawa, Y. Electron-transfer kinetics of Np^{3+}/Np^{4+}, NpO_2^{+}/NpO_2^{2+}, V^{2+}/V^{3+}, and VO^{2+}/VO_2^{+} at carbon electrodes. *Journal of the Electrochemical Society* **152**, A830-A836 (2005).

4. Djurišić, A. B., Li, E. H. Optical properties of graphite. *Journal of Applied Physics* **85**, 7404-7410 (1999).

5. Wu, L., Wang, J., Shen, Y., Liu, L., Xi, J. Electrochemical evaluation methods of vanadium flow battery electrodes. *Phys. Chem. Chem. Phys.* **19**, 14708-14717 (2017).
