# Supplementary figures and images for: In situ mapping of activity distribution and oxygen evolution reaction in vanadium flow batteries
Source: Nat Commun. 2019 Nov 21;10:5286. doi: 10.1038/s41467-019-13147-9 (PMC6872572; doi:10.1038/s41467-019-13147-9)

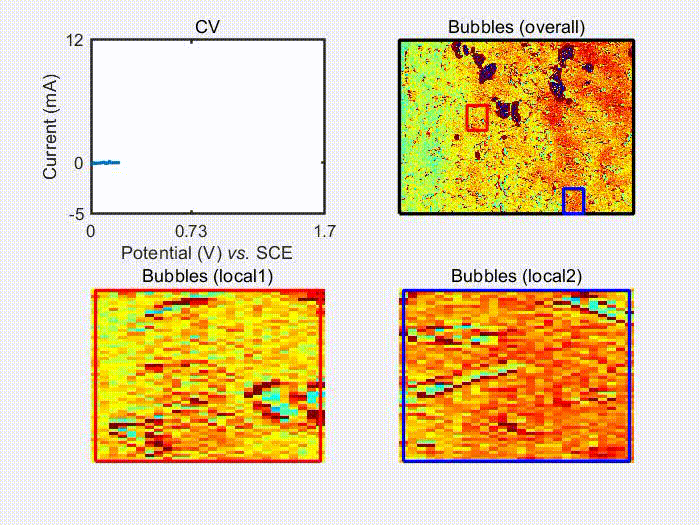

Supplement: Supplementary file 3 — Supplementary Video 2 [file 41467_2019_13147_MOESM3_ESM.gif]

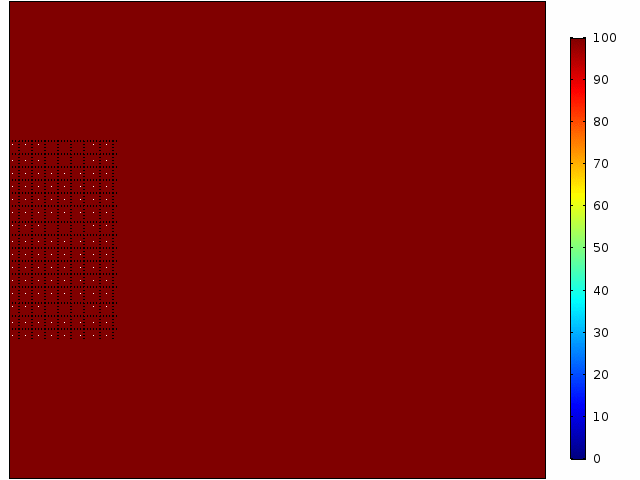

Supplement: Supplementary file 5 — Supplementary Video 4 [file 41467_2019_13147_MOESM5_ESM.gif]

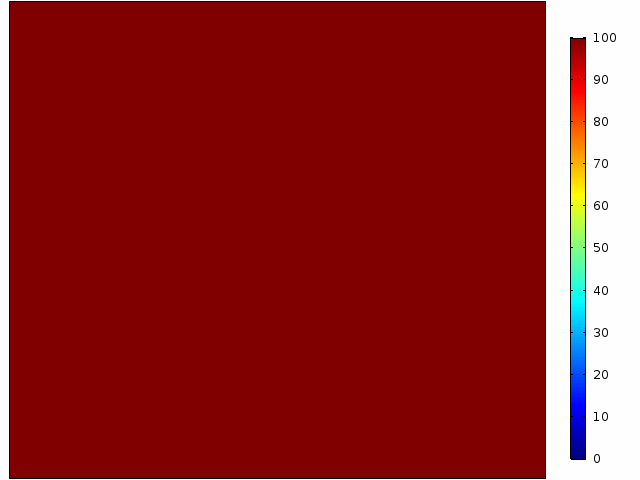

Supplement: Supplementary file 6 — Supplementary Video 5 [file 41467_2019_13147_MOESM6_ESM.gif]

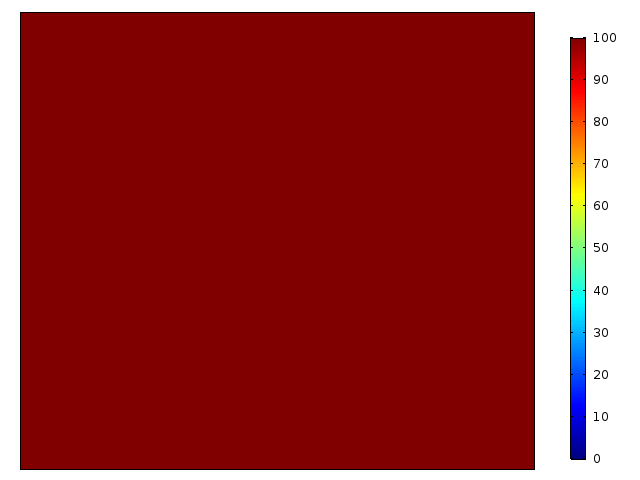

Supplement: Supplementary file 7 — Supplementary Video 6 [file 41467_2019_13147_MOESM7_ESM.gif]

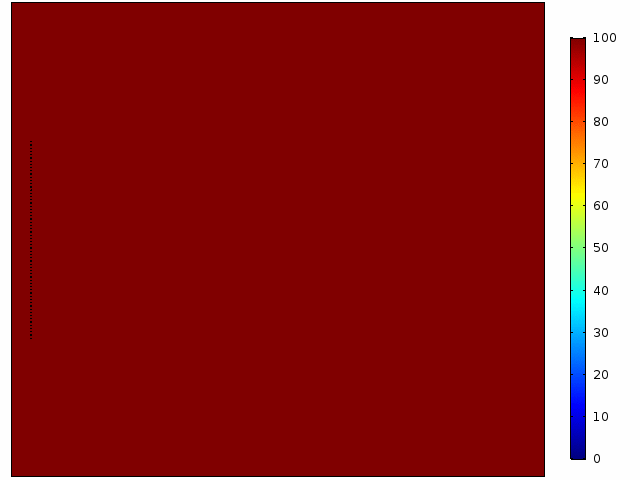

Supplement: Supplementary file 8 — Supplementary Video 7 [file 41467_2019_13147_MOESM8_ESM.gif]

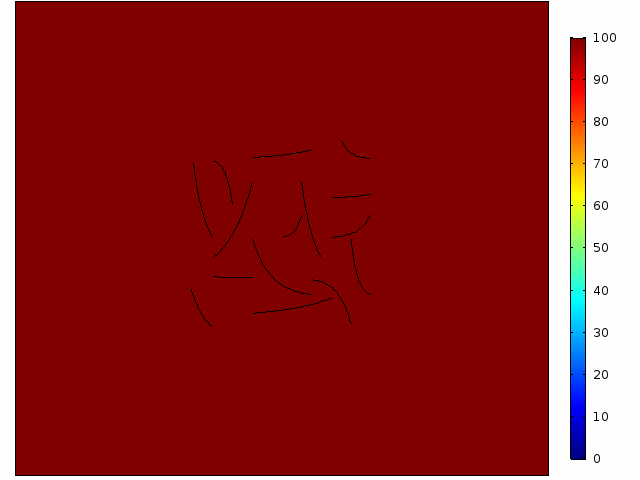

Supplement: Supplementary file 9 — Supplementary Video 8 [file 41467_2019_13147_MOESM9_ESM.gif]

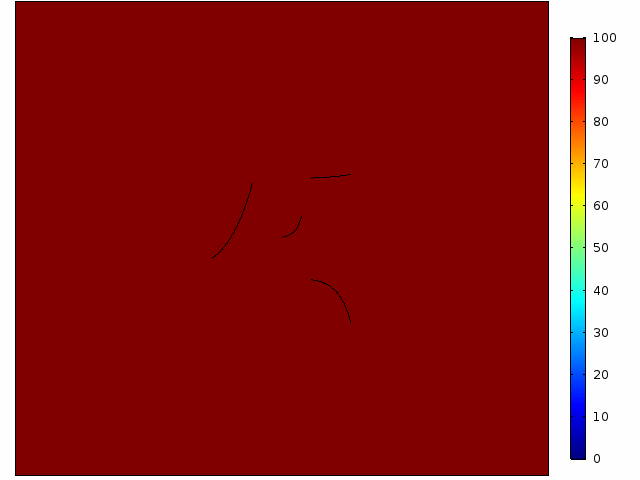

Supplement: Supplementary file 10 — Supplementary Video 9 [file 41467_2019_13147_MOESM10_ESM.gif]
